# Metabolomics analysis of plasma and adipose tissue samples from mice orally administered with polydextrose and correlations with cecal microbiota

**DOI:** 10.1038/s41598-020-78484-y

**Published:** 2020-12-09

**Authors:** Markku Tapani Saarinen, Olli Kärkkäinen, Kati Hanhineva, Kirsti Tiihonen, Ashley Hibberd, Kari Antero Mäkelä, Ghulam Shere Raza, Karl-Heinz Herzig, Heli Anglenius

**Affiliations:** 1 DuPont Nutrition & Biosciences, Global Health & Nutrition Science, Kantvik, Finland; 2 Afekta Technologies Ltd., Kuopio, Finland; 3 School of Pharmacy, University of Eastern Finland, Kuopio, Finland; 4 Institute of Public Health and Clinical Nutrition, University of Eastern Finland, Kuopio, Finland; 5 DuPont Nutrition & Biosciences, Genomics & Microbiome Science, St. Louis, MO USA; 6 Institute of Biomedicine, Medical Research Center (MRC), University of Oulu, and University Hospital, Oulu, Finland; 7 Department of Gastroenterology and Metabolism, Poznan University of Medical Sciences, Poznan, Poland

**Keywords:** Biochemistry, Microbiology, Physiology, Biomarkers

## Abstract

Polydextrose (PDX) is a branched glucose polymer, utilized as a soluble dietary fiber. Recently, PDX was found to have hypolipidemic effects and effects on the gut microbiota. To investigate these findings more closely, a non-targeted metabolomics approach, was exploited to determine metabolic alterations in blood and epididymal adipose tissue samples that were collected from C57BL/6 mice fed with a Western diet, with or without oral administration of PDX. Metabolomic analyses revealed significant differences between PDX- and control mice, which could be due to differences in diet or due to altered microbial metabolism in the gut. Some metabolites were found in both plasma and adipose tissue, such as the bile acid derivative deoxycholic acid and the microbiome-derived tryptophan metabolite indoxyl sulfate, both of which increased by PDX. Additionally, PDX increased the levels of glycine betaine and l-carnitine in plasma samples, which correlated negatively with plasma TG and positively correlated with bacterial genera enriched in PDX mice. The results demonstrated that PDX caused differential metabolite patterns in blood and adipose tissues and that one-carbon metabolism, associated with glycine betaine and l-carnitine, and bile acid and tryptophan metabolism are associated with the hypolipidemic effects observed in mice that were given PDX.

## Introduction

Dietary fiber (DF) and other dietary nutrients that are not digested during small intestinal transit are potential substrates for microbes found in the large intestine. Typically, DF is found in relatively low quantities in the Western diet (WD) but has well-established metabolic health benefits, including the promotion of weight loss and improved insulin sensitivity^1^. These indigestible substrates are metabolized into a wide range of compounds, such as short-chain fatty acids (SCFAs), organic acids, biogenic amines, vitamins, ammonia, hydrogen sulfide, and phenols, by the microbes that reside in the colon^2^. The metabolites that are produced by these microbes can feed other bacteria, causing shifts in the microbiota composition and the metabolome^3^, and may be utilized by the gastrointestinal tract itself, absorbed into the blood circulation and metabolized further by the host, or excreted in the urine^4^. The absorbed metabolites may have positive health effects, acting as signaling molecules, providing energy, or becoming integrated into other molecules^4,5^; however, they may also be toxic to the host, resulting in adverse health effects and predisposing the host to disease^6^.

Polydextrose (PDX) is a highly branched and randomly linked glucose polymer, which is widely used as a dietary fiber and a sugar and fat replacement^7^. PDX can also act as a prebiotic due to its promotion of beneficial gut microbes and the production of SCFAs^8,9^. As a complex molecule, PDX is resistant to hydrolysis by human digestive enzymes and is degraded slowly by intestinal microbes during gastrointestinal passage, making it available for fermentation by both proximal and distal colonic microbiota^10,11^. Finally, a proportion of PDX is excreted in feces^10,11^. The sustained fermentation of PDX throughout the colon also facilitates good tolerance in humans (90 g/d) because slow fermentation leads to limited gas production^12^.

The rapidly growing field of metabolomics has provided tools for the analysis of many hundreds of metabolites in complex specimens, including biofluids, tissues, and cells^13^. Recently, untargeted metabolomics analysis, employing ^13^C nuclear magnetic resonance (NMR) spectroscopy, was used to identify molecules that originate from PDX in an in vitro human colon simulation model. A study using ^13^C-labeled PDX showed that the major ^13^C-labeled metabolites in colon simulator samples were acetate, butyrate, propionate, and valerate. In addition, minor amounts of ^13^C-labeled lactate, formate, and succinate were detected^14^. Another in vitro human colon simulator study using PDX^15^ showed higher levels of acetate, butyrate, propionate, and succinate, and lower levels of certain amino acids, valerate, formate, isovalerate, and trimethylamine in PDX samples compared to control samples.

In contrast, a human clinical study examining PDX found no significant changes in any of these metabolites in fecal samples after PDX or placebo treatment; however, this result could be due to the absorption of metabolites by the intestine^16^. However, analysis of intestinal digesta has revealed that depolymerized PDX products can be found in human fecal samples^16^ and the most distal part of the colon in pigs^10^, providing further evidence of PDX fermentation in the colon.

Recently, a mouse model was used to study the effects of PDX supplementation on blood lipid levels, the modulation of the gut microbiota, and the expression of genes associated with lipid metabolism^17^. PDX demonstrated a positive, hypolipidemic effect after 14 days of feeding with a WD, and the observed alterations in the gut microbiota provided further evidence that certain bacteria are linked with a high-fat diet^18^. Altered gut microbiota were associated with the differential expression of genes associated with lipid metabolism, which are regulated by SCFAs^19^ and also associated with PDX^17^.

Encouraged by these findings, we extended our previous study^17^ to examine the metabolomics of mouse blood serum and epididymal fat tissue samples, to determine whether the hypolipidemic effects of PDX, changes in gene expression that are associated with lipid metabolism, and alterations in the gut microbiota could be observed at the molecular level.

## Results

### Body weight, food intake, plasma, and tissue triglycerides and cholesterol

As previously reported^17^, no significant changes were observed in mouse body weights over the two-week test period (*p* = 0.07), even though cumulative food intake was reduced significantly (*p* < 0.001) in the WD + PDX mice compared with WD mice. Fasting plasma TGs (50 ± 4.8 mg/dL vs. 81 ± 5.3 mg/dL; *p* < 0.001) and plasma cholesterol (141 ± 4 mg/dL vs. 161 ± 7 mg/dL, *p* < 0.05) were significantly reduced in WD + PDX mice compared with WD mice^17^. Furthermore, the epididymal fat weight of WD + PDX animals was significantly reduced (*p* < 0.05) compared with that of WD animals^17^.

### Metabolomic analysis of plasma and fat tissue samples

**Figure 1:**
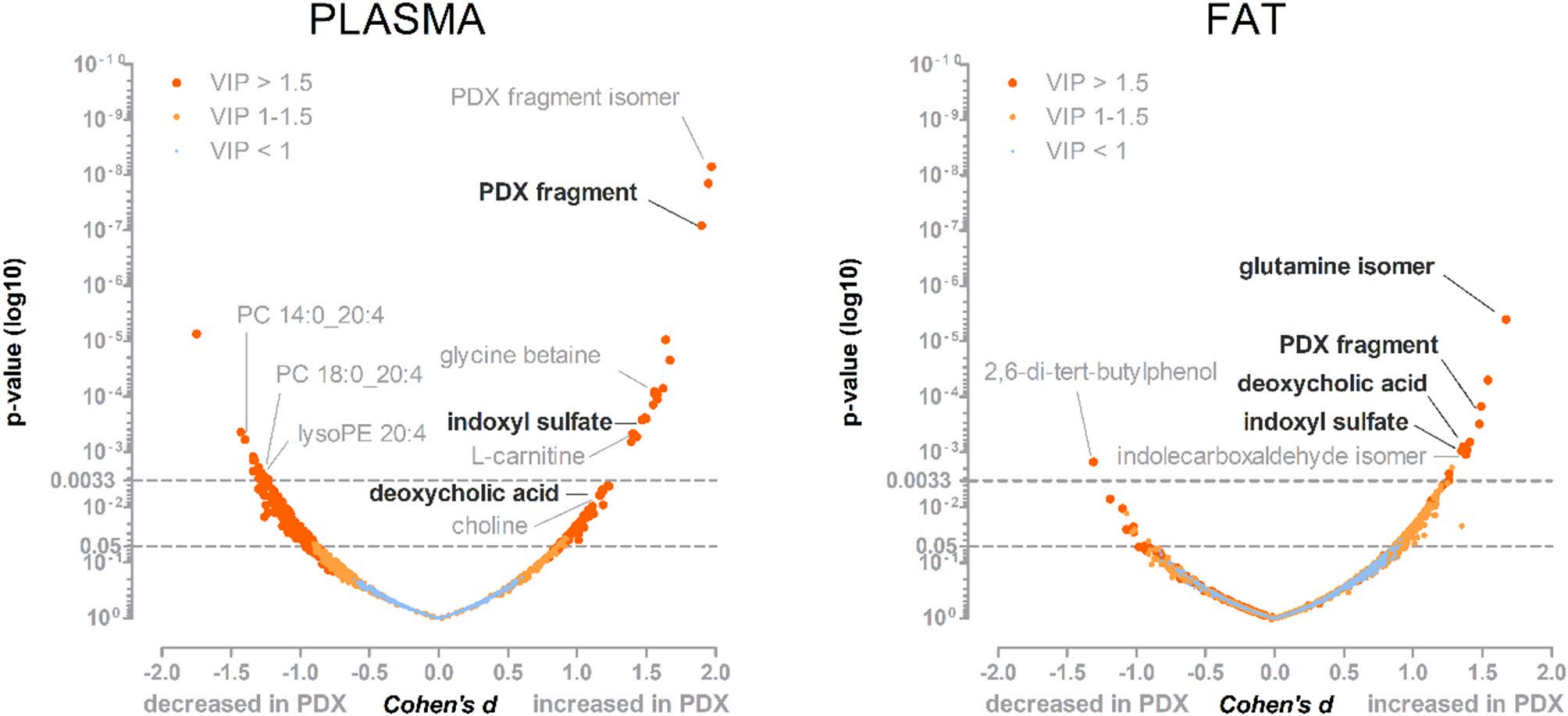
Overview of the metabolomics analysis of plasma and epididymal adipose tissue samples. Volcano plots showing the measured molecular features from plasma and fat tissue samples, which are indicated by the dots. *p* Values were calculated using Welch’s t-test, and 0.0033 was set as the adjusted α level (Bonferroni’s method). Cohen’s d values were calculated between PDX-treated and control animals, where a positive d-value indicated that the metabolite was identified at higher levels in PDX-fed animals and a negative d-value indicated higher levels in control animals. Variable importance to projection (VIP) values from the partial least square discriminant analysis (PLS-DA) are indicated by the different sizes and colors of the dot. The interesting molecular features are annotated in the figure. Legend: PC, phosphatidylcholine; PDX, polydextrose; lysoPE, lysophosphatidylethanolamine.

The only difference between the experimental diets was the inclusion of PDX, to ensure that changes in plasma and adipose tissue metabolites did not reflect differences in ingested foods or direct digestion products. A total of 1542 and 1607 molecular features were identified in the plasma and adipose tissue samples, respectively, and among them, 208 and 243 had *p* values below 0.05 (Fig. 1). In both sample types, 15 principal components were necessary to explain 95% of the variance in the metabolomics analysis. Therefore, we adjusted the α level to 0.0033 to account for multiple testing (Bonferroni’s method).

**Figure 2:**
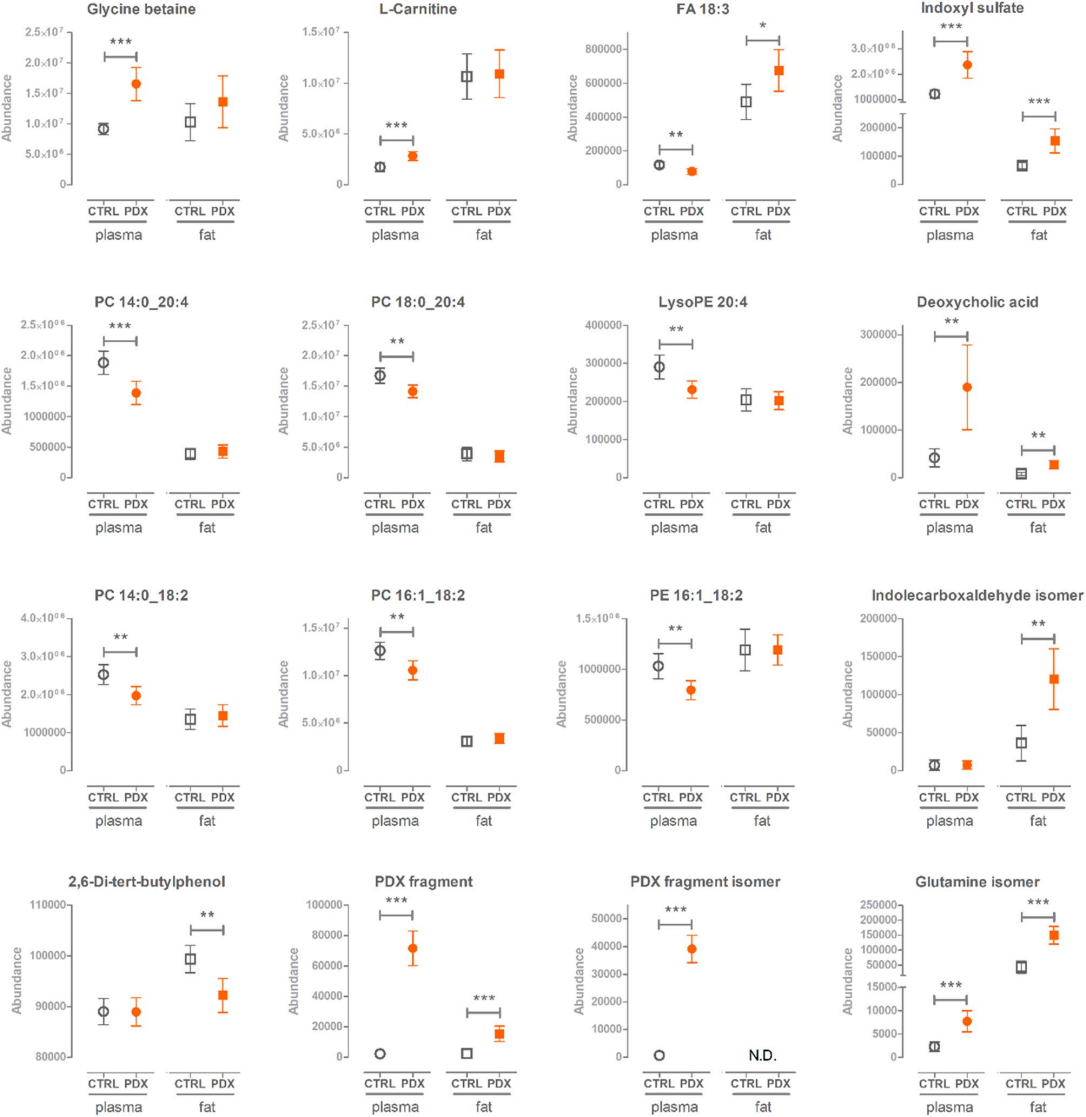
Significantly altered and identified metabolites from the metabolomics analysis. Metabolite abundances are shown for both plasma and fat tissue samples, from PDX-treated and control animals. Welch’s t-test was used to calculate *p* values. Legend: ***p < 0.001, **p < 0.01, *p < 0.05. FA, fatty acid; N.D., not detected; PC, phosphatidylcholine; PDX, polydextrose; PE, phosphatidylethanolamine.

The identified and significantly altered metabolites are shown in Fig. 2. The levels of indoxyl sulfate, deoxycholic acid, an isomer of glutamine, and a fragment representing a product of PDX degradation were increased in the WD + PDX group, in both plasma and fat tissue samples, compared with those in the WD group (Fig. 2). The PDX-treated animals had lower plasma levels of fatty acid (FA) 18:3 (which was identified α-linolenic acid), arachidonic acid (20:4), and linoleic acid (18:2), containing phospholipids (phosphatidylcholines [PCs], phosphatidylethanolamines [PEs] and lysoPEs, Fig. 2). In the adipose tissue samples, an increase in FA 18:3 (α-linolenic acid) levels (*p* = 0.0174) was observed.

Significant increases were found for the plasma levels of glycine betaine and l-carnitine in the PDX-treated group compared with controls (Fig. 2). A trend toward increased choline levels was also observed in the plasma samples from PDX-treated animals (*p* = 0.0049); however, the *p* value of this comparison was above the adjusted α level of 0.0033. These results indicated altered one-carbon metabolism in animals receiving PDX.

In the adipose tissues, a significant increase in the levels of an indolecarboxyaldehyde isomer was observed, which was identified as neither indole-4-carboxaldehyde nor indole-3-carboxaldehyde by the detailed MS/MS analysis. In the PDX group, we found decreased 2,6-di-tert-butylphenol levels compared with the control group (Fig. 2). Apart from these two molecular features, no additional compounds were identified from the fat tissue, with the statistical certainty of *p* < 0.0033. All detected molecular features as well as calculated *p* values, Cohen’s d values, and VIP values are presented in Supplementary Table 1.

### Pathway analysis

**Figure 3:**
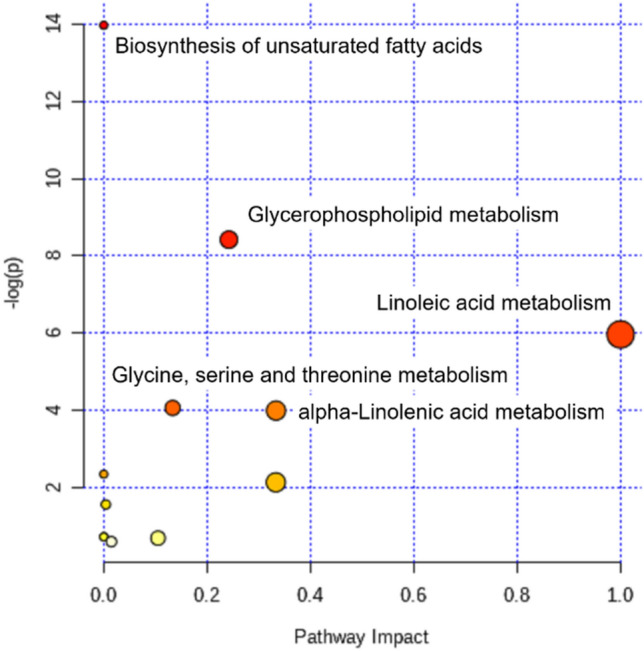
Pathway mapping with MetaboAnalyst, based on changes in metabolites, without adjustments for multiple testing and with *p* < 0.05. The Y-axis is the –log *p* values from the pathway enrichment analysis. The X-axis is the pathway impact values, from pathway topology analysis. The node colors and radii are based on *p* values and pathway impact values, respectively. Color intensities increase with reducing *p* values, and the sizes of the circles increase with increased pathway impacts.

When the data were analyzed using MetaboAnalyst, two metabolic routes were changed markedly, even after the *p* values were adjusted for multiple testing: unsaturated fatty acids and glycerophospholipid metabolism (Fig. 3).

### Correlation analysis of genus-level taxa and metabolomic data

Spearman’s correlation analyses were conducted to examine associations between genus-level taxa with > 0.1% abundance and metabolic features, using the R packages hmisc and gplots^20,21^ for those bacteria that differed according to treatment^17^. Relative abundance of caecal bacteria at genus level of taxonomy in WD and WD + PDX mice analyzed in the previous study is presented in Supplementary Figure 1^17^. The cecal microbiota analysis revealed several bacterial genera that were significantly different in the PDX-fed mice compared with the control mice, and many of these genera have been associated with benefits in lipid metabolism-related metabolic effects.

**Figure 4:**
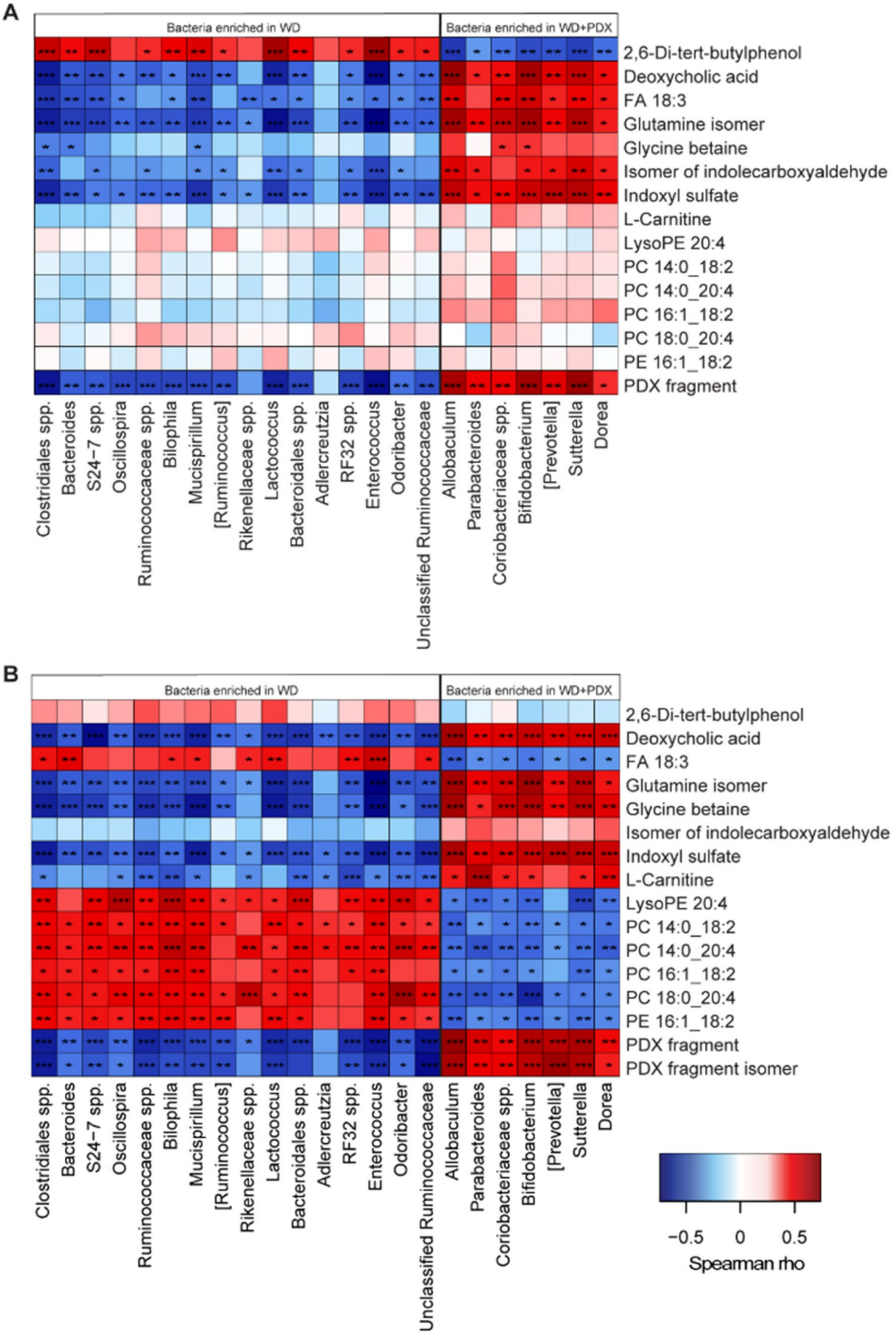
Heatmap showing the correlations among the relative abundance of cecal bacteria, at the genus level, and the metabolites identified in (**A**) adipose and (**B**) plasma. Bacteria are ordered by those that were enriched in the control Western diet (WD)-fed mice or in the WD diet-fed mice administered polydextrose (PDX). ***p < 0.001, **p < 0.01, *p < 0.05.

Briefly, they included significant increases in the relative abundance of *Allobaculum*, *Bifidobacterium,* and *Coriobacteriaceae* and decreases in the relative abundance of members of *Clostridiales* spp. and the families Ruminococcaceae, Rikenellaceae, Desulfovibrionaceae, and Deferribacteraceae. The bacterial genera enriched in PDX-treated mice generally correlated better with the metabolites in plasma than in adipose-tissue (Fig. 4B), and all, except *Prevotella*, correlated roughly with the same metabolites. In adipose tissue (Fig. 4A), fewer correlations were observed between the bacteria enriched by the PDX group and the metabolites, and some differences were identified between genera, such as *Parabacteroides* not correlating with FA 18:3 (α-linolenic acid) and *Coriobacteriaceae* spp. and *Bifidobacterium* being the only species to correlate with glycine betaine.

A notable difference between plasma and epididymal adipose tissue samples during the correlation analysis (Fig. 4) was that phospholipids, such as lysophosphatidylethanolamine and various phosphatidylcholines, correlated negatively with the bacterial genera that were enriched by the PDX diet in plasma (Fig. 4B), whereas no correlations were observed when measured from the adipose tissue (Fig. 4A). However, FA 18:3 (α-linolenic acid) showed increased concentrations and a positive correlation with these bacterial genera in adipose tissue but not in plasma.

Positive correlations were seen with deoxycholic acid, the glutamine isomer, and indoxyl sulfate, in both adipose tissue and plasma, for the genera that were enriched in PDX-fed mice. l-carnitine correlated significantly with all PDX-enriched bacteria, except *Prevotella*, in plasma but not in adipose tissue. Similarly, glycine betaine, which is associated with l-carnitine metabolism, correlated significantly with all genera enriched by PDX in the plasma but only with *Coriobacteriaceae* and *Bifidobacterium* in adipose tissue. Furthermore, in adipose tissue only, a negative correlation between 2,6-Di-tert-butylphenol and genera enriched by PDX and 2,6-Di-tert-butylphenol was observed.

### Correlation analyses between fasting plasma triglycerides and total cholesterol and metabolomic data

The plasma TG and total cholesterol values from the original study^17^ were correlated with the significantly different metabolome data, using an α level of 0.0033, which was adjusted to account for multiple testing.

**Figure 5:**
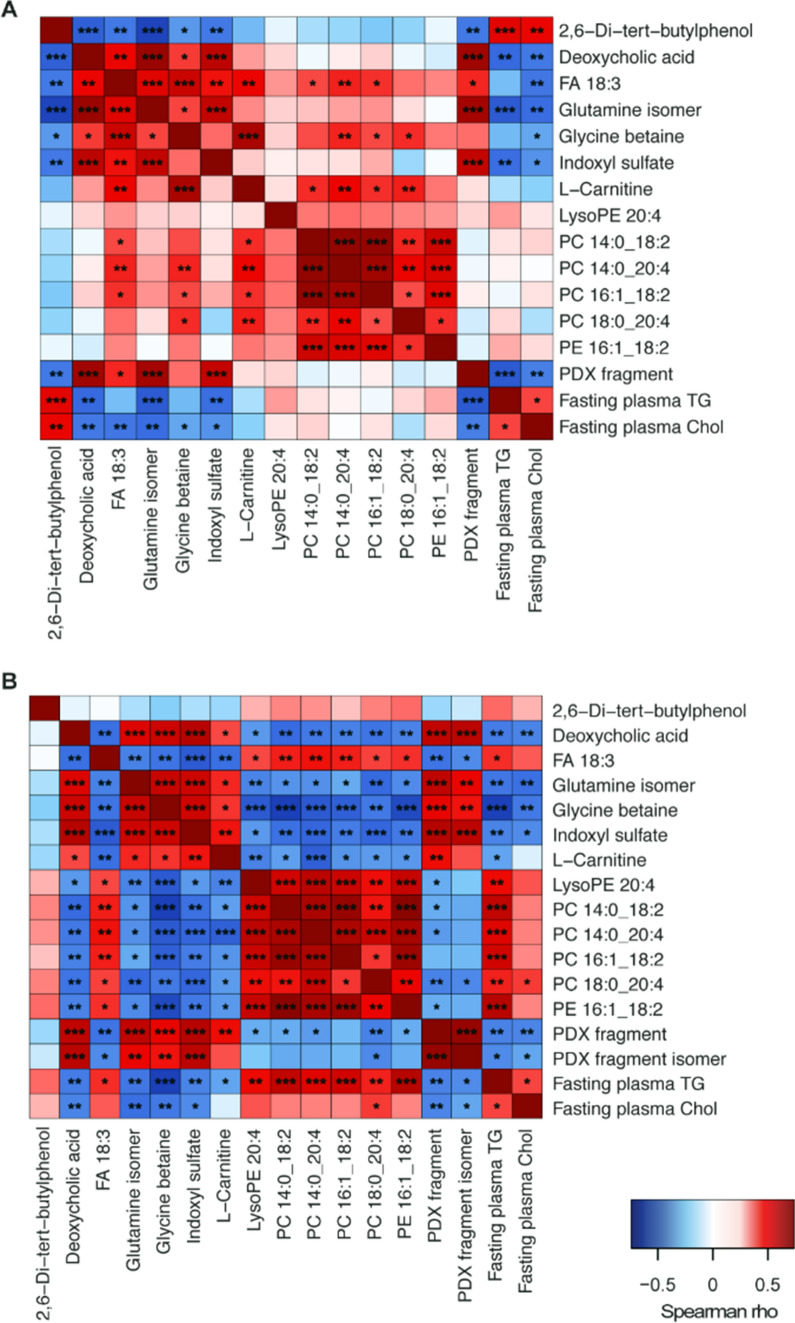
Heatmap showing correlations between fasting plasma TG and total cholesterol levels and metabolites in (**A**) adipose and (**B**) plasma, for both control Western diet (WD)-fed mice WD diet-fed mice administered polydextrose (PDX). ***p < 0.001, **p < 0.01, *p < 0.05.

In both adipose tissue and the plasma metabolome (Fig. 5), three metabolites correlated negatively with TG and total cholesterol values: deoxycholic acid, the glutamine isomer, and indoxyl sulfate. In addition, fasting total cholesterol correlated negatively with glycine, but a negative correlation with fasting TG was only noted in plasma. In adipose tissue (Fig. 5A), a positive correlation with both TG and total cholesterol was observed for 2,6-Di-tert-butylphenol, and no other positive correlations were noted. FA 18:3 correlated negatively with fasting total cholesterol in the adipose tissue metabolome but not in the plasma metabolome. In the plasma metabolome (Fig. 5B), positive correlations with PC 18:0_20:4 and both fasting TG and total cholesterol were observed. Additionally, fasting plasma TG correlated positively with phosphatidylcholines (PC 14:0_18:2, PC 14:0_20:4, PC 16:1_18:2), LysoPE 20:4, PE 16:1_18:2, and FA 18:3, in the plasma metabolome, whereas a negative correlation was found with l-carnitine.

## Discussion

The supplementation of the WD with PDX added an indigestible polysaccharide, with fiber-like and prebiotic characteristics^9^, and its fermentation in the colon produces SCFAs, especially acetate, butyrate, and propionate^10,22,23^ that can be absorbed into blood circulation^10^. PDX provides a saccharolytic, fermentable substrate throughout the colon, from proximal to distal, which has been suggested to direct fermentation toward the formation of SCFAs, instead of towards the metabolites generated during protein fermentation, such as branched-chain fatty acids, ammonia, phenol compounds, indole, cresol, and hydrogen sulfide^23^.

The site of fermentation might also be important, as distal but not proximal colonic acetate infusions have been shown to promote fat oxidation and improve metabolic markers in overweight or obese men^24^. SCFAs are absorbed rapidly by the intestinal epithelium, and all three main SCFAs can be utilized as energy sources, contributing to the promotion of adipogenesis and adipocyte differentiation^25,26^, the inhibition of lipolysis, the reduction of circulating fatty acids^25^, and the promotion of fatty acid oxidation^17,27^.

The favorable metabolic changes observed in mice after two weeks of PDX ingestion combined with the WD^17^ stimulated the further investigation of the entire metabolome, in both plasma and adipose tissue. The association between gut microbiota dysbiosis, lipid metabolism, and the development of obesity has been well-established, and microbiota exerts biological functions by modulating lipid compositions, absorption, and digestion, which can affect energy homeostasis^28–30^. Fermentation products can play key roles during this process, through the direct regulation of enterocyte lipid metabolism^31^ or the indirect regulation of the immune system or host metabolism^32,33^.

Bile acids and SCFAs are key components of these processes, acting as signaling molecules^32,33^. In our correlation analyses, *Allobaculum*, in particular, correlated positively with the plasma metabolites of PDX-treated mice, notably with glycine betaine and l-carnitine. *Allobaculum*, *Bifidobacterium*, and *Prevotella*, which are SCFA-producing bacterial genera, were found to decrease in high-caloric diet-fed mice^34^. *Allobaculum*, a butyrate-producing bacterial genus, has been shown to be depleted in obese mice and correlated positively with plasma high-density lipoprotein-cholesterol (HDL-C) levels^35,36^ and negatively with weight gain^37^. *Allobaculum* has been previously shown to be a microbial genera that is sensitive to the changes in the host diet^35^.

Carnitine has been found to have favorable effects on blood lipids, including the reduction of total cholesterol, low-density lipoprotein-cholesterol (LDL-C), and TGs, and to increase HDL-C levels^38^. Carnitine plays an important role in mammalian energy metabolism and the transport of activated long-chain fatty acids from the cytosol to the mitochondrial matrix^39^. The significant increase in carnitine observed in the plasma of mice fed PDX supplementation may be the result of increased endogenous carnitine synthesis or the increased absorption of dietary carnitine.

Endogenously, carnitine is synthesized from the amino acids lysine and methionine, in a sequence of reactions that requires methionine and S-adenosylmethionine (SAM), which acts as a methyl donor^39^. When excess dietary carnitine enters the colon, it is converted into glycine betaine by several bacteria, in the presence of adenosine triphosphate (ATP) or coenzyme A (CoA)^40^. PDX supplementation significantly increased the levels of glycine betaine in the plasma, and a trend (*p* < 0.05) towards increased choline and sarcosine (monomethylglycine) levels was observed in WD-fed mice receiving PDX. These findings indicated altered one-carbon metabolism, as glycine betaine and choline act as methyl donors in the homocysteine-methionine cycle^41,42^. This back-conversion of homocysteine into methionine is important for the conservation of methionine, the detoxification of homocysteine, and the production of SAM, which acts as a methyl donor in many biochemical reactions^43^.

Glycine betaine, choline, and sarcosine can be obtained through the diet. In addition to dietary supplementation, de novo synthesis can be utilized when these molecules are necessary. For instance, choline can be produced via de novo synthesis by bacteria, through the methylation of phosphatidylethanolamine to phosphatidylcholine, and excess choline can be oxidized into betaine^44–46^. Interestingly, PDX supplementation reduced plasma phospholipid levels. In the pathway mapping analysis, unsaturated fatty acid and glycerophospholipid metabolism were significantly altered by the addition of PDX. Because we used the Kyoto Encyclopedia of Genes and Genomes (KEGG) mouse metabolic routes as a reference metabolic dataset, only changes occurring in the mouse and not changes in the microbial metabolism of the intestine were considered. Therefore, the pathway mapping performed in our study cannot produce the full picture of affected metabolic pathways, and methodologies, such as metatranscriptomics of the gut microbiota, should be utilized in future studies to map these changes^47^.

Glycerophospholipid metabolism is associated with the glycine, serine, and threonine metabolism pathway, with choline, betaine, and L-tryptophan as intermediates. Dietary glycine betaine increased hepatic carnitine metabolism and reduced lipid accumulation in the liver^48^. Notably, we observed a significant reduction in the epididymal fat mass in our mice. Thus, increased carnitine might be the result of increased glycine betaine, which reduces the total body fat mass and body fat percentage in humans when given as a supplement^49^. Additionally, in our study, plasma glycine betaine correlated negatively with plasma TG and total cholesterol values, and glycine betaine in adipose tissue was negatively correlated with plasma cholesterol.

The identified FA 18:3 (α-linolenic) decreased in the plasma and increased in adipose tissue with PDX supplementation, and most likely originated from corn oil^50^. Corn oil, together with milk fat, were the two sources of fat in the WD, and milk fat has not been reported to contain α-linolenic acid. Moreover, α-linolenic acid is an essential fatty acid that cannot be synthesized by animals and humans and must be obtained from diet^51^. Therefore, we speculate that the inclusion of PDX might change the absorption of dietary components. The fermentation of carbohydrates lowers the intestinal pH, which could affect the absorption and bioavailability of dietary compounds, as has been observed for calcium^52^. The decreased phospholipid, TG, and cholesterol levels observed in PDX-supplemented mice indicated a change in lipid absorption or metabolism^53^. In vitro transcriptomic analyses of intestinal epithelial Caco-2 cells treated with PDX fermentation products demonstrated an upregulation of fatty acid oxidation and lipid transport^54^.

Indoxyl sulfate was significantly increased in both plasma and fat tissue following PDX treatment, which correlated negatively with blood TGs and total cholesterol in both the plasma and adipose tissue metabolomes. Indoxyl sulfate is produced in the liver from indole, which is a byproduct of tryptophan metabolism by intestinal bacteria^55^. In addition, an isomer of indolecarboxyaldehyde was significantly increased in fat tissue and may be related to the liver metabolism of indole. Interestingly, the tryptophan-derived microbial metabolite indole upregulated the expression of miR-181 in white adipocytes, leading to improvements in body weight gain, glucose tolerance, and insulin sensitivity^56^. This family of micro-RNAs is enriched in white adipose tissue and plays an important role in the regulation and control of lipid metabolism by attenuating the expression of genes involved in lipid synthesis and increasing the expression of genes involved in beta-oxidation^57^. MiR-181 can reduce cellular TG levels in mice and reduce the size of hepatic lipid droplets, and the imbalance of the microbiota—miR-181 axis is vital for obesity and the development of insulin resistance^56–58^.

Bile acids, such as cholic acid, are derived from cholesterol in the liver and are further metabolized by the gut microbiota. The secondary bile acid, deoxycholic acid, was significantly increased in both the plasma and fat tissue by PDX treatment and correlated negatively with both plasma TG and total cholesterol, in both tissue metabolomes^59^. Total plasma cholesterol following PDX treatment correlated inversely with the increase in deoxycholic acid. Therefore, these results suggested that PDX induces changes in the function and composition of the microbiota. Future studies should address these associations in more detail.

A significant decrease in 2,6-di-tert-butylphenol (2,6-DTBP) levels in the fat tissue of PDX-fed mice was also observed; however, the origin or precursor for this molecule is unknown. 2,6-DTBP is utilized as an additive in plastics^60^ and can be found as a contaminant in different food items^61^. As an organic compound, 2,6-DTBP can bioaccumulate in adipose tissue, which may explain why 2,6-DTBP was found in the epididymal adipose tissue of mice. Some microbes, such as *Pseudomonas aeruginosa*^60^, can degrade 2,6-DTBP, and in theory, PDX could increase the population of bacteria that are able to degrade it. The other possibility is that PDX reduced its absorption; however, this remains to be investigated.

In conclusion, our study provided novel insights into the effects of PDX supplementation in WD-fed mice and the resulting modification of the metabolic signatures of plasma and adipose tissue. Further studies remain necessary to investigate whether these changes are involved in the hypolipidemic effects observed with PDX supplementation. Some of the identified molecules with significant changes likely originated from the diet, suggesting that the fermentation of PDX affects the absorption of dietary compounds. The molecules that are involved in one-carbon metabolism, such as glycine betaine and l-carnitine, are essential and beneficial for maintaining cellular homeostasis and have specific roles in lipid metabolism. Two of the metabolites that have been proposed to be from microbial origins, deoxycholic acid and indoxyl sulfate, were increased in both the plasma and adipose tissue metabolomes and may positively affect lipid metabolism. Correlation analysis between metabolites, TG, total cholesterol, and genus level taxa of the cecal microbiota showed significant correlation patterns in both plasma and adipose tissue.

## Materials and methods

### Animal experimentation and sample collection

Animal experiments were conducted in accordance with the guidelines set by the European Community Council Directives (86/609/EEC) and were approved by the Institutional Animal Care and Use Committee of the Provincial Government (Oulu, Finland). All methods were performed following national guidelines. The experimental conditions used for the mouse study, including PDX administration, tissue and blood sampling procedures have been described previously^17^.

In brief, inbred, 10-week-old C57BL/6NCRl male mice (WD group N = 9; WD + PDX group N = 11) were housed in individual cages, with ad libitum food and water, maintained at 21 ± 2 °C, at 40%–60% relative humidity, with 12-h light and dark periods. After 1 week of acclimatization, mice were fed WD formula D12079B (Research Diet Inc., NJ, USA) and orally dosed with either 75 mg PDX (Litesse Ultra, DuPont) in water (referred to as the WD + PDX group) or water alone, as the control (referred to as the WD group), twice daily, for 14 days. The WD formula contained 17% Kcal protein, 43% Kcal carbohydrates, 41% Kcal fat, 5% fiber, and 0.21% cholesterol. The body weights and food intake of all animals were recorded daily. No diarrhea was observed in mice that received PDX during the feeding trial^17^.

After a 12-h fast, terminal blood samples were collected from mice under isoflurane anesthesia, in ethylenediaminetetraacetic acid (EDTA) tubes, and the mice were sacrificed immediately after samples were collected, by cervical dislocation. EDTA-treated blood was centrifuged at 8000 rpm, for 7 min at 4 °C, and plasma was stored at − 80 °C until use. The epididymal fat pad was immersed in RNAlater solution (Thermo Fischer Scientific, Waltham, MA, US) and stored at − 80 °C until use^17^.

### Analysis of total cholesterol, TG, and cecal microbes

Plasma total cholesterol, TG, and cecal microbes as well as the statistical analyses for these parameters were analyzed in our previous study^17^.

### Non-targeted UHPLC-qTOF-MS metabolite profiling analysis

Metabolomic analyses were performed on samples from all 9 control mice and all 10 mice fed with PDX. The non-targeted metabolite profiling pipeline has been described earlier in detail^62^. In brief, an aliquot (100 µL) of each plasma sample was mixed with 400 µL acetonitrile (VWR International, Leuven, Belgium) and mixed by vortex, at maximum speed for 15 s, after which the proteins were precipitated in an ice bath for 15 min, and then, the samples were centrifuged at 16,000×*g* for 10 min. The supernatant was collected and filtered through 0.2-µm polytetrafluoroethylene (PTFE) filters (PALL Corporation). Aliquots of 2 µL were removed from each plasma sample, mixed in one tube, and used as the quality control sample during the analysis.

Frozen fat samples were cryo-ground in 2-ml microcentrifuge tubes, using 4- or 7-mm stainless steel beads, in precooled 2 × 24 adapters that were shaken for 45 s at 30 Hz using a TissueLyser II (Qiagen Finland, Helsinki, Finland). Sample tissue powders were cryo-weighed, and 80% methanol was added (v/v H_2_O, LC–MS Ultra CHROMASOLV®, Fluka), at a ratio of 300 µL solvent/100 mg tissue. The samples were shaken for 20 min, centrifuged for 10 min at 4 °C (13,000 rpm), and the supernatants were filtered using 0.2-µm Acrodisc® Syringe Filters, with a PTFE membrane (PALL Corporation). Aliquots of 2 µL were taken from each tissue sample, mixed in one tube, and used as the quality control sample during the analysis.

The samples were analyzed with an ultra-high-performance liquid chromatography-quadrupole time of flight-mass spectrometry (UHPLC-qTOF-MS) system (Agilent Technologies, Waldbronn, Karlsruhe, Germany), which consisted of a 1290 liquid chromatography (LC) system, a Jetstream electrospray ionization (ESI) source, and a 6540 ultra-high definition (UHD) accurate-mass qTOF spectrometer. Two different chromatographic techniques, reversed-phase (RP) and hydrophilic interaction (HILIC) chromatography, were used for separation, and data were acquired in both positive (+) and negative (−) polarity ionization mode. The injector sample tray was maintained at 4 °C during the analysis. The data acquisition software was MassHunter Acquisition B.04.00 (Agilent Technologies). The quality control samples (separate samples for plasma and tissues) were injected at the beginning of each analysis and every 13th injection. The order of sample analysis was randomized. Details regarding the technical procedures and parameters have been previously described^48^.

### Compound identification

The chromatographic and mass spectrometric characteristics (retention times, exact mass, and MS/MS spectra) of significant differential molecular features were compared with entries in an in-house standard library, publicly available databases, such as METLIN and HMDB, and against published literature. The annotation of each metabolite and the level of identification was determined based on the recommendations published by the Chemical Analysis Working Group (CAWG) Metabolomics Standards Initiative (MSI)^63^: 1 = identified based on a reference standard, 2 = putatively annotated, based on MS/MS spectra or physicochemical properties, and 3 = putatively annotated compared to a compound group (e.g., phosphatidylcholine).

### Statistical analyses

For statistical analyses, *p* values (Welch’s t-test, α level = 0.05) and effect sizes (Cohen’s d) were calculated for comparisons between the study groups, for each molecular feature. Furthermore, a principal component analysis (PCA) was separately performed on the metabolite profiling data from each sample type (fat tissue and plasma), to analyze the overall variance among the mice. We used Bonferroni’s method to explain 95% of the variance in the data and to adjust the α level, to account for multiple testing. Moreover, partial least square discriminant analysis (PLS-DA) was performed, for which the variable importance to projection (VIP) was reported.

Profinder (version B.08.00, Agilent Technologies) was used for feature extraction and peak alignment, the Mass Profiler Professional (version 13, Agilent Technologies) was used for statistical analyses, SIMCA (version 14.0.0, Umetrics) was used to perform multivariate analyses, MS-DIAL (ver.2.52) was used for metabolite identification, and Prism (version 5.03, GraphPad Software Inc.) was used to generate figures. For pathway analyses, MetaboAnalyst version 4.0 was used^64^. For the analysis of metabolites, Welch’s t-test, with *p* < 0.05, was selected.

Spearman’s correlation analyses were conducted to compare genus-level taxa with > 0.1% abundance, fasting plasma TGs, and total cholesterol against metabolite compounds, using the R packages hmisc and gplots^20,21^.

## Supplementary information

Supplementary Information.
