# Supplementary material for: Metabolomics analysis of plasma and adipose tissue samples from mice orally administered with polydextrose and correlations with cecal microbiota
Source: Sci Rep. 2020 Dec 9;10:21577. doi: 10.1038/s41598-020-78484-y (PMC7726573; doi:10.1038/s41598-020-78484-y)
Supplement: Supplementary file 1 — Supplementary Information. [file 41598_2020_78484_MOESM1_ESM.pdf]

Supplementary Information for

Metabolomics analysis of plasma and adipose tissue samples from mice orally administered with polydextrose and correlations with cecal microbiota

Markku Tapani Saarinen*[1], Olli Kärkkäinen[2,3], Kati Hanhineva[2,4], Kirsti Tiihonen[1], Ashley Hibberd[5], Kari Antero Mäkelä[6], Ghulam Shere Raza[6], Karl-Heinz Herzig[6,7] & Heli Anglenius[1]

[1]DuPont Nutrition & Biosciences, Global Health & Nutrition Science, Kantvik, Finland.
[2]Afekta Technologies Ltd., Kuopio, Finland.
[3]School of Pharmacy, University of Eastern Finland, Kuopio, Finland.
[4]Institute of Public Health and Clinical Nutrition, University of Eastern Finland, Kuopio, Finland.
[5]DuPont Nutrition & Biosciences, Genomics & Microbiome Science, St. Louis, MO, USA.
[6]Institute of Biomedicine, Medical Research Center (MRC), University of Oulu, and University Hospital, Oulu, Finland.
[7]Department of Gastroenterology and Metabolism, Poznan University of Medical Sciences, Poznan, Poland.

*Corresponding author: markku.saarinen@dupont.com

Supplementary materials

Supplementary Table 1

Supplementary Figure 1

Supplementary Table 1. All detected molecular features and calculated p-values, Cohen's d values, and VIP values in descending order based on exact mass.

							Fat							Plasma						
							Control		PDX		Welch's t-test		PLS-DA	Control		PDX		Welch's t-test		PLS-DA
Molecular feature	Mass	RT	RP/HILIC	POS/NEG	Compound	ID_level	Mean	SD	Mean	SD	p	d	VIP	Mean	SD	Mean	SD	p	d	VIP
46.0055@10.802001	46.0055	10.80	RP	NEG			17947	2084	20893	4774	0.10087	0.72	1.03	62535	10778	57139	7146	0.24444	-0.58	1.23
60.03192@0.97999966	60.0319	0.98	HILIC	POS	Urea	2	47747	11257	43412	8487	0.38124	-0.43	1.25	184785	18597	182985	27375	0.87037	-0.08	0.42
63.00835@4.416	63.0084	4.42	HILIC	POS			229480	21635	243448	27596	0.24433	0.54	0.81	258000	7889	247257	8875	0.01428	-1.07	1.92
71.07326@4.852998	71.0733	4.85	HILIC	POS	1,2-Diamino-2-methylpropane	2	109327	16011	94194	19635	0.08848	-0.77	1.16	110651	16042	106801	12300	0.58405	-0.27	0.59
72.05762@2.6839986	72.0576	2.68	RP	NEG			65145	23397	61350	18122	0.71140	-0.18	1.45	500203	105672	463579	119025	0.49873	-0.32	0.63
73.05334@5.733998	73.0533	5.73	HILIC	POS			16286	1782	17998	2769	0.13068	0.68	1.01	15811	2233	16127	4368	0.84475	0.09	0.70
74.03674@1.137	74.0367	1.14	HILIC	NEG			164443	35604	171122	24183	0.65740	0.22	0.80	8049	2885	9034	3550	0.52452	0.30	0.65
76.03116@3.9290004	76.0312	3.93	HILIC	POS			6167	336	5843	661	0.19835	-0.57	1.46	22011	1666	21068	1376	0.21671	-0.60	1.05
84.02101@3.0749989	84.0210	3.07	HILIC	POS			46865	14300	47333	24185	0.95992	0.02	1.25	34417	9418	37606	10998	0.51613	0.31	0.61
85.01621@4.514	85.0162	4.51	HILIC	NEG			124289	14079	127801	19502	0.66301	0.20	0.97							
85.05291@0.88999975	85.0529	0.89	HILIC	POS			1871	399	2135	661	0.30973	0.46	0.75	10730	1316	14911	2605	0.00047	1.41	2.34
85.08922@4.01	85.0892	4.01	HILIC	POS			156513	16235	151772	16660	0.55073	-0.28	1.48	349041	50095	345020	42479	0.85880	-0.09	0.47
85.08924@4.2279983	85.0892	4.23	HILIC	POS			116343	19048	115730	15898	0.94275	-0.04	1.19	347992	38141	357284	32215	0.59019	0.26	0.65
89.04738@5.5339975	89.0474	5.53	HILIC	POS			43898	9261	40750	9371	0.48558	-0.33	1.63	24817	2820	24707	1721	0.92472	-0.05	0.50
89.04771@5.5959973	89.0477	5.60	HILIC	POS	Sarcosine	2	63773	9648	68590	6917	0.25422	0.56	0.91	49073	8324	58260	6605	0.02236	1.05	1.72
89.04773@5.522002	89.0477	5.52	HILIC	NEG			66161	24751	66444	38530	0.98514	0.01	1.26	19673	2106	17343	3138	0.07707	-0.79	1.35
89.96927@4.416	89.9693	4.42	HILIC	POS			135505	38804	169945	60044	0.15948	0.63	0.81	422262	17294	426365	19792	0.64479	0.22	0.67
90.03155@5.141999	90.0316	5.14	HILIC	NEG			873712	139546	914505	188475	0.60451	0.24	0.83	6608070	1385686	7114725	1810018	0.50984	0.31	0.76
90.03163@1.3880004	90.0316	1.39	HILIC	NEG			108885	20791	116819	26818	0.48866	0.32	1.09	63958	16456	70059	8883	0.36420	0.46	1.04
90.03165@4.4380007	90.0317	4.44	HILIC	NEG			254590	40257	280651	65351	0.31358	0.46	1.06							
90.03167@5.4080014	90.0317	5.41	HILIC	NEG			13433	1708	12911	2213	0.57867	-0.26	0.96	61243	12448	63802	16492	0.71196	0.17	0.67
90.03195@1.5289996	90.0320	1.53	HILIC	NEG			220691	75619	269906	122678	0.31087	0.46	0.97	105012	26202	122857	20763	0.13686	0.71	1.30
94.0528@5.5310016	94.0528	5.53	HILIC	POS			77745	25952	95399	18696	0.12798	0.74	1.08	3130	1382	4448	2323	0.15359	0.64	1.10
97.96723@7.701001	97.9672	7.70	HILIC	NEG			643254	566209	966749	669141	0.28142	0.50	1.40	325796	64895	323231	169030	0.96547	-0.02	0.38
100.01611@1.1240003	100.0161	1.12	HILIC	NEG			53326	7509	57600	7270	0.24033	0.56	0.81	2587	1639	9481	2923	0.00001	1.64	2.78
101.04702@5.7400017	101.0470	5.74	HILIC	POS			22381	3270	24588	2538	0.13807	0.71	0.91	19176	2549	19440	5265	0.89067	0.06	0.60
101.04733@6.3020015	101.0473	6.30	HILIC	POS			28720	5917	27912	4451	0.75322	-0.16	1.08	668	347	942	685	0.28914	0.48	1.01
101.08379@4.4980006	101.0838	4.50	HILIC	POS			56929	14426	57181	15586	0.97206	0.02	1.34							
101.08392@4.858001	101.0839	4.86	HILIC	POS			44300	10811	44819	11898	0.92388	0.05	1.36	8236	2268	12123	2755	0.00414	1.22	2.00
101.93991@12.438002	101.9399	12.44	RP	NEG										99142	15969	107534	20859	0.34663	0.44	0.91
102.04661@3.939	102.0466	3.94	HILIC	POS			11292	1291	10736	998	0.33266	-0.47	1.08	46515	2560	45140	2795	0.29167	-0.50	0.91
103.06319@4.7200007	103.0632	4.72	HILIC	POS			82689	26896	75885	29495	0.61580	-0.24	0.66	36386	5060	50651	15223	0.01602	1.04	1.74
103.06335@5.065999	103.0634	5.07	HILIC	POS			115216	26901	108058	36425	0.63765	-0.22	1.35	6604	1873	6366	1317	0.76512	-0.15	0.33
103.09962@1.5369995	103.0996	1.54	HILIC	POS	Choline	2	22412681	8182736	25336070	9117111	0.48345	0.33	1.12	4100030	582656	5181473	839461	0.00485	1.19	1.93
103.09988@6.1620016	103.0999	6.16	HILIC	POS			244516	59996	214807	68752	0.34110	-0.45	1.68	16631	8169	18165	9086	0.71100	0.18	0.55
105.04269@6.11	105.0427	6.11	HILIC	POS			48903	4866	51647	5695	0.28524	0.50	0.73	28074	4520	30643	4854	0.26171	0.53	0.97
105.07897@3.5719998	105.0790	3.57	HILIC	POS	Diethanolamine	2	13986	2579	14118	3890	0.93233	0.04	0.74	58970	17913	60697	15832	0.83331	0.10	0.27
106.02449@0.9810001	106.0245	0.98	HILIC	NEG			21404	15746	13417	14345	0.28211	-0.51	0.60	19210	34010	13801	40358	0.76125	-0.14	0.52
106.04169@9.554996	106.0417	9.55	RP	POS			209520	7495	208757	7587	0.83353	-0.10	0.81	220187	6630	218922	6959	0.69861	-0.18	0.55
108.06886@4.605	108.0689	4.61	HILIC	POS			23637	3937	27749	6273	0.10781	0.72	1.02	5168	1865	8786	5585	0.07782	0.77	1.27
108.09394@10.0460005	108.0939	10.05	RP	POS			41037	32179	40887	27232	0.99175	-0.01	0.65							
109.00141@7.3200045	109.0014	7.32	HILIC	POS			26938	3423	25424	3815	0.38753	-0.41	1.26	13425	1804	14495	3565	0.42132	0.36	0.63
109.01969@5.6040025	109.0197	5.60	HILIC	POS			57801	47062	82697	52243	0.30256	0.48	0.94	386	263	938	500	0.01286	1.12	1.79
109.06396@6.7230034	109.0640	6.72	HILIC	POS			70030	14907	68920	14548	0.87578	-0.08	0.73	18240	2784	19372	4601	0.52822	0.29	0.61
110.10952@10.475999	110.1095	10.48	RP	POS										58196	5882	56827	5746	0.62636	-0.23	0.76
110.10968@9.658998	110.1097	9.66	RP	POS			39452	5657	41302	8512	0.58832	0.25	1.14	58890	12926	53504	16525	0.44797	-0.35	0.66
111.04306@3.5609994	111.0431	3.56	HILIC	POS	Isocytosine	2	96563	28966	111818	54903	0.46031	0.33	1.19	125890	15305	128458	24843	0.79100	0.12	0.45
111.04319@2.2030003	111.0432	2.20	HILIC	POS	Cytosine	2	65310	12637	62606	13773	0.67003	-0.20	1.53	402112	60814	388614	32325	0.58159	-0.28	0.68
111.04324@3.5609994	111.0432	3.56	HILIC	POS	Isocytosine	2	95381	28624	111798	54902	0.42618	0.36	1.17	125932	15318	128450	24843	0.79503	0.12	0.45
111.07927@5.5429997	111.0793	5.54	HILIC	POS			522491	172514	644753	141693	0.12653	0.73	1.08	15430	7114	29417	18523	0.04632	0.87	1.42
112.06339@6.306	112.0634	6.31	HILIC	POS			17151	2762	17225	3111	0.95794	0.03	1.21	10250	2145	10899	3841	0.65638	0.20	0.52
113.05862@1.2799996	113.0586	1.28	HILIC	POS	Creatinine	2	442086	88496	429161	92716	0.76664	-0.14	1.31	260037	29008	266756	50818	0.72899	0.16	0.58
113.99288@4.386001	113.9929	4.39	HILIC	NEG			292496	87362	353866	174293	0.34642	0.42	0.69	1250016	137747	1270289	157697	0.77452	0.14	0.58
115.06328@4.8690023	115.0633	4.87	HILIC	POS	Proline	2	1052130	189201	1025269	211761	0.77985	-0.13	0.70	735856	88125	819737	90025	0.06294	0.85	1.65
115.10005@1.9339988	115.1001	1.93	HILIC	POS			12744	3840	13625	3672	0.62836	0.23	1.08	44849	10272	47904	9091	0.51892	0.31	0.93
116.0113@3.4140007	116.0113	3.41	HILIC	NEG			211211	39201	230772	45585	0.34100	0.45	0.72							
116.0474@1.0530001	116.0474	1.05	HILIC	NEG			7324	2342	5241	1569	0.04960	-0.94	1.64	157293	32865	135534	44947	0.25145	-0.53	0.85
116.04746@1.8769996	116.0475	1.88	RP	NEG			4491	1546	3358	814	0.08649	-0.85	1.31	79435	16077	80389	27755	0.92845	0.04	0.56

117.04236@2.8580012	117.0424	2.86	HILIC	NEG	N-Acetylglycine	2	27800	12086	24831	9510	0.57830	-0.27	1.38	114226	30232	100860	46297	0.47079	-0.33	0.58
117.05377@5.6649976	117.0538	5.66	HILIC	POS			157029	29836	132267	15814	0.05633	-0.94	1.87	25409	5937	23589	3886	0.46740	-0.36	1.01
117.05798@4.038	117.0580	4.04	HILIC	POS			3441	559	3087	467	0.17036	-0.66	1.78	25351	3654	25897	3147	0.74205	0.16	1.19
117.07895@4.8509984	117.0790	4.85	HILIC	POS	Valine	2	161518	19256	144102	22255	0.09229	-0.77	1.01	403311	55025	391777	40379	0.62768	-0.24	0.57
117.07897@3.8430011	117.0790	3.84	HILIC	POS	Glycine betaine	1	10290322	3751651	13612562	6085106	0.17329	0.61	1.00	9143521	1100648	16563172	3890727	0.00009	1.56	2.55
118.02664@1.1349996	118.0266	1.13	HILIC	NEG	Succinic acid	2	1017968	215622	1076904	176654	0.54205	0.30	0.73	50185	20378	62871	24259	0.24369	0.54	1.00
118.02683@1.1800001	118.0268	1.18	RP	NEG			210490	52760	210347	62252	0.99585	0.00	0.70							
118.06304@0.9820002	118.0630	0.98	HILIC	NEG	DL-2-Hydroxyvaleric acid	2								13665	6854	11347	3940	0.41144	-0.42	0.77
119.05763@5.735	119.0576	5.74	HILIC	POS	Threonine	2	155527	15296	172036	23919	0.09349	0.75	0.98	143064	18389	146486	38949	0.80925	0.11	0.65
119.05821@5.7239995	119.0582	5.72	HILIC	NEG	Threonine	2	68713	9047	73022	10222	0.35607	0.43	1.10	74793	11062	81165	17389	0.35814	0.42	0.76
119.07367@1.9360001	119.0737	1.94	RP	POS			315806	56957	322099	62242	0.82573	0.10	1.32	505803	42535	550347	48850	0.05362	0.87	1.55
119.07401@3.9259999	119.0740	3.93	HILIC	POS	2-Amino-1-phenylethanol	2	87754	5243	81586	6611	0.04051	-0.91	1.44	414893	21317	401597	27929	0.26717	-0.51	0.94
119.07405@1.278	119.0741	1.28	RP	POS										627523	40463	661322	54977	0.15075	0.65	1.20
119.0836@3.8600001	119.0836	3.86	HILIC	POS			42416	15771	57788	25678	0.13710	0.67	1.00	37453	5100	65356	14580	0.00008	1.56	2.54
119.09417@1.9869998	119.0942	1.99	HILIC	POS			200825	169887	520275	599338	0.13417	0.66	0.80	17743	13235	22589	15959	0.49046	0.32	0.68
120.09387@8.645997	120.0939	8.65	RP	POS			38831	4123	38281	4999	0.80111	-0.12	0.53	34794	25466	40177	12747	0.59596	0.27	0.44
121.07391@5.4550014	121.0739	5.46	HILIC	POS			72062	9374	63393	7960	0.05397	-0.90	2.14	93897	7351	94833	7497	0.79331	0.13	0.55
121.91705@3.1689992	121.9171	3.17	HILIC	POS			86707	9010	85611	12513	0.83173	-0.10	1.39	57542	3644	64113	6002	0.01081	1.09	1.81
123.99282@6.166	123.9928	6.17	HILIC	POS			118520	35712	101083	39682	0.34048	-0.45	1.71	4089	3389	4829	3350	0.64965	0.22	0.63
124.97888@7.8630037	124.9789	7.86	HILIC	POS			68234	2892	70140	2119	0.14083	0.71	1.88	86677	4123	86949	11322	0.94499	0.03	0.34
125.01474@5.348003	125.0147	5.35	HILIC	POS	Taurine	2	587563	57958	646040	95050	0.12647	0.68	1.06	57786	3354	63173	12027	0.20140	0.56	0.94
125.01494@5.330998	125.0149	5.33	HILIC	NEG	Taurine	2	2943020	97080	3030230	147540	0.15010	0.65	0.89	671668	69511	711109	162006	0.49880	0.30	0.78
125.05871@1.8619998	125.0587	1.86	HILIC	POS			4633	908	5329	1153	0.16928	0.63	1.18	40215	5403	39423	2704	0.71216	-0.19	0.47
125.09522@4.605	125.0952	4.61	HILIC	POS			132510	24878	171684	45374	0.03330	0.93	1.23	23369	9969	46981	35459	0.06774	0.80	1.32
125.99864@3.5260005	125.9986	3.53	HILIC	NEG			166328	31314	155296	34218	0.48539	-0.33	1.05	852651	174787	479276	191170	0.00043	-1.43	2.44
127.01145@5.3289986	127.0115	5.33	HILIC	NEG			105282	3820	107371	6553	0.41044	0.37	0.85	27871	3086	28786	6439	0.69762	0.18	0.77
128.05861@5.6589975	128.0586	5.66	HILIC	POS			19205	3318	24576	3286	0.00320	1.26	2.17	13846	2597	20099	6394	0.01388	1.05	1.71
128.05902@6.050002	128.0590	6.05	HILIC	NEG			48413	9405	55569	14153	0.21609	0.56	0.96	29742	4758	34589	9118	0.16617	0.62	1.05
129.0425@4.2709975	129.0425	4.27	HILIC	NEG	D-Pyroglutamic acid	2	37482	10460	54337	24588	0.07032	0.79	0.93	63838	21094	98698	55088	0.08802	0.75	1.27
129.04262@6.300001	129.0426	6.30	HILIC	POS			50550	10918	51498	7404	0.83672	0.10	1.07							
129.04266@6.292	129.0427	6.29	HILIC	NEG			37398	10564	37166	9243	0.96148	-0.02	1.21							
129.04272@6.063998	129.0427	6.06	HILIC	POS	Glutamine fragment	3	406433	46492	438921	65626	0.23579	0.54	0.97	294870	24947	327359	40822	0.05325	0.85	1.38
129.07901@4.144	129.0790	4.14	HILIC	POS										30826	6434	66519	20881	0.00024	1.49	2.48
129.07922@4.9700007	129.0792	4.97	HILIC	POS	L-Pipecolic acid	2	72653	18870	58984	12359	0.10020	-0.80	1.92	53350	8319	48149	3665	0.12959	-0.77	1.26
129.07922@1.5690001	129.0792	1.57	HILIC	POS										57352	26357	49778	16849	0.49292	-0.34	0.69
129.0793@1.7019999	129.0793	1.70	HILIC	POS										54496	24134	46779	16246	0.45184	-0.38	0.78
129.96693@3.136001	129.9669	3.14	HILIC	NEG			370042	36156	381358	53047	0.59832	0.24	1.04	249153	16703	271290	33190	0.08540	0.76	1.25
130.01608@1.132	130.0161	1.13	HILIC	NEG			69781	14249	73258	11712	0.58620	0.27	0.82							
130.01828@0.97999966	130.0183	0.98	HILIC	NEG			30880	15591	34878	13330	0.57261	0.28	0.82	96271	52856	52808	50177	0.09444	-0.78	1.37
130.02652@2.4420002	130.0265	2.44	HILIC	NEG										159707	83851	235759	132630	0.15683	0.64	1.03
130.06264@0.979	130.0626	0.98	HILIC	NEG			2475	1311	2016	908	0.41396	-0.41	1.17	257310	68360	193214	71337	0.06890	-0.83	1.34
130.06306@3.184999	130.0631	3.18	RP	NEG			8756	1373	6835	1233	0.00698	-1.19	2.22	603805	74313	626240	202479	0.75091	0.14	0.59
130.06306@3.4290004	130.0631	3.43	RP	NEG			14412	2943	11363	1897	0.02466	-1.07	2.14	543001	86027	562870	201037	0.78207	0.12	0.59
130.06313@0.879	130.0631	0.88	HILIC	NEG			15559	2794	12126	2991	0.02212	-1.02	2.32	736880	94303	694649	159213	0.49397	-0.31	0.58
131.06934@5.522002	131.0693	5.52	HILIC	NEG			97376	40807	97468	64823	0.99713	0.00	1.22	25738	2916	22324	3591	0.03901	-0.92	1.53
131.06938@5.5350027	131.0694	5.54	HILIC	POS	Creatine	2	5348625	1338959	4926908	1686292	0.56160	-0.27	1.56	2647196	284143	2445043	196196	0.10945	-0.78	1.27
131.07356@4.0630007	131.0736	4.06	HILIC	POS										30795	5031	29530	3307	0.55033	-0.30	0.96
131.09456@4.015	131.0946	4.02	HILIC	POS	Leucine	2	867459	100411	827775	87935	0.39188	-0.41	1.56	1953213	264428	1964952	249396	0.92468	0.05	0.37
131.0948@4.233001	131.0948	4.23	HILIC	POS	Isoleucine	2	583835	90592	578295	75044	0.89127	-0.07	1.29	1746149	194383	1787430	167313	0.64060	0.23	0.59
131.09502@1.347	131.0950	1.35	RP	POS	Isoleucine	1	2846667	648492	2792975	677897	0.86615	-0.08	1.33	2321442	416316	2376842	342086	0.76573	0.15	0.49
131.9516@10.474	131.9516	10.47	RP	NEG			140051	15339	153088	17975	0.11442	0.72	0.84	135132	10284	129381	17994	0.40668	-0.38	0.76
132.05328@6.1609983	132.0533	6.16	HILIC	NEG			81509	15086	80291	20585	0.88652	-0.07	1.52	16806	3265	15904	3541	0.58189	-0.26	0.70
132.05354@6.1900015	132.0535	6.19	HILIC	POS	L-Asparagine	2	15955	3317	17187	5041	0.54186	0.28	0.96	41283	8294	39999	7783	0.74144	-0.16	0.55
132.07875@4.066	132.0788	4.07	RP	NEG			22943	10733	22139	9554	0.87031	-0.08	1.53	166778	36967	155353	50293	0.58578	-0.25	0.55
132.95857@12.438002	132.9586	12.44	RP	NEG										100190	12905	94519	11915	0.35168	-0.45	0.86
133.02382@0.979	133.0238	0.98	HILIC	NEG			13413	3308	10571	3525	0.09601	-0.77	0.91	51286	11046	43173	11022	0.13946	-0.69	1.22
133.03738@6.6950006	133.0374	6.70	HILIC	POS			96978	19182	108572	25538	0.28610	0.49	0.72							
133.0377@6.686998	133.0377	6.69	HILIC	NEG	Aspartic acid	2	151847	20649	137976	26650	0.22946	-0.55	0.81	1520	241	1987	855	0.12569	0.67	1.22
133.0638@4.932999	133.0638	4.93	RP	POS			150696	55779	179967	90816	0.41249	0.37	0.97	668251	409067	672772	422090	0.98193	0.01	0.69
134.02176@5.317001	134.0218	5.32	HILIC	NEG			122180	24897	151378	65333	0.21546	0.55	1.05	26058	8654	30358	13774	0.43022	0.36	0.60
134.10963@8.7890005	134.1096	8.79	RP	POS			41380	13648	42765	12614	0.82758	0.11	0.64	39458	29563	42222	19753	0.82392	0.11	0.21
134.10982@8.553	134.1098	8.55	RP	POS			146663	15388	148361	15798	0.82087	0.11	0.86	135236	99454	158677	56207	0.56293	0.29	0.48
134.10986@8.645997	134.1099	8.65	RP	POS			180546	19765	173867	26612	0.54917	-0.28	0.55	166501	124516	197072	67322	0.54389	0.31	0.50

135.05437@6.5360017	135.0544	6.54	HILIC	POS			27798	6898	30108	8694	0.53739	0.29	0.84							
135.05446@1.1650002	135.0545	1.17	HILIC	POS			145465	43853	140435	53009	0.82815	-0.10	1.53							
135.06882@5.0800014	135.0688	5.08	HILIC	POS			23227	4512	23215	4036	0.99561	0.00	0.71	29888	7148	34194	5496	0.18102	0.65	1.25
136.03705@6.6360006	136.0371	6.64	HILIC	NEG			70353	23895	101262	46471	0.08748	0.75	1.02							
136.03714@5.5330014	136.0371	5.53	HILIC	NEG			229401	50561	290770	119626	0.16441	0.62	1.02	128585	67540	138636	78567	0.77389	0.14	0.56
136.03831@2.57	136.0383	2.57	HILIC	POS			1674717	467187	1714600	627508	0.87897	0.07	1.33							
136.03842@1.4279997	136.0384	1.43	HILIC	NEG	Threonic acid	2	161735	50812	172963	55640	0.66066	0.21	1.13							
136.03847@1.4379998	136.0385	1.44	HILIC	POS			1018631	150910	1079964	180743	0.44301	0.36	0.79							
136.03854@1.479	136.0385	1.48	RP	POS	Hypoxanthine	1	1298136	351580	1328059	631119	0.90029	0.06	1.29							
136.03864@2.5639997	136.0386	2.56	HILIC	NEG			75473	22729	78610	39716	0.83592	0.09	1.28							
136.0637@2.2770002	136.0637	2.28	HILIC	POS	2-Aminobenzamide	2	405816	64330	470070	90102	0.09517	0.75	0.91	59927	19311	69034	16973	0.30989	0.49	0.89
137.0459@4.8739977	137.0459	4.87	HILIC	POS			9433	1700	10319	2776	0.41680	0.37	1.12	19601	3884	21548	4009	0.31166	0.48	1.10
138.04295@1.0929998	138.0430	1.09	HILIC	POS			52359	28930	55919	27946	0.79547	0.13	0.81	21448	15716	22955	13993	0.83464	0.10	0.37
139.06091@3.8430011	139.0609	3.84	HILIC	POS			58501	29095	70602	33666	0.42441	0.38	0.95	30608	4102	61979	16257	0.00009	1.58	2.56
143.07324@4.038	143.0732	4.04	HILIC	POS			4184	756	3897	869	0.46450	-0.34	1.60	33121	4105	32957	4013	0.93312	-0.04	1.19
143.09451@3.0729985	143.0945	3.07	HILIC	POS			42162	13785	42644	21767	0.95511	0.03	1.22	32619	9504	33221	8719	0.89151	0.07	0.29
144.00404@2.9900007	144.0040	2.99	HILIC	NEG			30863	10222	37982	12673	0.20361	0.59	0.79							
144.00517@1.535	144.0052	1.54	HILIC	NEG			128959	36874	152701	49886	0.26126	0.52	0.91							
144.0073@1.316	144.0073	1.32	HILIC	NEG			77144	10470	85789	17049	0.20377	0.57	0.82							
144.0421@3.093001	144.0421	3.09	HILIC	POS			59061	17772	59495	31375	0.97096	0.02	1.28	44032	12619	48758	13377	0.45235	0.36	0.68
145.05247@4.035002	145.0525	4.04	HILIC	POS			7350	1221	7052	889	0.57202	-0.28	1.49	55077	6499	54447	6880	0.84445	-0.09	1.08
145.07338@1.2159997	145.0734	1.22	HILIC	NEG			5745	2351	5054	1880	0.50933	-0.32	1.54	40736	8828	32655	9218	0.07523	-0.82	1.33
145.07407@5.36	145.0741	5.36	HILIC	POS			12559	3059	12099	3477	0.76893	-0.14	1.56							
145.08472@3.8949997	145.0847	3.89	HILIC	POS	isomer of indolecarboxaldehyde	3	36349	28634	120299	56706	0.00094	1.35	2.15	7414	8465	7357	7644	0.98841	-0.01	0.57
145.11037@3.4730003	145.1104	3.47	HILIC	POS	Acetylcholine cation	2	1904720	222062	1521644	340371	0.01034	-1.10	2.23	139251	39230	135136	26149	0.80276	-0.13	0.39
145.15762@7.8429985	145.1576	7.84	HILIC	POS			160599	69341	147676	57937	0.67864	-0.20	0.76	5677	2609	8268	4878	0.16880	0.61	1.03
145.15785@7.670001	145.1579	7.67	HILIC	POS			40994	17336	28316	12300	0.10271	-0.79	1.46	1600	737	2259	1619	0.27564	0.48	0.89
146.06908@5.9069986	146.0691	5.91	HILIC	POS	Glutamine isomer	3	43236	25345	149422	41666	0.00000	1.67	2.32	2277	1218	7714	3216	0.00026	1.47	2.42
146.06914@6.2020016	146.0691	6.20	HILIC	POS			18544	5322	22892	5771	0.11482	0.73	0.92							
146.0695@6.050002	146.0695	6.05	HILIC	NEG	Glutamine	1	852706	180715	976325	259182	0.24996	0.53	0.98	523978	87741	609984	167661	0.18091	0.60	1.00
146.06958@6.0650015	146.0696	6.07	HILIC	POS	Glutamine	1	1609274	190629	1731563	269082	0.27492	0.50	0.99	1162913	111448	1288410	167371	0.07381	0.80	1.28
146.10616@7.040999	146.1062	7.04	HILIC	POS			69206	27690	85469	64966	0.48648	0.31	1.12	22130	4330	26772	17145	0.42661	0.35	0.63
146.99657@5.3439984	146.9966	5.34	HILIC	POS			99527	6728	104438	10621	0.24902	0.52	0.88	6745	730	7114	974	0.37142	0.41	0.70
147.03546@1.9550003	147.0355	1.96	HILIC	POS			303761	58516	341357	65517	0.21559	0.58	0.91							
147.05322@6.4310007	147.0532	6.43	HILIC	POS	Glutamate isomer	3	20076	17127	72262	24717	0.00005	1.54	2.04							
147.05325@6.3029976	147.0533	6.30	HILIC	POS			504954	111917	503636	70620	0.97730	-0.01	1.08	11251	7084	13820	7659	0.47060	0.34	0.79
147.05339@6.2929997	147.0534	6.29	HILIC	NEG	Glutamate	2	731147	193597	755768	197222	0.79347	0.13	1.12	8510	4284	8584	2742	0.96675	0.02	0.77
148.01599@10.925997	148.0160	10.93	RP	POS			58406	23975	47476	4386	0.23714	-0.63	0.85	38167	7925	40910	9490	0.51262	0.31	0.77
148.05183@9.554996	148.0518	9.55	RP	POS			71212	3467	69836	2800	0.37698	-0.43	0.88	70932	3487	69644	3795	0.46371	-0.35	0.62
149.05087@0.88999975	149.0509	0.89	HILIC	POS			5735	1178	7509	1862	0.02481	0.98	1.06	34832	5316	45621	8679	0.00480	1.18	1.98
149.05089@4.5950007	149.0509	4.60	HILIC	POS	Methionine	2	47369	18131	37796	15681	0.25489	-0.55	0.97	56723	10820	53427	17173	0.62599	-0.22	0.56
151.06296@4.0630007	151.0630	4.06	HILIC	POS			56139	35894	57484	59710	0.95359	0.03	1.12							
152.0341@1.7620003	152.0341	1.76	HILIC	NEG	Xanthine	2	824685	147272	889657	160260	0.38324	0.41	1.06	4713	1671	7003	3736	0.10409	0.72	1.18
152.05824@0.7789997	152.0582	0.78	HILIC	POS	1,6-dihydro-1-methyl-6-oxo-3-Pyridinecarboxamide	2	32356	14350	26912	6722	0.34398	-0.49	1.43	174540	31544	159206	46976	0.42020	-0.37	0.85
152.12039@8.6449995	152.1204	8.64	RP	POS			155665	15888	151444	21308	0.63622	-0.22	0.51	142549	105448	168239	56523	0.54636	0.31	0.50
153.00945@3.5309994	153.0095	3.53	HILIC	NEG			10905	3516	12387	3305	0.37444	0.43	0.61	186630	124303	212987	105588	0.63956	0.23	0.52
153.0471@5.5370016	153.0471	5.54	HILIC	POS			75559	12777	71158	11023	0.45180	-0.37	1.50	53057	4080	50939	8033	0.47931	-0.32	0.63
153.0512@5.5370016	153.0512	5.54	HILIC	POS			76977	11951	71795	9902	0.33958	-0.46	1.54	54511	4474	52321	8422	0.48993	-0.31	0.64
154.0376@3.6510005	154.0376	3.65	HILIC	NEG			4778	603	5364	1531	0.28733	0.47	1.00	55176	8515	58504	10070	0.45748	0.35	0.67
154.09943@10.0460005	154.0994	10.05	RP	POS			58002	48207	55550	42054	0.91111	-0.05	0.66							
155.03471@3.834	155.0347	3.83	HILIC	POS			38082	22188	50436	34371	0.36944	0.41	1.08	8483	1562	19395	6385	0.00025	1.50	2.43
155.06956@6.718001	155.0696	6.72	HILIC	POS	Histidine	2	518609	109737	503050	114099	0.77249	-0.14	0.74	134740	24522	147118	40372	0.43365	0.36	0.68
155.06961@6.7019987	155.0696	6.70	HILIC	NEG	Histamine	2	14411	3858	14227	3929	0.92151	-0.05	0.68	49566	7469	51306	8978	0.65894	0.21	0.56
157.0736@0.97599995	157.0736	0.98	HILIC	NEG										15888	6087	10007	3479	0.03142	-1.04	1.69
157.14706@7.380002	157.1471	7.38	RP	POS			970479	44193	977095	50590	0.77072	0.14	0.94	1061160	23764	1074865	31628	0.30800	0.47	1.10
157.95676@4.425999	157.9568	4.43	HILIC	POS			55292	13802	69066	23939	0.14515	0.65	0.81	192684	5866	195103	9663	0.52142	0.29	0.57
158.02023@2.5709996	158.0202	2.57	HILIC	POS			55284	8451	48883	7953	0.11996	-0.73	1.92							
158.02054@1.448	158.0205	1.45	HILIC	POS			136054	31358	123428	32731	0.41643	-0.39	0.71							
158.04393@1.9479988	158.0439	1.95	HILIC	NEG			272915	37156	278120	47325	0.79678	0.12	0.99	1254229	136166	1282413	170574	0.70124	0.18	0.74
158.04416@1.9719999	158.0442	1.97	HILIC	POS										18701	2166	19910	4686	0.47991	0.32	0.68
158.06921@6.307003	158.0692	6.31	HILIC	POS			109170	15544	110201	20170	0.90381	0.06	1.26	69130	16269	74389	22829	0.57602	0.26	0.61
158.08437@4.0370016	158.0844	4.04	HILIC	POS			7357	1139	6910	1075	0.40883	-0.40	1.45	52842	7213	52577	5509	0.93258	-0.04	1.15
159.06812@1.6609997	159.0681	1.66	HILIC	POS	1-Acetylindole (MS-DIAL, serotonin fragment?)	2	46091	11609	51107	16913	0.46671	0.33	0.83	243665	93612	840668	742811	0.02975	0.95	1.57

159.0896@0.972	159.0896	0.97	HILIC	NEG			4980	2459	3805	1797	0.27658	-0.53	1.80	67898	28241	40523	12916	0.02819	-1.09	1.77
159.12607@2.4410005	159.1261	2.44	HILIC	POS										61843	7197	69793	8644	0.04735	0.89	1.50
159.1262@2.1650007	159.1262	2.17	HILIC	POS	5-AVAB	1	1978334	604070	2460374	1503219	0.37093	0.40	0.93	824196	243251	1039388	316017	0.12004	0.71	1.15
159.9862@7.3159995	159.9862	7.32	HILIC	NEG			61819	7178	57765	7105	0.24809	-0.55	1.13	33288	4124	35261	10503	0.59542	0.24	0.55
161.05067@1.3880002	161.0507	1.39	HILIC	POS			37358	2839	36143	3767	0.44550	-0.35	0.93							
161.0684@6.028	161.0684	6.03	HILIC	NEG			22784	5658	24367	6541	0.58916	0.25	0.97	1380	295	1590	706	0.40742	0.37	0.68
161.06848@6.0240016	161.0685	6.02	HILIC	POS			29793	6326	29483	3000	0.90067	-0.06	0.88	2864	906	2598	1149	0.58944	-0.25	0.56
161.0689@3.6580002	161.0689	3.66	HILIC	NEG										51356	20674	37705	19883	0.17504	-0.64	1.11
161.1051@4.5080004	161.1051	4.51	HILIC	POS			12817227	3293019	13014612	3969471	0.90949	0.05	1.36							
161.10529@4.8509984	161.1053	4.85	HILIC	POS	L-Carnitine	1	10644147	2726544	10925474	3327653	0.84593	0.09	1.33	1731306	520815	2831511	612082	0.00065	1.39	2.27
162.12578@5.4880023	162.1258	5.49	RP	POS			71880	8971	78035	8719	0.16134	0.66	1.08	87066	12812	91442	26439	0.65197	0.20	0.40
164.04788@5.0820003	164.0479	5.08	HILIC	POS			24767	4209	24749	5260	0.99371	0.00	0.55	32133	6026	36763	6595	0.13819	0.69	1.42
165.05566@6.166	165.0557	6.17	HILIC	POS			47624	14604	40516	15347	0.32955	-0.46	1.68	2001	1548	1882	1449	0.86886	-0.08	0.67
165.0789@1.278	165.0789	1.28	RP	NEG										226393	15236	246402	24829	0.05088	0.86	1.55
165.0789@1.9369996	165.0789	1.94	RP	NEG			111316	19059	110660	23131	0.94813	-0.03	1.36	163879	13269	176092	18275	0.11834	0.71	1.31
165.07898@3.9290004	165.0790	3.93	HILIC	POS	Phenylalanine	2	328570	20643	310129	21201	0.07982	-0.81	1.74	1554292	91485	1504218	102998	0.28987	-0.50	0.93
165.07942@1.9360001	165.0794	1.94	RP	POS	Phenylalanine	1	1100045	209951	1126371	220075	0.79887	0.12	1.29	1832309	181384	1979704	194648	0.11501	0.73	1.35
165.07942@1.2819998	165.0794	1.28	RP	POS										2327162	164685	2476908	226743	0.12251	0.70	1.28
167.02557@3.2119987	167.0256	3.21	HILIC	NEG			29040	5101	28452	7460	0.84532	-0.09	1.42	40737	8780	28992	5751	0.00605	-1.26	2.20
167.98222@6.7279983	167.9822	6.73	HILIC	NEG	Phospho(enol)pyruvic acid	2	34526	22074	89568	109975	0.15079	0.63	0.86							
168.02838@4.737	168.0284	4.74	HILIC	NEG			429069	97123	451229	63189	0.58638	0.27	0.70	64400	14668	71656	20968	0.40021	0.39	0.71
168.05128@6.063998	168.0513	6.06	HILIC	POS			103570	22068	108409	22498	0.65243	0.22	0.96	50898	13050	57364	19948	0.41930	0.37	0.68
169.08504@6.2769966	169.0850	6.28	HILIC	POS	1-methylhistidine	2	151745	34159	162964	60086	0.62491	0.22	1.13	212499	97478	247827	169552	0.58684	0.25	0.64
169.08507@6.813998	169.0851	6.81	HILIC	POS			34401	11416	44776	13923	0.09949	0.75	0.97	10265	2102	12637	3359	0.08479	0.77	1.27
171.12605@10.0460005	171.1261	10.05	RP	POS			395285	335794	378501	287421	0.91204	-0.05	0.66							
171.1265@9.951002	171.1265	9.95	RP	POS			135405	127977	80611	114929	0.35893	-0.44	0.87							
171.1268@4.432999	171.1268	4.43	RP	POS			325468	208777	485421	218393	0.13153	0.70	0.81	31520	12181	33685	13679	0.72705	0.17	0.65
172.0137@6.4849997	172.0137	6.48	HILIC	NEG	Glycerophosphate	2	78819	77493	209354	218583	0.10270	0.72	0.96							
173.00142@5.531999	173.0014	5.53	HILIC	POS			29162	9089	34215	5230	0.18717	0.66	1.19	990	646	2345	1493	0.03840	1.01	1.66
173.10504@0.81100035	173.1050	0.81	HILIC	NEG			15040	6457	12209	4254	0.30433	-0.51	1.65	103881	33065	80382	27647	0.12755	-0.73	1.18
173.99422@1.4459996	173.9942	1.45	HILIC	POS			43121	12977	35900	8701	0.19946	-0.63	1.10							
173.99843@2.3369987	173.9984	2.34	RP	NEG			26903	15867	134080	64208	0.00031	1.48	1.93	336100	166466	2316217	868811	0.00002	1.67	2.79
174.01619@1.3260001	174.0162	1.33	HILIC	NEG			223177	24534	222883	24959	0.98026	-0.01	1.28							
174.01646@1.5270002	174.0165	1.53	HILIC	NEG			107942	46157	146606	60041	0.13993	0.67	0.92							
174.11172@6.8939986	174.1117	6.89	HILIC	POS	Arginine	2	228345	69289	280410	159047	0.36747	0.40	1.06	102719	17599	110730	36946	0.55408	0.27	0.46
174.13686@6.630998	174.1369	6.63	HILIC	POS			20370	3095	22027	4670	0.37982	0.40	0.97	4618	527	5036	2014	0.54100	0.27	0.47
175.06657@1.103	175.0666	1.10	HILIC	POS			47637	11344	44318	10470	0.53211	-0.30	1.19							
175.09573@6.2929997	175.0957	6.29	HILIC	NEG			33706	7180	32302	8097	0.70155	-0.18	1.54	39834	7765	35674	9813	0.32866	-0.45	0.74
175.09583@6.306	175.0958	6.31	HILIC	POS	Citrulline	2	362685	52239	371033	69617	0.77476	0.13	1.20	224795	51054	248425	76574	0.44446	0.35	0.75
175.96347@7.854003	175.9635	7.85	HILIC	NEG			149271	5543	147834	7549	0.64745	-0.21	1.12	167106	13756	170216	29227	0.77000	0.13	0.57
176.03238@3.432	176.0324	3.43	HILIC	NEG	Vitamin C	2	6536789	1221907	7142038	1461505	0.35141	0.43	0.78	11603	3340	7964	3401	0.03565	-0.95	1.88
176.04909@0.979	176.0491	0.98	HILIC	NEG			3877	1705	2863	1467	0.20142	-0.61	1.45	40732	20218	22589	18142	0.06498	-0.86	1.49
176.0947@1.6609997	176.0947	1.66	HILIC	POS	Serotonin	1	36306	8756	41475	13573	0.34223	0.43	0.90	180266	69626	633530	559124	0.02863	0.96	1.58
177.12689@0.7889997	177.1269	0.79	HILIC	POS			40454	22765	55901	42320	0.33738	0.43	0.62	2118	817	1783	1454	0.54561	-0.27	0.65
178.0414@6.6279993	178.0414	6.63	HILIC	POS			47257	6002	50425	9855	0.41249	0.37	0.73							
179.058@0.9929999	179.0580	0.99	HILIC	NEG			5765	2494	5202	2964	0.66715	-0.20	1.07	192625	197097	74653	42765	0.13244	-0.80	1.58
180.02623@1.9599993	180.0262	1.96	HILIC	POS			2157	1120	2112	1319	0.93740	-0.04	1.03	87498	14038	98630	31135	0.32917	0.44	0.91
180.06342@6.2010016	180.0634	6.20	HILIC	NEG			73610	11689	78995	19827	0.48323	0.32	1.00							
180.07877@8.164999	180.0788	8.16	RP	POS			168056	9112	163700	13218	0.42012	-0.37	0.76	163100	4751	163125	9286	0.99413	0.00	0.37
180.07896@9.554996	180.0790	9.55	RP	POS			256970	12337	257413	6114	0.92754	0.05	0.51	247232	8709	241676	8738	0.19676	-0.61	1.00
181.07458@5.078002	181.0746	5.08	HILIC	POS	Tyrosine	2	181942	34410	174671	37098	0.67225	-0.20	0.83	239394	48281	270452	51544	0.20513	0.59	1.14
181.98042@4.4279985	181.9804	4.43	HILIC	NEG			189679	10923	197435	24479	0.38460	0.39	0.70	211735	16622	217112	21124	0.55312	0.28	0.64
181.981@4.602999	181.9810	4.60	HILIC	NEG										96990	8838	95805	8702	0.77936	-0.13	0.39
181.99638@4.298	181.9964	4.30	RP	NEG			528151	18631	519426	24389	0.40100	-0.39	1.02	529174	12784	528689	13061	0.93777	-0.04	0.63
181.99835@3.1230006	181.9984	3.12	HILIC	POS			35033	12025	36518	20091	0.84836	0.09	1.21	9582	2267	10943	3859	0.36461	0.41	0.69
182.02483@1.6819996	182.0248	1.68	HILIC	NEG										29539	12551	16661	9303	0.02939	-1.02	1.66
182.0577@1.1220003	182.0577	1.12	HILIC	NEG			14590	4644	14657	5893	0.97889	0.01	1.13	51986	12186	44611	12292	0.22064	-0.58	0.95
183.06601@6.807	183.0660	6.81	HILIC	POS			431626	142473	744429	735277	0.21549	0.54	0.97							
183.06618@7.7010026	183.0662	7.70	HILIC	POS			15834	7973	27180	20283	0.12830	0.67	1.15							
183.06656@6.166	183.0666	6.17	HILIC	POS			127476	36656	109272	40954	0.33375	-0.45	1.69	5671	4070	5921	3722	0.89451	0.06	0.59
183.08682@4.419999	183.0868	4.42	HILIC	POS			242385	62485	279337	128400	0.43642	0.35	1.04							
184.0252@6.0650015	184.0252	6.07	HILIC	POS			35771	7484	37358	8325	0.67579	0.20	1.00	13479	3219	15176	5150	0.40499	0.38	0.70
184.11017@9.2890005	184.1102	9.29	RP	POS			72434	11903	76839	15875	0.50989	0.31	0.70	57826	10644	56625	10551	0.81404	-0.11	0.34

185.14021@10.007999	185.1402	10.01	RP	POS			86991	72432	83095	60574	0.90461	-0.06	0.63							
187.06297@2.5070012	187.0630	2.51	RP	POS			138171	22410	130584	25206	0.50826	-0.31	1.72	1030422	161971	1065379	117908	0.61683	0.25	1.31
187.06335@4.0419984	187.0634	4.04	HILIC	POS	Tryptophan fragment		92938	9514	87521	9730	0.25081	-0.54	1.78	731794	85531	745572	77065	0.72733	0.17	1.27
187.06384@2.1510005	187.0638	2.15	RP	POS										511092	83843	529944	64113	0.60747	0.25	1.26
187.12083@10.046999	187.1208	10.05	RP	POS			150682	127489	144178	110575	0.91066	-0.05	0.66							
187.12096@4.753001	187.1210	4.75	HILIC	POS			15566	4342	13630	3628	0.32797	-0.47	1.71	24506	4916	21719	8305	0.38872	-0.39	0.94
188.0142@3.579	188.0142	3.58	RP	NEG			70776	29678	64191	13908	0.57457	-0.29	0.90	1828929	294076	1553593	703542	0.28106	-0.48	1.31
188.04897@1.3779994	188.0490	1.38	HILIC	NEG			148794	56393	132334	51230	0.53059	-0.30	0.88	146458	95264	159023	165445	0.84244	0.09	0.60
188.1052@5.6309977	188.1052	5.63	RP	NEG	Azelaic acid	1	111561	37665	135290	70497	0.37449	0.40	0.71	68705	17053	70189	17553	0.85840	0.09	0.62
188.15257@6.718998	188.1526	6.72	HILIC	POS			148067	26341	140615	39919	0.64068	-0.21	1.11	27075	4788	28563	10954	0.70533	0.17	0.40
188.8684@0.9209999	188.8684	0.92	HILIC	NEG			40529	10399	46083	7573	0.22597	0.59	1.07	20589	6316	22279	9087	0.64797	0.21	0.69
190.0479@3.2219996	190.0479	3.22	HILIC	NEG			198207	30190	203795	65465	0.81372	0.11	1.16	27514	5233	24072	4305	0.15428	-0.68	1.33
190.17188@8.553	190.1719	8.55	RP	POS			235287	26043	240738	25921	0.66390	0.21	0.92	224303	166521	261036	90690	0.58542	0.28	0.45
190.17223@8.7890005	190.1722	8.79	RP	POS			136284	42239	136231	36470	0.99781	0.00	0.56	121150	93083	133999	62544	0.74305	0.16	0.27
190.17236@8.862	190.1724	8.86	RP	POS			31915	22968	30388	20213	0.88438	-0.07	0.47	29905	24576	28419	18231	0.88862	-0.07	0.21
190.17255@8.6449995	190.1726	8.64	RP	POS			301136	34924	291209	39732	0.58007	-0.26	0.52	290288	217216	340909	118114	0.56461	0.29	0.48
190.17271@8.714997	190.1727	8.71	RP	POS			133760	32247	133405	22463	0.97929	-0.01	0.63	115920	88363	133540	58366	0.63519	0.24	0.39
192.02686@2.445	192.0269	2.45	HILIC	NEG										296062	158541	439652	252019	0.15872	0.63	1.03
192.08029@1.1170003	192.0803	1.12	HILIC	NEG			102719	64086	74896	43339	0.31185	-0.50	0.64	21079	21314	9274	7954	0.16878	-0.71	1.24
193.07327@0.979	193.0733	0.98	HILIC	NEG	2-Methylhippuric acid	2	2691	855	5084	2724	0.02185	0.99	1.22	97976	61030	84065	34615	0.57594	-0.28	0.52
194.02078@1.9530001	194.0208	1.95	HILIC	NEG			21787	3272	21945	3631	0.92399	0.05	0.79	104715	9296	108861	12564	0.43186	0.36	0.87
195.11052@1.5289996	195.1105	1.53	HILIC	NEG			95289	39779	125366	68382	0.26014	0.51	0.99	15026	2551	18266	3232	0.02874	0.97	1.57
195.95366@6.738	195.9537	6.74	HILIC	NEG			404529	262382	672438	635126	0.24605	0.52	0.81							
196.05843@6.069	196.0584	6.07	HILIC	NEG	Gluconic acid	2	141675	41284	172884	70837	0.25963	0.51	1.02	48717	16135	65108	41639	0.27318	0.49	0.83
197.07765@6.306	197.0777	6.31	HILIC	POS			51368	6401	52554	8951	0.74745	0.15	1.02	8192	1802	8182	2221	0.99126	-0.01	0.89
197.93152@7.701001	197.9315	7.70	HILIC	NEG			44202	38472	68367	48022	0.25130	0.53	1.44	24117	5856	24622	13463	0.91656	0.05	0.41
198.16203@9.658998	198.1620	9.66	RP	POS			105616	8539	109564	10604	0.39309	0.40	1.23	145046	25008	134105	30606	0.41486	-0.38	0.63
199.15746@10.403	199.1575	10.40	RP	POS			115195	97146	103528	80503	0.78854	-0.13	0.62							
199.15756@6.194	199.1576	6.19	RP	POS			241784	179934	355818	171478	0.19054	0.62	0.73	4612	1338	3723	1080	0.14814	-0.69	1.36
199.15778@9.985	199.1578	9.99	RP	POS			103207	89513	99846	71809	0.93237	-0.04	0.63							
199.15897@9.882999	199.1590	9.88	RP	POS			37521	34164	24355	30067	0.40435	-0.40	0.82							
199.159@10.347	199.1590	10.35	RP	POS			53348	27524	47299	32548	0.67444	-0.20	0.72							
199.1941@9.204001	199.1941	9.20	RP	POS			63211	9595	67047	14181	0.50400	0.31	0.57	33058	21290	23792	9046	0.27583	-0.56	0.99
200.04486@2.4010007	200.0449	2.40	HILIC	NEG			125863	17607	158429	26824	0.00644	1.15	1.43	483311	65174	590551	111700	0.02131	0.99	1.81
201.10014@1.0620002	201.1001	1.06	HILIC	POS			28323	3241	31424	5732	0.16708	0.62	0.74							
201.11127@4.1840005	201.1113	4.18	HILIC	POS			5410	1048	4668	782	0.11747	-0.75	1.99	26131	6051	26556	2656	0.85784	0.09	0.41
201.13661@6.6329985	201.1366	6.63	RP	NEG			3412	1240	3906	1446	0.44566	0.36	0.97	54901	9222	66796	12235	0.03038	0.95	1.61
201.17308@1.4160002	201.1731	1.42	HILIC	POS			54085	43674	47045	33486	0.71229	-0.18	1.13	33215	39580	28256	11917	0.73992	-0.18	0.40
202.04234@2.408999	202.0423	2.41	HILIC	NEG			45317	3489	56004	10578	0.01059	1.09	1.32	156598	20660	191149	35038	0.01877	1.01	1.83
202.04526@6.209	202.0453	6.21	HILIC	POS			92284	21954	114934	48735	0.20964	0.56	1.01							
202.04556@5.222	202.0456	5.22	HILIC	POS			543295	105880	561086	137778	0.76034	0.14	0.67	6519612	964681	7111212	1371683	0.29804	0.48	0.88
202.14287@6.3379984	202.1429	6.34	HILIC	POS			14717	3679	18447	7247	0.17664	0.60	0.99	15563	3606	20763	6282	0.04219	0.89	1.47
202.14302@6.3990016	202.1430	6.40	HILIC	POS			47602	8266	52409	17175	0.44783	0.34	1.10	28268	5671	31981	13161	0.43475	0.35	0.70
203.05809@0.9799997	203.0581	0.98	HILIC	NEG										119629	51913	69847	38582	0.03991	-0.97	1.57
203.11598@3.0800014	203.1160	3.08	HILIC	POS	Acetyl-L-carnitine	2	10507275	3429908	10684843	5831559	0.93684	0.04	1.23	7738175	2286914	8278979	2495956	0.63808	0.22	0.47
204.08992@4.0269985	204.0899	4.03	HILIC	NEG	Tryptophan	2	7710	1142	6879	1037	0.12947	-0.72	2.03	110648	28980	114467	25569	0.77357	0.14	0.99
204.08997@2.5029988	204.0900	2.50	RP	POS	Tryptophan	1	312066	49315	298872	59028	0.61177	-0.24	1.63	2607248	434283	2695491	320184	0.63863	0.23	1.27
204.08998@2.1510005	204.0900	2.15	RP	NEG										290579	39099	293322	44325	0.89076	0.07	1.12
204.08998@2.5090008	204.0900	2.51	RP	NEG	Tryptophan	2	73359	10053	67463	14610	0.32549	-0.45	1.67	540934	95832	542297	70987	0.97372	0.02	1.16
204.09013@4.039999	204.0901	4.04	HILIC	POS	Tryptophan	2	219854	23752	209029	26205	0.37118	-0.42	1.73	1768315	221186	1794573	195383	0.79554	0.13	1.24
204.09048@2.1440005	204.0905	2.14	RP	POS										1273826	210023	1324338	164689	0.58584	0.27	1.27
206.02193@0.97999966	206.0219	0.98	HILIC	NEG			51119	35338	32123	34157	0.32386	-0.53	0.77	36159	70865	40896	121405	0.92206	0.05	0.43
206.11603@0.861	206.1160	0.86	HILIC	POS										186	88	17304	8359	0.00007	1.62	2.55
206.16705@9.451997	206.1671	9.45	RP	NEG	2,6-Di-tert-butylphenol	2	99363	3298	92204	4755	0.00147	-1.31	1.81	89012	3167	88953	3948	0.97230	-0.02	0.31
207.08945@0.8500002	207.0895	0.85	HILIC	NEG			3134	762	2994	986	0.73953	-0.16	1.32	33592	3617	34563	6250	0.68519	0.18	0.32
208.02141@0.9760002	208.0214	0.98	HILIC	NEG			4545	2055	2779	1452	0.05911	-0.90	1.27	33610	13062	18102	9616	0.01383	-1.13	1.92
208.08495@4.0529995	208.0850	4.05	HILIC	POS			9286	4673	7395	2381	0.31944	-0.51	1.63	24810	2830	25635	4141	0.62262	0.23	0.49
208.18309@8.7890005	208.1831	8.79	RP	POS			42835	11541	41787	8691	0.83424	-0.10	0.63	36987	26922	40934	18527	0.72938	0.17	0.30
208.18321@8.645997	208.1832	8.65	RP	POS			296228	30102	284430	36907	0.46474	-0.34	0.58	256376	195911	298994	102689	0.58817	0.28	0.45
208.18327@8.553	208.1833	8.55	RP	POS			237317	25670	240409	28056	0.81034	0.11	0.82	204096	153341	235551	82843	0.61116	0.26	0.42
208.18327@8.714997	208.1833	8.71	RP	POS			46955	9472	47052	7463	0.98137	0.01	0.79	40352	30627	45795	19830	0.67068	0.21	0.36
209.94171@3.3180003	209.9417	3.32	HILIC	NEG			270397	26621	267035	33682	0.81573	-0.11	0.99	302622	32225	288071	40952	0.40997	-0.38	0.97
209.9419@3.2319992	209.9419	3.23	HILIC	NEG										88312	3311	84730	4017	0.05275	-0.87	1.59

210.0741@2.4110007	210.0741	2.41	HILIC	NEG			17005	2470	22207	5211	0.01378	1.05	1.15	92813	11869	129368	22712	0.00047	1.40	2.44
212.04684@6.292	212.0468	6.29	HILIC	NEG			15593	2942	19013	6489	0.15837	0.62	0.85							
213.00943@2.480001	213.0094	2.48	RP	NEG	Indoxyl sulfate	1	66675	14994	154374	60367	0.00091	1.39	1.66	1223343	198287	2368606	736377	0.00053	1.43	2.37
213.05139@6.3029976	213.0514	6.30	HILIC	POS			25132	4118	24653	4919	0.82481	-0.10	1.12	1623	728	1761	633	0.67876	0.20	0.82
213.08287@6.671998	213.0829	6.67	RP	POS			341777	16310	343648	10646	0.78362	0.14	0.92	371296	12839	378458	9065	0.20364	0.62	1.10
213.1729@9.9609995	213.1729	9.96	RP	POS			280958	249256	276889	208743	0.97099	-0.02	0.63							
213.17316@6.886997	213.1732	6.89	RP	POS			339371	259447	506843	270355	0.19888	0.60	0.72	5131	1057	4927	954	0.67644	-0.20	0.65
213.17339@9.9609995	213.1734	9.96	RP	POS			280158	253146	276207	208780	0.97212	-0.02	0.62							
214.05635@6.057003	214.0564	6.06	HILIC	NEG			29768	2786	30844	3058	0.44606	0.36	0.69	25609	3876	26288	4596	0.73789	0.16	0.65
215.05573@6.4140005	215.0557	6.41	HILIC	NEG			309076	61897	292589	52563	0.55708	-0.29	0.94	752	351	780	309	0.86181	0.08	0.66
215.05602@6.4140005	215.0560	6.41	HILIC	POS			97992	25116	84552	12136	0.19233	-0.66	1.44							
215.88742@0.9209999	215.8874	0.92	HILIC	NEG			18714	12366	34166	14055	0.02342	1.00	1.31	35087	12426	55565	25699	0.04243	0.88	1.55
216.04016@6.2010016	216.0402	6.20	HILIC	NEG			707678	81693	717437	143683	0.85839	0.08	0.76							
216.04033@5.1159983	216.0403	5.12	HILIC	NEG			8461677	1084657	8654964	1420571	0.74726	0.15	0.87	16213580	2052532	16654072	2358838	0.67715	0.20	0.75
216.08002@0.972	216.0800	0.97	HILIC	NEG			1035	981	519	391	0.19098	-0.68	1.78	43291	19825	19112	9972	0.01474	-1.26	2.09
217.03854@4.61	217.0385	4.61	HILIC	NEG										42954	8845	39746	12758	0.53778	-0.28	0.51
217.13158@2.1500008	217.1316	2.15	HILIC	POS			330138	93982	393256	104325	0.19461	0.60	0.83	173422	48064	224766	50699	0.04170	0.92	1.51
218.03745@6.2010016	218.0375	6.20	HILIC	NEG			209318	26064	209717	37978	0.97926	0.01	0.88							
218.12611@5.754999	218.1261	5.75	HILIC	POS										13606	4787	15376	5379	0.47042	0.34	0.65
219.11057@1.0680004	219.1106	1.07	HILIC	NEG	Pantothenic acid	2	3445132	598371	3855699	817429	0.23496	0.54	0.77	192570	73590	181488	71222	0.75137	-0.15	0.45
219.11069@2.3259988	219.1107	2.33	RP	NEG	Pantothenic acid	1	713720	118494	791967	196971	0.31201	0.46	0.60	10630	2283	11208	3021	0.64925	0.21	0.39
219.11089@2.327001	219.1109	2.33	RP	POS	Pantothenic acid	1	607931	108531	719391	189383	0.13615	0.66	0.81	9853	2511	11389	2787	0.23538	0.55	0.90
219.11101@1.0629998	219.1110	1.06	HILIC	POS			1424956	170316	1597985	273386	0.11885	0.70	0.81	41149	9253	44231	11864	0.54355	0.28	0.52
222.01955@1.3870002	222.0196	1.39	HILIC	NEG			284643	106626	304224	89766	0.68383	0.20	0.78	222067	92606	259210	44710	0.32009	0.51	1.07
222.1985@8.645997	222.1985	8.65	RP	POS			88289	9561	89211	11926	0.85755	0.08	0.65	85413	65934	97079	35182	0.65987	0.23	0.37
222.19891@8.553	222.1989	8.55	RP	POS			67249	8294	70975	8798	0.36908	0.42	1.38	65433	49758	74506	27393	0.65200	0.23	0.37
222.95422@12.438002	222.9542	12.44	RP	NEG										376263	42153	357246	52803	0.40666	-0.39	0.90
223.10548@4.405999	223.1055	4.41	HILIC	NEG			171778	189113	250490	407389	0.59570	0.24	0.95							
223.12083@5.416	223.1208	5.42	HILIC	POS			49144	2477	50140	2323	0.39628	0.41	1.38	50166	1743	51313	3307	0.35889	0.41	0.73
224.02757@3.270998	224.0276	3.27	HILIC	POS			37101	10001	43943	11455	0.19334	0.60	0.89	106056	7405	103328	7780	0.45778	-0.35	0.79
225.07559@1.7400005	225.0756	1.74	HILIC	POS			8943	2567	8082	3721	0.56983	-0.26	1.37	40245	5307	38134	6322	0.45140	-0.35	0.60
225.20958@9.463003	225.2096	9.46	RP	POS			59357	39570	73943	39002	0.44491	0.37	0.81	52331	60132	76986	60584	0.40081	0.40	0.72
225.94452@4.4250016	225.9445	4.43	HILIC	POS			154138	33429	183285	55471	0.18627	0.59	0.80	533233	20639	548489	32458	0.24214	0.53	0.90
226.06891@4.4369993	226.0689	4.44	HILIC	NEG			57070	10009	63808	16642	0.30306	0.47	1.07							
226.06892@6.201003	226.0689	6.20	HILIC	NEG			452495	84049	468466	119934	0.74419	0.15	1.16	4370	1002	6010	2676	0.09671	0.73	1.21
226.06892@5.147002	226.0689	5.15	HILIC	NEG			231701	35540	240408	53272	0.68371	0.19	0.85	2057226	394553	2228639	532768	0.44348	0.35	0.78
226.07198@4.0630007	226.0720	4.06	HILIC	POS										37680	8607	37236	9521	0.91853	-0.05	1.04
226.19305@9.994997	226.1931	9.99	RP	NEG			60394	18435	71966	18794	0.20670	0.59	0.91	8393	2838	6374	1517	0.09536	-0.83	1.36
227.09106@2.2060008	227.0911	2.21	HILIC	POS	Deoxycytidine	2	20696	4707	20417	5085	0.90552	-0.06	1.44	203506	42347	192413	22656	0.51703	-0.33	0.62
227.1889@7.4800005	227.1889	7.48	RP	POS			299971	79440	352030	84493	0.19642	0.60	0.72	157669	15515	158172	17911	0.94990	0.03	0.47
227.22537@9.873995	227.2254	9.87	RP	POS			268800	39259	289800	67136	0.41948	0.37	0.86	48373	28628	57933	27996	0.48680	0.33	0.54
228.00327@1.316	228.0033	1.32	HILIC	NEG			2467308	145966	2490422	248754	0.80893	0.11	0.77							
228.00343@0.9810001	228.0034	0.98	HILIC	NEG			83390	39452	101043	36357	0.34290	0.46	0.79							
228.07446@0.9810001	228.0745	0.98	HILIC	NEG			3196	1735	3388	1341	0.80029	0.13	0.84	40013	14999	38634	11441	0.83293	-0.10	0.42
228.2087@10.342003	228.2087	10.34	RP	NEG			252689	64892	305299	70563	0.11790	0.72	0.92	48714	11630	41346	7403	0.14356	-0.72	1.35
229.08856@5.393998	229.0886	5.39	HILIC	POS			129070	21846	152180	36891	0.11728	0.70	0.97	2229	1100	3009	1996	0.30861	0.46	0.75
229.16843@9.962001	229.1684	9.96	RP	POS			76946	66125	78055	53265	0.96982	0.02	0.61							
230.00064@1.316	230.0006	1.32	HILIC	NEG			780687	48926	786806	78300	0.84183	0.09	0.81							
230.0194@6.768	230.0194	6.77	HILIC	NEG	D-Ribose 5-phosphate	2	183146	77735	274395	209068	0.22438	0.54	0.91							
231.14706@1.6199996	231.1471	1.62	HILIC	POS	Isobutyryl carnitine	2	492198	231202	465860	74078	0.76358	-0.16	0.89	179984	43942	217626	55732	0.12652	0.70	1.63
231.99377@5.576999	231.9938	5.58	RP	NEG			1057851	66172	1058619	54421	0.97923	0.01	1.06	1143869	52350	1159229	41052	0.50727	0.33	0.78
232.00381@0.98899984	232.0038	0.99	HILIC	NEG			16997	10359	24841	16160	0.22890	0.54	0.72	202980	83742	546436	310802	0.00597	1.17	1.95
232.05235@6.630998	232.0524	6.63	HILIC	POS			16907	2217	18788	3310	0.16794	0.62	0.82							
232.08339@0.9820002	232.0834	0.98	HILIC	NEG										28787	11774	16418	8174	0.02458	-1.06	1.73
232.93892@6.0899973	232.9389	6.09	HILIC	NEG			367106	43605	375799	65765	0.74104	0.15	1.00	278588	29896	281363	35585	0.85940	0.08	0.75
232.93907@6.328002	232.9391	6.33	HILIC	NEG			1375262	102437	1494678	155805	0.06713	0.81	1.05	993584	130920	1004464	159023	0.87530	0.07	0.70
232.93909@5.725002	232.9391	5.73	HILIC	NEG			116628	10183	125516	15340	0.15905	0.64	0.92	90216	11203	92562	12097	0.67521	0.20	0.58
232.93929@5.8480034	232.9393	5.85	HILIC	NEG			240898	22866	253889	50362	0.47848	0.32	1.04	137216	17661	142293	15484	0.53135	0.30	0.82
234.16222@8.860995	234.1622	8.86	RP	POS			177709	8697	175691	8371	0.62537	-0.24	1.09	183400	9157	180471	16987	0.64677	-0.21	0.52
236.14143@8.164999	236.1414	8.16	RP	POS			144836	6993	137268	12589	0.12566	-0.68	1.13	151973	7439	149797	7859	0.55516	-0.28	0.48
236.1416@9.554996	236.1416	9.55	RP	POS			197280	6773	196260	7459	0.76496	-0.14	0.76	180230	7136	177527	5624	0.39500	-0.42	0.86
236.93327@12.438998	236.9333	12.44	RP	NEG										87220	14335	81975	14363	0.45137	-0.36	0.86
237.03789@6.4140005	237.0379	6.41	HILIC	POS			24736	3114	22932	2665	0.21194	-0.60	1.06							

237.93651@3.3069997	237.9365	3.31	HILIC	NEG			37039	7383	38520	9111	0.70775	0.18	0.81	36080	3322	36032	3303	0.97625	-0.01	0.59
238.15672@9.411997	238.1567	9.41	RP	POS			6383	1262	7637	1099	0.04153	0.94	1.27	122364	32728	135255	25692	0.37638	0.43	1.25
240.02087@5.5930004	240.0209	5.59	HILIC	NEG			12888	11033	23929	23490	0.20812	0.56	0.83							
240.12276@7.033001	240.1228	7.03	HILIC	POS			13844	9083	21401	24457	0.38296	0.39	1.08	1014	249	1241	552	0.26506	0.50	0.84
241.07185@3.1230006	241.0719	3.12	HILIC	POS			227831	73821	236689	129122	0.85720	0.08	1.24	59690	17024	67989	21311	0.37052	0.42	0.73
241.09282@1.0729996	241.0928	1.07	HILIC	POS			104552	23723	124108	42566	0.23513	0.53	0.73	3158	1836	3811	1474	0.42638	0.39	0.69
241.27797@7.326001	241.2780	7.33	RP	POS			568484	32231	575622	52310	0.72670	0.16	0.96	1553429	389546	1574253	372620	0.90989	0.05	0.65
242.04578@4.039001	242.0458	4.04	HILIC	POS			2405	888	2197	925	0.63410	-0.23	1.41	44102	8507	48273	10985	0.37609	0.41	1.10
242.18796@9.339997	242.1880	9.34	RP	NEG			16433	2936	16118	2625	0.81579	-0.11	1.69	335620	58563	330174	78758	0.86836	-0.08	0.60
242.90607@0.9209999	242.9061	0.92	HILIC	NEG			5271	6696	13722	8792	0.03269	0.94	1.13	33938	13306	108425	77610	0.01282	1.08	1.80
243.08607@3.5640006	243.0861	3.56	HILIC	POS	Cytidine	2	128655	44363	160570	93814	0.35673	0.41	1.11	193324	25925	191477	43753	0.91273	-0.05	0.56
243.08685@6.1510005	243.0869	6.15	HILIC	NEG			106362	34146	106546	62302	0.99374	0.00	1.15	3001	1914	3327	2613	0.76358	0.14	0.57
243.18398@7.3670015	243.1840	7.37	RP	POS			70551	16137	79143	15571	0.27020	0.52	0.69	50233	6872	52811	10190	0.53121	0.29	0.65
243.22005@8.553	243.2201	8.55	RP	POS			69361	6504	70246	8122	0.80019	0.12	0.65	68449	50401	81901	27764	0.51136	0.34	0.55
243.22035@8.645997	243.2204	8.65	RP	POS			99526	8956	94448	13882	0.36114	-0.42	0.62	102383	76305	121083	40895	0.54403	0.31	0.50
243.2569@7.798002	243.2569	7.80	RP	POS			24630	8394	26395	12892	0.73047	0.16	0.72	112989	36509	98377	21169	0.33448	-0.49	1.12
244.0693@3.6480002	244.0693	3.65	HILIC	NEG			49132	7823	55150	12831	0.23714	0.53	0.92	656844	98603	692114	105094	0.47366	0.34	0.66
244.06944@1.5180006	244.0694	1.52	HILIC	NEG	Uridine	2	585329	184514	647328	176258	0.47989	0.34	1.08	116830	24332	122193	22867	0.63938	0.23	0.62
245.08788@4.366	245.0879	4.37	HILIC	POS			79425	77941	54272	93406	0.55475	-0.29	1.40							
245.163@1.3069994	245.1630	1.31	HILIC	POS			218268	166600	213789	56732	0.94352	-0.04	0.71	20561	3987	24285	5118	0.09980	0.74	1.39
245.17392@6.7349997	245.1739	6.73	HILIC	POS			63582	8564	68110	17627	0.48620	0.31	1.18	1266	375	1792	791	0.08290	0.76	1.26
245.97092@6.0899973	245.9709	6.09	HILIC	NEG			26508	2616	29180	3809	0.09589	0.75	0.90	12509	2388	14090	2918	0.22246	0.56	0.97
245.99278@4.2989974	245.9928	4.30	RP	NEG			1316196	19544	1321225	20057	0.59864	0.25	1.11	1442243	33157	1446141	33802	0.80883	0.12	0.61
246.05026@6.175002	246.0503	6.18	HILIC	NEG			196573	64745	227085	120918	0.50343	0.30	1.00	1322	518	2110	788	0.02046	1.00	1.77
246.10065@0.8489995	246.1007	0.85	HILIC	NEG			2001	610	1722	470	0.30370	-0.50	1.35	20901	6333	18984	4800	0.48974	-0.34	0.96
246.10078@0.8500002	246.1008	0.85	HILIC	POS										15162	3540	14741	4070	0.81717	-0.11	1.08
246.23529@10.484995	246.2353	10.48	RP	POS			79435	5764	80761	6073	0.64163	0.22	0.53	92488	19947	102975	21244	0.29619	0.49	1.05
247.10596@6.168999	247.1060	6.17	HILIC	POS			28728	8629	32275	10635	0.44483	0.36	1.07							
247.14194@4.2429976	247.1419	4.24	HILIC	POS	Azatadine	2	427691	136017	402600	191433	0.74931	-0.15	1.53	84321	29926	77738	27988	0.63943	-0.23	0.43
247.16116@1.0119998	247.1612	1.01	HILIC	POS			1938	435	2034	711	0.72850	0.16	1.19	32786	10354	32706	11049	0.98757	-0.01	0.74
248.17563@8.553996	248.1756	8.55	RP	POS			76186	6974	72983	7525	0.36249	-0.43	1.00	56944	38430	67666	22859	0.49888	0.34	0.57
248.17586@8.645997	248.1759	8.65	RP	POS			74277	16316	61510	13384	0.09410	-0.79	1.07	70916	49970	81899	26522	0.58513	0.28	0.46
248.17822@8.971	248.1782	8.97	RP	POS			85008	3073	83738	5331	0.53535	-0.28	1.24	88099	5575	92379	8336	0.21034	0.57	0.97
248.25082@10.803994	248.2508	10.80	RP	POS			5719	1143	5443	1389	0.64892	-0.21	1.50	68328	6200	65415	9688	0.44987	-0.35	0.80
249.96771@5.4100027	249.9677	5.41	HILIC	NEG			38791	2646	36477	5174	0.23781	-0.53	0.91	35832	1120	35176	2274	0.43671	-0.35	0.64
249.9679@4.386001	249.9679	4.39	HILIC	NEG			177852	16582	191083	30769	0.26127	0.50	0.78	378809	33128	385055	39775	0.72052	0.17	0.51
250.06831@0.97999966	250.0683	0.98	HILIC	NEG			2004	755	1624	803	0.31587	-0.47	2.17	33890	11946	17408	7299	0.00472	-1.30	2.18
250.12111@7.920001	250.1211	7.92	RP	POS			57540	4253	59001	5046	0.51365	0.31	0.92	65731	1657	66344	3196	0.60723	0.23	0.67
251.07068@5.4010005	251.0707	5.40	HILIC	POS			125066	21334	143791	30615	0.14477	0.66	0.94	733	439	1185	1147	0.27173	0.49	0.84
252.02629@5.330998	252.0263	5.33	HILIC	NEG			61239	2795	63524	3760	0.15688	0.64	0.99	6417	824	6980	2130	0.45615	0.33	0.81
252.20935@8.7890005	252.2094	8.79	RP	POS			104823	27701	104915	21942	0.99399	0.00	0.57	99179	77369	109137	51928	0.75977	0.15	0.25
252.20961@8.714997	252.2096	8.71	RP	POS			119925	33273	121869	24620	0.89233	0.07	0.71	113777	88786	129034	58637	0.68229	0.21	0.34
252.9248@5.722	252.9248	5.72	HILIC	NEG			20375	1528	21381	1771	0.21236	0.58	0.76	19882	1679	19729	1786	0.85457	-0.09	0.55
253.07393@6.281999	253.0739	6.28	HILIC	NEG			17054	4051	18061	4007	0.60510	0.25	0.90							
253.16824@8.164999	253.1682	8.16	RP	POS			589883	37198	571999	47477	0.38208	-0.41	0.97	657440	25473	638179	24649	0.12438	-0.72	1.21
253.24097@10.036003	253.2410	10.04	RP	POS	Palmitoleamide	2	1092056	260502	1139688	417903	0.77103	0.13	0.56	49184	46181	62455	42334	0.53830	0.30	0.55
254.02667@0.9810001	254.0267	0.98	HILIC	NEG										27854	12091	14711	7650	0.01917	-1.11	1.86
254.22435@10.454003	254.2244	10.45	RP	POS			97323	17937	115532	29149	0.12207	0.69	0.96	159844	12080	147981	12064	0.05372	-0.88	1.47
254.22437@9.618998	254.2244	9.62	RP	POS			62406	10297	65513	9003	0.51101	0.32	0.93	29862	5128	25397	5652	0.09676	-0.76	1.26
254.22466@10.458994	254.2247	10.46	RP	NEG	Palmitoleic acid (FA 16:1)	1	1186793	311663	1442734	322362	0.10601	0.75	0.95	650417	121116	502487	116591	0.01809	-1.06	1.75
254.22523@9.381002	254.2252	9.38	RP	POS			162582	22717	174097	25340	0.32377	0.46	0.84	115533	15014	107750	17498	0.32357	-0.46	0.92
254.22556@9.484001	254.2256	9.48	RP	POS			63313	12557	62780	7740	0.91799	-0.05	0.76	32748	7390	31459	15733	0.82166	-0.10	0.37
254.92236@5.7239995	254.9224	5.72	HILIC	NEG			23967	2142	25249	2227	0.23182	0.56	0.82	23331	2863	23773	2605	0.73902	0.16	0.46
255.02272@0.91999996	255.0227	0.92	HILIC	NEG			51721	22639	73605	23656	0.06161	0.85	1.06							
255.08723@1.0709995	255.0872	1.07	HILIC	NEG			434585	35214	450090	51987	0.46210	0.34	0.77	37788	13613	35364	13061	0.70717	-0.18	0.49
255.25696@10.364004	255.2570	10.36	RP	POS	Palmitic acid	1	7043455	1218570	7303874	1614240	0.70123	0.18	0.85	93061	17625	84131	15023	0.27148	-0.53	0.91
255.29337@7.3919992	255.2934	7.39	RP	POS			224565	6550	224956	11049	0.92696	0.04	0.96	245491	7027	246884	6012	0.66238	0.21	0.75
256.14026@3.8430011	256.1403	3.84	HILIC	POS			68086	58673	119490	113136	0.23218	0.53	0.98	36244	11461	149918	61647	0.00014	1.55	2.53
256.24@10.216002	256.2400	10.22	RP	NEG			33993	6589	39016	6374	0.12149	0.72	0.90	167757	7846	164060	12711	0.45945	-0.34	0.77
256.24057@10.730002	256.2406	10.73	RP	NEG	FA 16:0	2	1166845	322294	1572762	487474	0.04889	0.87	1.03	407772	62488	341372	60668	0.03662	-0.95	1.66
256.24298@9.349	256.2430	9.35	RP	POS			143274	24877	219284	117835	0.07237	0.79	1.51	3377	2016	3951	1968	0.55255	0.29	0.58
257.06097@2.3240001	257.0610	2.32	RP	POS			58933	8553	66094	16341	0.24939	0.52	0.77							
257.0666@3.9149995	257.0666	3.91	HILIC	NEG			86072	23633	105662	29530	0.13539	0.68	0.93	132243	27604	134860	32921	0.85655	0.09	0.67

257.103@6.1690025	257.1030	6.17	HILIC	POS	Glycerophosphocholine	2	14059081	4531668	12051870	5511171	0.40765	-0.39	1.63	460318	363593	503255	374900	0.80892	0.12	0.59
258.1837@9.2890005	258.1837	9.29	RP	POS			337391	62554	360299	74189	0.48653	0.33	0.73	314735	57334	297037	54577	0.51523	-0.31	0.63
259.17868@1.0929998	259.1787	1.09	HILIC	POS			71435	18443	78492	31741	0.56344	0.26	0.98	80758	25858	70556	24388	0.40578	-0.40	0.81
260.09854@5.7239995	260.0985	5.72	HILIC	NEG			19500	1085	20374	2732	0.37123	0.40	0.79	21164	3460	22411	4382	0.50827	0.31	0.62
260.1376@5.673001	260.1376	5.67	HILIC	POS			50714	9935	58403	17031	0.24861	0.52	1.08	2274	483	2798	1173	0.22020	0.54	0.90
260.98907@5.3840017	260.9891	5.38	HILIC	NEG			29963	1240	30735	1179	0.19771	0.61	1.83	9079	1061	8878	1182	0.70834	-0.18	0.44
261.12106@5.4400005	261.1211	5.44	HILIC	POS			48335	11446	63760	13246	0.01640	1.05	1.21							
261.15784@3.5989995	261.1578	3.60	HILIC	POS			125652	22798	130835	35082	0.71003	0.17	1.29	29668	4894	34925	5498	0.04620	0.90	1.45
262.2292@10.251005	262.2292	10.25	RP	POS			247742	40138	234792	64360	0.60841	-0.23	0.65	14568	3396	8507	3649	0.00193	-1.30	2.11
262.2293@10.332	262.2293	10.33	RP	POS			28043	7870	41029	11609	0.01156	1.08	1.39	23482	9256	12759	6500	0.01504	-1.13	1.85
262.23004@10.584998	262.2300	10.58	RP	POS			51204	9141	63377	12937	0.03152	0.94	1.04	13336	3035	12147	2859	0.40877	-0.40	0.92
264.0475@1.337	264.0475	1.34	HILIC	NEG			60024	11589	65225	13503	0.39106	0.40	1.00							
264.04767@1.5179998	264.0477	1.52	HILIC	NEG			87352	43822	113245	62707	0.31738	0.46	1.00							
264.05106@0.979	264.0511	0.98	HILIC	NEG			14167	9118	14839	7128	0.86656	0.08	0.81	127312	60501	114202	40609	0.60783	-0.26	0.57
264.2454@10.5599985	264.2454	10.56	RP	POS			59388	15333	70930	15513	0.13259	0.70	0.90	94738	18528	77015	15953	0.04794	-0.92	1.51
264.2457@10.477998	264.2457	10.48	RP	POS			174689	20013	177382	23632	0.79651	0.12	0.66	18701	3209	16640	4805	0.29185	-0.48	0.96
265.07935@4.5080004	265.0794	4.51	HILIC	NEG			111663	17709	119729	25097	0.43548	0.36	1.01							
266.02615@1.7520002	266.0262	1.75	HILIC	NEG			58964	10644	62988	11461	0.45134	0.36	1.01							
266.1555@10.078002	266.1555	10.08	RP	NEG	Dodecyl sulfate	2	971175	556465	1437695	906526	0.19738	0.58	0.81	458680	112174	457183	105587	0.97731	-0.01	0.52
267.04453@5.39	267.0445	5.39	HILIC	POS			18006	3195	20038	4569	0.28262	0.49	1.00							
267.09732@1.4270008	267.0973	1.43	RP	POS	Adenosine	1	1788524	569056	1913669	804926	0.70429	0.18	1.23	1474	1164	2939	1960	0.06581	0.81	1.34
267.09732@1.4069997	267.0973	1.41	HILIC	POS			2418071	544751	2343324	560656	0.77862	-0.13	1.23							
268.08035@1.3559996	268.0804	1.36	RP	NEG			456610	173353	501172	351095	0.73011	0.16	1.09							
268.08044@2.563	268.0804	2.56	HILIC	NEG	Inosine	2	1679285	593655	1865486	1194494	0.67255	0.19	1.17							
268.08066@1.4780003	268.0807	1.48	RP	NEG	Inosine	2	841105	312571	982070	693357	0.57466	0.25	1.05							
268.08075@2.5719998	268.0808	2.57	HILIC	POS			1092879	394185	1155118	598248	0.79422	0.12	1.27							
268.08185@1.4850003	268.0819	1.49	RP	POS			490324	183415	502660	331957	0.92160	0.04	1.28							
268.23972@10.638	268.2397	10.64	RP	NEG	FA 17:1	2	104546	20408	122656	21091	0.08244	0.80	0.94	28109	3307	24836	3060	0.04608	-0.92	1.54
268.96912@3.361	268.9691	3.36	HILIC	POS			13247	4907	15739	4675	0.28972	0.50	1.07	41099	2528	40596	1977	0.65193	-0.22	0.86
269.2718@10.329996	269.2718	10.33	RP	POS			379527	10476	384578	10443	0.32288	0.47	1.01	101328	10554	103524	9350	0.65082	0.22	0.54
269.27216@10.566003	269.2722	10.57	RP	POS			54832	8216	55320	12284	0.92101	0.05	1.03	18956	2082	18158	3583	0.56267	-0.26	0.62
270.0618@3.4499989	270.0618	3.45	HILIC	NEG			17738	2594	24588	17063	0.23771	0.52	1.27	52226	16223	137563	226953	0.26297	0.49	0.81
271.95276@1.4279997	271.9528	1.43	HILIC	NEG			91974	9512	95662	11402	0.46415	0.34	0.69							
272.01138@5.2879987	272.0114	5.29	HILIC	NEG			60198	2387	55844	4147	0.01278	-1.07	1.39	57311	10320	55772	9758	0.75152	-0.15	0.73
272.01215@5.365001	272.0122	5.37	HILIC	NEG			73093	4096	73036	3113	0.97440	-0.02	1.18	44708	6928	46078	9619	0.72986	0.16	0.51
272.2348@10.149996	272.2348	10.15	RP	NEG			42847	7944	45232	7792	0.53165	0.30	0.93	44539	4385	45782	13782	0.79245	0.12	0.68
272.23486@9.483001	272.2349	9.48	RP	NEG			107365	15711	103376	16114	0.60339	-0.25	0.98	24761	6762	20689	5015	0.17710	-0.66	1.21
272.235@9.615998	272.2350	9.62	RP	NEG			67536	9694	68520	10634	0.83989	0.10	1.01	25844	4858	22628	4518	0.16865	-0.65	1.18
272.235@9.428995	272.2350	9.43	RP	NEG	Juniperic acid	2	65139	11102	74963	9874	0.06763	0.85	1.16	37477	6547	34027	8230	0.33504	-0.45	0.87
272.23523@9.381002	272.2352	9.38	RP	NEG			68730	8175	68269	7797	0.90489	-0.06	1.28	44694	7923	39562	5856	0.14818	-0.70	1.19
272.23746@9.479003	272.2375	9.48	RP	POS			33992	10026	35866	12907	0.73295	0.16	0.94	73449	28455	75806	53868	0.90670	0.05	0.37
273.0785@5.39	273.0785	5.39	HILIC	POS			30688	6092	34333	7724	0.27776	0.50	0.89							
273.09583@6.627	273.0958	6.63	HILIC	NEG			22998	3157	23509	4169	0.77058	0.14	0.59							
273.9509@1.427	273.9509	1.43	HILIC	NEG			93832	10409	97264	13514	0.55043	0.28	0.95							
273.96606@7.3159995	273.9661	7.32	HILIC	NEG			57495	15409	47499	10775	0.14243	-0.72	1.17	19651	5261	23582	15733	0.47277	0.32	0.57
274.04544@7.133001	274.0454	7.13	HILIC	NEG			20998	7169	45661	42009	0.09617	0.73	0.95							
274.07922@0.97999966	274.0792	0.98	HILIC	NEG			3444	1778	3849	1649	0.62670	0.24	0.79	63363	23604	59447	15943	0.69424	-0.20	0.49
274.13522@1.9249996	274.1352	1.92	HILIC	POS			101225	75939	163104	115705	0.19014	0.59	0.80							
275.1732@2.4339988	275.1732	2.43	HILIC	POS			79433	13492	84245	21247	0.56683	0.26	0.98	11500	2504	10636	1239	0.39140	-0.44	0.73
276.06137@5.6729984	276.0614	5.67	HILIC	NEG			39077	7344	53677	14529	0.01411	1.05	1.24							
276.17322@8.841002	276.1732	8.84	RP	POS			45965	4147	47114	4199	0.56866	0.27	0.84	69971	9578	70008	15331	0.99498	0.00	0.31
278.0668@0.84000003	278.0668	0.84	HILIC	NEG			19605	3472	20465	3669	0.61726	0.24	1.05	82526	22886	80216	22335	0.83229	-0.10	0.27
278.15222@9.208005	278.1522	9.21	RP	POS			93256	10090	92069	6091	0.77462	-0.15	1.16	151550	83009	107993	36858	0.19641	-0.66	1.09
278.15234@9.287996	278.1523	9.29	RP	POS	Phthalic acid Mono-2-ethylhexyl Ester	2	68586	7917	71669	6161	0.38043	0.43	0.95	112129	58621	81895	29315	0.20954	-0.64	1.05
278.18765@8.891	278.1877	8.89	RP	NEG			50446	2746	49851	3246	0.67861	-0.20	0.59	51720	5118	52043	7101	0.91204	0.05	0.72
278.1898@10.730002	278.1898	10.73	RP	NEG			129718	11313	136904	10701	0.18839	0.62	1.06	76607	14984	69266	11998	0.27752	-0.53	1.11
278.2191@10.802998	278.2191	10.80	RP	POS										197615	31034	192097	49216	0.77547	-0.13	0.49
278.22437@10.372995	278.2244	10.37	RP	NEG	FA 18:3	1	489777	128236	676243	173433	0.01739	1.03	1.12	117468	20553	78221	23345	0.00136	-1.33	2.18
278.2256@9.493998	278.2256	9.49	RP	POS			988644	670495	1255138	864012	0.47079	0.34	0.93	281339	288773	341958	589287	0.77928	0.13	0.42
279.06186@3.5250003	279.0619	3.53	HILIC	NEG			47397	16694	62062	35191	0.26262	0.50	0.93	167852	40716	222818	61807	0.03660	0.92	1.49
279.08505@6.1790004	279.0851	6.18	HILIC	POS			298933	59705	276701	44468	0.39513	-0.42	1.52	13090	13033	14418	15089	0.84364	0.09	0.53
279.25583@10.247995	279.2558	10.25	RP	POS	Linoleamide	2	9391786	1624302	8864972	2904729	0.63325	-0.22	0.69	24104	16431	26826	13608	0.71158	0.18	0.42
279.2561@9.349	279.2561	9.35	RP	POS			147970	31182	230659	133813	0.08444	0.76	1.41	4606	2599	6080	3305	0.30378	0.48	0.78

279.25717@9.197	279.2572	9.20	RP	POS			108597	16946	164291	104393	0.12603	0.67	1.35	2553	1333	2833	1367	0.66704	0.21	0.38
280.04593@1.493	280.0459	1.49	HILIC	NEG			365809	40206	337330	52810	0.21051	-0.57	0.74	214514	37581	189825	36397	0.17829	-0.63	1.09
280.23984@10.587004	280.2398	10.59	RP	POS			318171	59306	397105	89909	0.03885	0.91	1.01	91730	17272	78096	13166	0.08541	-0.82	1.50
280.24057@10.584998	280.2406	10.58	RP	NEG	Linoleic acid (FA 18:2)	2	2852769	849988	4002198	1143309	0.02506	0.98	1.08	686396	133975	551666	123899	0.04305	-0.93	1.65
280.2412@9.666995	280.2412	9.67	RP	POS			383611	79084	417839	104629	0.43977	0.36	1.05	407095	247495	520176	381349	0.45809	0.34	0.59
280.25946@10.25	280.2595	10.25	RP	POS			1586980	251003	1502104	449352	0.61923	-0.23	0.69							
281.05963@3.5250003	281.0596	3.53	HILIC	NEG			16868	5606	22090	12273	0.24979	0.51	0.94	60535	14357	80435	21532	0.03119	0.94	1.53
281.27234@10.467003	281.2723	10.47	RP	POS	Oleamide	2	45058911	5816906	44861057	7969649	0.95217	-0.03	0.56	724909	515901	867624	433582	0.54072	0.30	0.56
281.9905@6.544999	281.9905	6.54	RP	NEG			628981	70488	645246	74851	0.64188	0.22	1.01	667751	57411	671379	52392	0.89156	0.07	0.63
282.04337@1.5090003	282.0434	1.51	HILIC	NEG										60940	11372	54351	11233	0.23613	-0.56	0.96
282.0585@1.3279997	282.0585	1.33	HILIC	NEG			148143	25399	160530	34200	0.38987	0.40	1.00							
282.06158@1.628	282.0616	1.63	HILIC	NEG										136130	22238	135596	22594	0.96049	-0.02	0.47
282.10593@1.0629998	282.1059	1.06	HILIC	NEG			102916	33901	144884	42461	0.03153	0.95	1.03	22097	9882	40281	20638	0.02669	0.96	1.55
282.25565@10.333006	282.2557	10.33	RP	NEG			71626	6647	74580	7831	0.39834	0.40	0.89	131944	8734	130968	8652	0.81583	-0.11	0.68
282.25577@10.802001	282.2558	10.80	RP	NEG	Oleic acid (FA 18:1)	1	3354533	1121149	4613492	1476756	0.05469	0.86	1.01	1724638	161062	1457633	191309	0.00483	-1.20	1.95
282.25616@10.803002	282.2562	10.80	RP	POS			186150	35937	220808	43901	0.08207	0.79	1.00	86040	12427	75324	9557	0.06316	-0.88	1.50
282.98483@5.5930004	282.9848	5.59	HILIC	NEG			15573	9065	21552	12851	0.26334	0.51	0.83							
283.09177@4.204	283.0918	4.20	HILIC	POS			27073	15862	35394	26375	0.41936	0.37	1.05							
283.16238@1.58	283.1624	1.58	HILIC	NEG										96424	39100	78953	25919	0.29632	-0.52	0.86
283.28748@10.730996	283.2875	10.73	RP	POS			3876639	957071	3757547	752406	0.77738	-0.14	0.50	40132	12365	43243	13303	0.61440	0.24	0.48
284.08148@5.5220013	284.0815	5.52	HILIC	POS			23414	15573	27721	11987	0.52924	0.31	0.99							
284.08905@3.3840013	284.0891	3.38	HILIC	NEG			64243	29403	47444	12138	0.15998	-0.72	1.20	1447135	465316	905012	437616	0.02218	-1.03	2.06
284.12067@5.522002	284.1207	5.52	HILIC	NEG			81978	24652	74347	26203	0.53392	-0.30	1.49	25725	4414	21323	4451	0.05127	-0.89	1.46
284.1207@5.5370016	284.1207	5.54	HILIC	POS			48531	16306	42190	13357	0.38865	-0.42	1.59	16355	3156	13331	3471	0.06951	-0.83	1.36
284.271@11.058005	284.2710	11.06	RP	NEG	Stearic acid (FA 18:0)	1	280823	45143	331943	73858	0.08857	0.76	0.99	174949	11613	170298	20144	0.54785	-0.27	0.74
285.0254@4.5650015	285.0254	4.57	HILIC	NEG			3220	537	3390	891	0.62311	0.22	1.22	23791	4874	22509	7076	0.65531	-0.21	0.43
286.21408@9.660003	286.2141	9.66	RP	POS			84688	7367	89248	7017	0.20045	0.61	1.21	117994	23076	112796	25172	0.65393	-0.21	0.35
286.99722@6.050002	286.9972	6.05	HILIC	NEG			40756	3101	41734	3356	0.52984	0.30	1.01	27646	3032	28999	4021	0.42658	0.37	0.80
287.2465@8.7890005	287.2465	8.79	RP	POS			375221	94725	379440	73695	0.91903	0.05	0.68	367958	286196	422861	193450	0.64983	0.23	0.37
287.24686@8.714997	287.2469	8.71	RP	POS			417487	107774	424777	80472	0.87580	0.08	0.74	403627	313191	468108	212865	0.62676	0.24	0.40
287.28308@7.5619993	287.2831	7.56	RP	POS			91139	37497	102030	55645	0.62721	0.22	0.81	153213	65913	113912	25207	0.14203	-0.76	1.50
287.28345@7.8589997	287.2835	7.86	RP	POS			341563	135640	309595	92846	0.57895	-0.28	0.88	1048993	433727	846899	171620	0.24243	-0.61	1.32
287.28372@7.6810007	287.2837	7.68	RP	POS			306152	170390	317539	142332	0.88159	0.07	0.69	530621	252636	403200	98920	0.20801	-0.65	1.36
287.9807@6.3009977	287.9807	6.30	HILIC	NEG			38180	5586	37406	3564	0.73881	-0.17	1.11	959	444	956	437	0.98827	-0.01	0.47
288.0092@1.5400003	288.0092	1.54	HILIC	NEG			77046	14510	87039	16066	0.18350	0.62	0.85							
288.05896@5.664001	288.0590	5.66	HILIC	NEG			80037	26360	99965	50065	0.29435	0.47	0.94	5806	1957	6455	2069	0.50500	0.32	0.79
288.09558@0.84000003	288.0956	0.84	HILIC	NEG			3592	732	4208	1165	0.18883	0.59	0.91	32562	5131	30969	4399	0.49606	-0.33	0.70
289.8394@3.1289992	289.8394	3.13	HILIC	POS			32807	2373	32428	3279	0.77943	-0.13	1.41	24976	1208	26516	871	0.00895	1.19	1.94
289.83997@3.1289992	289.8400	3.13	HILIC	POS			32807	2373	32428	3279	0.77943	-0.13	1.41	24976	1208	26516	871	0.00895	1.19	1.94
290.06305@2.5709996	290.0631	2.57	HILIC	POS			1209280	172379	1088115	187746	0.17185	-0.64	1.75							
290.0749@1.5190008	290.0749	1.52	HILIC	NEG			262369	87775	294510	83530	0.44132	0.37	1.07	50973	9838	54772	9296	0.41587	0.39	0.73
292.09076@6.211999	292.0908	6.21	HILIC	POS	Ethylenediaminetetraacetic acid	2								62539	63845	122684	277608	0.52029	0.28	0.53
292.1718@10.445005	292.1718	10.45	RP	POS			70075	8127	77095	10238	0.12228	0.70	0.97	17101	5329	14677	3107	0.27584	-0.55	1.32
292.20193@8.714997	292.2019	8.71	RP	POS			107659	26987	100568	19398	0.54190	-0.30	0.62	85436	63547	98576	44571	0.62808	0.24	0.41
292.20242@9.554996	292.2024	9.55	RP	POS			166862	4155	160482	9585	0.07866	-0.77	1.06	157107	7282	151078	8999	0.13362	-0.69	1.22
293.93228@4.4120016	293.9323	4.41	HILIC	POS			21177	3238	24845	6393	0.13471	0.66	0.79	59997	2535	60459	3665	0.75599	0.14	0.31
293.94037@5.600999	293.9404	5.60	HILIC	POS			9033	8882	13044	8861	0.35383	0.44	0.90							
294.05603@4.509	294.0560	4.51	HILIC	NEG			290228	42066	316326	60724	0.29708	0.48	1.01							
294.0562@5.399998	294.0562	5.40	HILIC	NEG			21889	3206	22053	3947	0.92365	0.05	0.65	115399	17281	126046	19011	0.23039	0.56	1.05
294.05695@6.1930003	294.0570	6.19	HILIC	NEG			60547	4546	58491	6489	0.44071	-0.36	1.23							
294.05936@1.4459996	294.0594	1.45	HILIC	POS			26484	12445	21838	8104	0.37798	-0.44	1.04							
294.18292@8.0529995	294.1829	8.05	RP	NEG			271494	91061	270187	79545	0.97488	-0.02	0.48	645164	402452	463084	158333	0.25522	-0.59	1.04
294.1846@13.135004	294.1846	13.14	RP	NEG	Myristyl sulfate	2	316114	193843	389307	181889	0.42489	0.38	0.83	163219	40980	157480	40181	0.76944	-0.14	0.34
294.18713@10.729003	294.1871	10.73	RP	POS			97875	10450	104954	7679	0.13125	0.73	1.03	25936	5141	24883	3077	0.61845	-0.25	0.85
294.21835@9.535003	294.2184	9.54	RP	POS			18774	10226	24644	24177	0.49891	0.30	0.99	18183	12486	20353	20911	0.78822	0.12	0.40
294.21893@9.479003	294.2189	9.48	RP	POS			41771	13977	43646	17453	0.80309	0.12	1.01	87926	32652	89437	65180	0.94983	0.03	0.37
294.21896@9.472995	294.2190	9.47	RP	NEG			15997	7123	20781	9332	0.23344	0.55	0.83	60120	15181	54347	30561	0.60902	-0.23	0.48
294.21896@9.237	294.2190	9.24	RP	NEG			105863	44687	145991	94298	0.25297	0.51	0.84	12116	6584	10932	9927	0.76536	-0.14	0.46
295.21616@8.898002	295.2162	8.90	RP	POS			166154	8144	171962	10375	0.19992	0.59	1.18	220932	25678	229685	39833	0.58018	0.25	0.66
295.25226@10.373995	295.2523	10.37	RP	POS			97332	19073	124817	28282	0.02486	0.98	1.10	13909	3332	9917	2634	0.01441	-1.12	1.86
295.98965@5.576999	295.9897	5.58	RP	NEG			890765	31297	891770	22316	0.94003	0.04	0.90	966722	49060	974263	43473	0.73771	0.16	0.65
296.23532@9.493002	296.2353	9.49	RP	NEG	9-HODE	2	812817	573329	1256314	915400	0.22639	0.55	0.88	195570	222506	239204	428014	0.78411	0.12	0.41
296.2359@9.636995	296.2359	9.64	RP	POS			82439	21038	95167	25708	0.26317	0.52	0.93	29953	8896	27355	6412	0.49886	-0.34	0.56

296.236@9.638	296.2360	9.64	RP	POS			86802	21569	97411	24627	0.34342	0.44	1.01	38617	6578	37519	7310	0.74152	-0.16	0.44
297.26752@10.588004	297.2675	10.59	RP	POS			170065	21784	196807	24670	0.02540	0.99	1.08	61080	11371	53821	9525	0.16830	-0.66	1.28
297.8896@3.1050014	297.8896	3.11	HILIC	NEG			73445	4093	73138	6066	0.89987	-0.06	1.09	57529	4633	59156	5714	0.51315	0.31	0.74
298.16@10.174997	298.1600	10.17	RP	NEG			342951	177527	488253	253961	0.17140	0.62	0.86	113193	34837	168565	169118	0.33457	0.42	0.91
298.25034@10.281004	298.2503	10.28	RP	NEG			28186	5542	31416	6723	0.27865	0.50	1.19	54287	7808	49718	15151	0.42111	-0.36	0.77
298.2509@9.647002	298.2509	9.65	RP	NEG	Ricinoleic acid	2	801630	140574	865850	192469	0.42454	0.37	0.92	413260	279393	358550	220624	0.65746	-0.22	0.61
298.2521@9.687998	298.2521	9.69	RP	POS			204653	33301	216995	25830	0.40283	0.41	0.83	91646	10749	93717	19107	0.77536	0.13	0.70
298.97983@5.294002	298.9798	5.29	HILIC	NEG			33125	3169	31441	3494	0.29873	-0.49	1.03	45171	2507	44541	5277	0.74369	-0.15	0.91
299.1932@7.579999	299.1932	7.58	RP	POS			2484	613	3288	868	0.03385	0.93	1.02	54448	9353	68117	18284	0.05740	0.83	1.64
299.19473@0.8679999	299.1947	0.87	HILIC	POS			1643	1035	2687	759	0.04419	1.01	1.27	27862	7396	37451	16852	0.12798	0.67	1.31
299.28186@10.324003	299.2819	10.32	RP	POS			136630	20345	139501	21397	0.77465	0.14	0.74	10373	1608	8720	729	0.02148	-1.13	1.88
299.28348@8.657002	299.2835	8.66	RP	POS			60483	11279	63414	16604	0.66210	0.20	0.98	21148	3231	24976	8386	0.20702	0.56	0.91
300.10248@9.820004	300.1025	9.82	RP	NEG			64614	11723	70831	9885	0.24886	0.56	0.92	22782	4707	19793	3821	0.16629	-0.66	1.16
300.26575@10.002005	300.2658	10.00	RP	NEG			118617	15669	126544	20958	0.37098	0.41	1.06	10564	2509	10431	1773	0.90087	-0.06	0.62
300.26587@9.953002	300.2659	9.95	RP	NEG			27972	3414	65868	26705	0.00113	1.38	1.98	5663	1690	7429	2020	0.05861	0.85	1.59
301.01202@6.199999	301.0120	6.20	HILIC	NEG										51166	11384	53215	22922	0.80811	0.11	0.57
301.1165@3.3919988	301.1165	3.39	HILIC	POS			5580	1720	4815	1898	0.39970	-0.41	1.84	223645	61754	139083	62521	0.01049	-1.13	2.13
302.1666@1.3029997	302.1666	1.30	HILIC	POS			47469	35990	64902	35474	0.31846	0.47	0.66							
302.2245@10.351004	302.2245	10.35	RP	NEG			164204	45372	222841	61130	0.03143	0.95	1.08	145120	38305	100084	35062	0.02013	-1.05	1.75
302.22552@9.668	302.2255	9.67	RP	POS			85796	44218	99278	75926	0.64409	0.21	0.79	163088	193321	279366	317465	0.35187	0.42	0.72
303.07297@1.3969998	303.0730	1.40	HILIC	NEG			196009	64491	197155	81670	0.97384	0.02	1.20							
303.10764@6.1499977	303.1076	6.15	HILIC	NEG			397190	138820	409618	285709	0.90539	0.05	1.07	10192	6612	11109	7400	0.78474	0.13	0.58
303.20435@1.5880005	303.2044	1.59	HILIC	POS			50024	11548	52276	17170	0.74420	0.15	0.85	10496	1655	10311	1096	0.79036	-0.13	0.29
304.05734@2.5709996	304.0573	2.57	HILIC	NEG			402693	92767	394769	119580	0.87599	-0.07	1.41							
304.12634@1.0830001	304.1263	1.08	HILIC	NEG			45290	9548	51007	11903	0.27300	0.51	0.73							
304.2378@10.794004	304.2378	10.79	RP	NEG			118243	11195	129979	12519	0.05063	0.88	1.00	80400	12480	70600	11317	0.10342	-0.76	1.33
304.2405@10.556	304.2405	10.56	RP	NEG	AA (FA 20:4)	1	1495997	384615	2057247	500851	0.01502	1.06	1.13	1382319	336537	1150778	282187	0.13959	-0.70	1.20
304.24084@10.557005	304.2408	10.56	RP	POS			193594	48341	258989	60885	0.02052	1.02	1.09	207105	43202	182174	33054	0.19841	-0.62	1.05
304.24368@9.782998	304.2437	9.78	RP	POS			13460	5833	14850	9052	0.69844	0.18	0.67	24439	43648	22537	42666	0.92714	-0.04	0.46
305.06976@1.3980002	305.0698	1.40	HILIC	NEG			66260	21337	65802	26233	0.96786	-0.02	1.22							
305.07382@4.205001	305.0738	4.21	HILIC	POS			49946	30548	58089	40798	0.63430	0.22	1.13							
305.92108@7.855001	305.9211	7.86	HILIC	NEG			58308	2915	55091	5663	0.13949	-0.66	0.82	80475	8071	81255	12375	0.87394	0.07	0.58
306.01968@1.2959998	306.0197	1.30	HILIC	NEG			706307	72475	737061	80968	0.40716	0.39	1.02							
306.03668@2.563	306.0367	2.56	HILIC	POS			260740	46502	243007	51334	0.45287	-0.35	1.64							
306.0541@2.5639997	306.0541	2.56	HILIC	NEG			142054	33512	139757	46113	0.90404	-0.06	1.41							
306.07672@1.1919999	306.0767	1.19	RP	POS			512165	149098	496162	167532	0.83289	-0.10	1.10							
306.25537@10.7109995	306.2554	10.71	RP	NEG	FA 20:3	2	208225	85588	311068	101896	0.03189	0.95	1.06	105797	25828	88666	22207	0.15664	-0.68	1.20
306.25574@10.760995	306.2557	10.76	RP	NEG			148416	47456	211112	71594	0.03956	0.90	1.05	102043	25501	83512	21065	0.11850	-0.74	1.28
307.084@6.627	307.0840	6.63	HILIC	NEG	Glutathione	2	1131454	167103	1185365	190839	0.53135	0.30	0.68	1764	505	1767	464	0.99104	0.01	0.65
307.08472@6.6340013	307.0847	6.63	HILIC	POS			1682080	242767	1881608	381434	0.19523	0.58	0.87							
307.28766@10.587999	307.2877	10.59	RP	POS			144035	31058	131436	34048	0.42355	-0.38	0.47							
309.1937@0.87999976	309.1937	0.88	HILIC	POS			30437	8899	29690	7265	0.85062	-0.09	0.99	13117	1952	13664	2253	0.58837	0.26	0.64
309.23117@9.554996	309.2312	9.55	RP	POS			2602092	108314	2577283	108237	0.63520	-0.23	0.86	2652171	50056	2647919	97948	0.90665	-0.05	0.21
309.30353@10.794004	309.3035	10.79	RP	POS			9462467	2273898	8948679	2329290	0.64338	-0.22	0.52	36863	14870	34197	18955	0.74196	-0.15	0.57
310.17786@8.0	310.1779	8.00	RP	NEG			72669	2767	71553	2689	0.40112	-0.40	0.64	76544	2718	73596	2616	0.03288	-0.97	1.77
310.17847@8.001004	310.1785	8.00	RP	POS	Biliverdin IX	1	166442	11060	175931	16056	0.15634	0.64	0.99	176199	7512	179562	8420	0.38319	0.41	0.70
310.2866@11.119997	310.2866	11.12	RP	NEG	FA 20:1	2	78300	31017	115768	41335	0.04149	0.90	1.03	49112	8394	48674	13112	0.93247	-0.04	0.71
311.31894@11.049996	311.3189	11.05	RP	POS			148064	42357	118553	43537	0.16471	-0.65	0.70	13769	4508	15546	6431	0.50063	0.31	0.78
312.1191@2.5820003	312.1191	2.58	HILIC	POS			154310	48657	154883	67130	0.98351	0.01	1.35							
312.17584@10.802001	312.1758	10.80	RP	NEG			127295	98580	143297	51473	0.68511	0.21	0.78	161169	169768	584209	1457273	0.38339	0.38	1.03
312.17593@10.731002	312.1759	10.73	RP	NEG			74547	45889	96845	51473	0.34494	0.44	0.86							
312.18967@10.4609995	312.1897	10.46	RP	NEG			45997	9228	55405	15966	0.13654	0.66	0.92	81633	18862	117275	131626	0.41665	0.36	0.86
312.2669@10.394004	312.2669	10.39	RP	POS			39788	11570	38562	8787	0.80759	-0.12	0.75	468681	112277	505633	89595	0.46030	0.36	0.58
312.2671@10.477998	312.2671	10.48	RP	POS			29702	6892	33541	11025	0.37930	0.40	0.92	3411227	381050	3301993	381795	0.55387	-0.28	0.91
312.31427@10.464998	312.3143	10.46	RP	POS			103762	4385	104446	7800	0.81726	0.11	1.05							
313.10153@1.3969996	313.1015	1.40	HILIC	NEG			141073	56006	161193	92770	0.57724	0.25	1.14	640	564	1197	978	0.15711	0.64	1.07
313.22534@0.81399965	313.2253	0.81	HILIC	POS			18918	6599	19863	6644	0.76708	0.14	0.94	21634	6034	20402	4788	0.64456	-0.23	0.54
314.11984@6.050998	314.1198	6.05	HILIC	NEG			86563	9942	92599	10599	0.23024	0.56	0.88	72011	13488	77854	16202	0.41474	0.38	0.75
314.18613@9.554996	314.1861	9.55	RP	POS			143334	3524	137651	7636	0.05421	-0.84	1.02	134016	5686	128741	8678	0.13900	-0.67	1.24
314.24527@8.942996	314.2453	8.94	RP	NEG	12,13-DiHOME	2	42454	3365	46651	9994	0.23630	0.52	0.85	15917	1874	14519	3995	0.34258	-0.42	0.75
315.31427@8.351002	315.3143	8.35	RP	POS			137286	58720	127094	39779	0.68120	-0.21	0.81	292024	141225	229771	57477	0.26821	-0.57	1.30
315.31458@8.4920025	315.3146	8.49	RP	POS			114257	35285	110685	37958	0.83892	-0.10	1.30	847121	242058	686446	87296	0.10530	-0.84	1.74
316.0883@6.292	316.0883	6.29	HILIC	NEG			58418	11625	58462	7226	0.99275	0.00	1.08							

316.1727@10.372995	316.1727	10.37	RP	POS			89480	13038	105906	16132	0.02805	0.97	1.08	8762	2697	7443	1681	0.24888	-0.58	1.16
316.18164@1.1229999	316.1816	1.12	HILIC	NEG			46589	40766	64303	47868	0.40789	0.39	0.71							
316.18246@1.1229999	316.1825	1.12	HILIC	POS			72247	59016	107154	75911	0.28689	0.49	0.71							
316.26108@9.145997	316.2611	9.15	RP	NEG			112589	16557	127776	34433	0.23812	0.53	0.98	54995	2457	53350	2991	0.21635	-0.57	1.19
316.26108@9.329996	316.2611	9.33	RP	NEG			88298	14676	94048	17389	0.45683	0.35	0.60	43769	3216	42185	2143	0.25231	-0.57	0.93
316.298@10.740998	316.2980	10.74	RP	POS			86940	21896	99281	25858	0.28757	0.50	0.67	27002	5270	27077	6940	0.97956	0.01	0.33
317.955@4.391999	317.9550	4.39	HILIC	NEG			174655	14020	182576	24859	0.40636	0.38	0.70	272672	24022	276609	25995	0.74296	0.16	0.46
318.18738@10.587004	318.1874	10.59	RP	POS			254938	24580	279202	22421	0.04561	0.92	1.04	63117	12329	57947	9768	0.34855	-0.46	1.01
318.21762@9.493998	318.2176	9.49	RP	POS			118893	75704	144990	86832	0.50486	0.31	0.92	32621	30187	36304	57723	0.86394	0.08	0.39
319.1439@1.0830001	319.1439	1.08	HILIC	NEG			28292	14442	22332	14220	0.39327	-0.41	0.63							
320.06567@3.3840013	320.0657	3.38	HILIC	NEG			5391	2349	3923	1163	0.13413	-0.76	1.21	121186	34664	78536	35251	0.01953	-1.04	2.08
320.1467@3.777	320.1467	3.78	HILIC	NEG										33533	14246	47588	19770	0.09722	0.75	1.24
320.20377@10.812997	320.2038	10.81	RP	POS			179742	21253	200288	21897	0.06015	0.86	0.94	17944	5255	15029	3443	0.19868	-0.64	1.20
320.2345@9.666995	320.2345	9.67	RP	NEG	12R-HETE	2	85819	40112	118044	78099	0.27497	0.49	0.86	146336	192410	228503	266648	0.45812	0.34	0.62
321.2673@10.557005	321.2673	10.56	RP	POS			96694	19691	123718	23122	0.01543	1.06	1.13	83388	11976	73345	10193	0.07771	-0.83	1.35
322.04575@3.4329991	322.0458	3.43	HILIC	POS			3982	1147	2896	972	0.06120	-0.92	2.50	44622	10753	30859	14410	0.03241	-0.94	1.92
322.0638@3.4329994	322.0638	3.43	HILIC	NEG										46152	12095	30358	13643	0.01820	-1.04	2.05
322.21866@11.066995	322.2187	11.07	RP	POS			55104	5931	60324	7725	0.12235	0.70	0.91	36218	4707	35022	5841	0.63604	-0.22	0.69
323.2826@10.178001	323.2826	10.18	RP	POS			71187	19005	75601	13214	0.58592	0.27	0.63	1736	562	1759	329	0.91925	0.05	0.32
323.9953@1.4830008	323.9953	1.48	HILIC	NEG			29581	7400	30981	7304	0.69337	0.19	1.05	70105	12381	66481	10548	0.51981	-0.31	0.55
324.03577@6.656999	324.0358	6.66	HILIC	NEG	Uridine monophosphate	2	378646	87650	428062	74513	0.22279	0.59	0.94							
324.2251@10.730996	324.2251	10.73	RP	NEG			52285	7007	55065	7679	0.43366	0.37	0.98	42159	5894	38421	5155	0.17690	-0.64	1.22
325.10052@6.829	325.1005	6.83	HILIC	NEG			182395	53485	185230	48402	0.90864	0.06	1.16							
325.29834@10.4349985	325.2983	10.43	RP	POS			305843	68118	311985	51269	0.83538	0.10	0.89	24633	2412	20561	3345	0.00780	-1.13	1.83
325.99347@1.4830008	325.9935	1.48	HILIC	NEG			31547	7025	32341	7672	0.82168	0.11	1.06	67586	11747	65393	9806	0.67800	-0.20	0.41
326.03894@2.562	326.0389	2.56	HILIC	NEG			83344	11214	72883	11181	0.06533	-0.85	1.98							
326.07098@8.811003	326.0710	8.81	RP	POS			77579	8664	74560	2930	0.36901	-0.47	1.40	78407	4985	77492	4471	0.69096	-0.19	0.57
326.11652@9.994997	326.1165	9.99	RP	NEG			54630	9951	62619	9165	0.09825	0.77	0.94	41793	4342	34582	5559	0.00632	-1.16	1.91
327.3141@10.700004	327.3141	10.70	RP	POS			154198	26433	162519	32567	0.55710	0.28	0.70	13297	2373	13502	2005	0.84835	0.09	0.90
327.9956@6.768	327.9956	6.77	HILIC	NEG			120272	65544	201521	185123	0.21940	0.54	0.88							
328.13345@10.342003	328.1335	10.34	RP	NEG			216882	24744	230703	13489	0.18164	0.67	0.79	60526	13502	54447	8428	0.28662	-0.53	1.10
328.24033@10.526	328.2403	10.53	RP	NEG	DHA (FA 22:6)	2	1246432	360321	1720375	361743	0.01307	1.10	1.19	2035996	361707	1910686	312153	0.44935	-0.37	0.78
328.24078@10.53	328.2408	10.53	RP	POS			183908	55012	248891	57499	0.02559	1.00	1.09	283933	41167	267559	36005	0.38868	-0.42	0.76
328.26193@10.177001	328.2619	10.18	RP	POS			88611	22709	90970	20970	0.82360	0.11	0.75	11371	4279	6395	3636	0.01881	-1.07	1.79
329.0651@6.641	329.0651	6.64	HILIC	NEG			31630	2894	30438	3414	0.43344	-0.37	0.87							
329.06595@6.6459985	329.0660	6.65	HILIC	POS			67658	3913	64197	4434	0.09604	-0.76	0.95							
329.08585@1.507	329.0859	1.51	HILIC	NEG			111412	12243	108570	12099	0.62894	-0.23	0.53	74380	13471	75074	10619	0.90684	0.06	0.49
329.10522@9.819	329.1052	9.82	RP	NEG			102331	10218	111547	8894	0.06131	0.87	1.04	63428	11018	55783	8692	0.12969	-0.73	1.23
329.25903@9.983001	329.2590	9.98	RP	POS			156756	122244	129030	95612	0.60687	-0.25	0.73	4693	3501	4138	2447	0.70978	-0.19	0.60
329.3301@8.748996	329.3301	8.75	RP	POS			61944	14819	57008	5166	0.39050	-0.45	0.77	50362	1491	50606	1658	0.74671	0.15	0.52
330.1358@10.341002	330.1358	10.34	RP	NEG			116975	12224	125610	13733	0.17596	0.63	0.97	55409	9947	48181	7283	0.10717	-0.78	1.44
330.25552@10.717004	330.2555	10.72	RP	NEG	DPA (FA 22:5)	2	349114	149982	556436	173043	0.01407	1.07	1.15	330040	65050	281634	67361	0.14056	-0.69	1.28
330.25586@10.648996	330.2559	10.65	RP	NEG			175354	56580	228359	46850	0.04968	0.92	1.04	100793	22674	74279	13369	0.01242	-1.18	1.95
330.25778@10.648996	330.2578	10.65	RP	POS			56070	16813	68366	9403	0.08927	0.84	0.99	22460	6113	13939	3835	0.00450	-1.30	2.13
330.27728@10.793	330.2773	10.79	RP	POS			541150	99565	489458	132426	0.35727	-0.43	0.75							
330.27792@10.394004	330.2779	10.39	RP	POS			83933	23015	84126	15870	0.98412	0.01	0.69	558664	142343	602943	110065	0.48059	0.35	0.57
330.28433@10.476997	330.2843	10.48	RP	POS			163056	42040	198891	70821	0.20036	0.58	0.79	17694157	1572950	17290993	2394725	0.67312	-0.19	0.76
331.2727@8.798003	331.2727	8.80	RP	POS			47084	35370	43607	30776	0.82914	-0.11	0.48	42977	35443	40712	26655	0.88281	-0.07	0.26
331.2729@8.8670025	331.2729	8.87	RP	POS			55611	41431	50655	35479	0.79170	-0.13	0.51	51463	43394	47479	32260	0.83183	-0.11	0.29
331.98712@7.270997	331.9871	7.27	RP	NEG			400206	26616	402999	26550	0.82740	0.10	0.91	443183	41515	446264	50317	0.88840	0.07	0.70
332.27084@10.813997	332.2708	10.81	RP	NEG	FA 22:4	2	147305	64424	224345	79838	0.03604	0.93	1.06	223379	48698	165927	31190	0.01252	-1.17	1.92
333.00226@6.048997	333.0023	6.05	HILIC	NEG			34715	1166	35513	2328	0.35919	0.41	1.03	26658	2757	27596	3450	0.52956	0.29	0.72
333.10336@1.0540005	333.1034	1.05	HILIC	NEG			279005	61288	315559	74678	0.26903	0.51	0.69	14150	4299	13234	4250	0.65713	-0.21	0.54
334.06558@7.62	334.0656	7.62	HILIC	NEG			297875	440181	447842	761788	0.60799	0.23	0.94							
334.10098@1.5149996	334.1010	1.51	HILIC	NEG			629648	217029	701976	198970	0.47633	0.34	1.03	97573	26532	114992	26521	0.18400	0.62	1.16
334.1448@10.372995	334.1448	10.37	RP	POS			116139	22379	143455	24237	0.02350	1.01	1.09	12299	2439	9416	2098	0.01761	-1.08	1.80
334.15976@10.588004	334.1598	10.59	RP	POS			162046	13402	175177	13184	0.05291	0.89	1.20	68742	17038	63343	13803	0.47898	-0.35	0.96
335.0807@6.181	335.0807	6.18	HILIC	POS			42253	2990	42765	2842	0.71696	0.18	0.92	10409	7347	11263	7573	0.81186	0.11	0.64
335.21432@10.730002	335.2143	10.73	RP	POS			91199	7057	98769	8966	0.06033	0.84	1.06	25838	4741	24148	2716	0.38726	-0.44	0.89
335.31906@10.8829975	335.3191	10.88	RP	POS			73678	27497	74695	24821	0.93612	0.04	0.64	182950	59051	180030	67144	0.92298	-0.05	0.37
336.0678@2.5610003	336.0678	2.56	HILIC	NEG			104706	13932	98415	16712	0.39535	-0.40	1.65							
336.16055@10.588004	336.1606	10.59	RP	POS			451649	37856	491989	40854	0.04407	0.91	1.00	154868	30511	140719	25133	0.30726	-0.50	1.08
337.09814@4.4189997	337.0981	4.42	HILIC	NEG			34347	38203	35815	61990	0.95154	0.03	0.94							

337.33475@11.109002	337.3348	11.11	RP	POS			3977992	1277253	3632941	914390	0.53029	-0.31	0.76	2847640	1295035	3587975	2014397	0.35835	0.42	0.93
338.15622@10.588004	338.1562	10.59	RP	POS			221232	22487	243290	19017	0.04213	0.94	1.04	79708	15314	72294	11967	0.27950	-0.53	1.11
338.2823@10.496001	338.2823	10.50	RP	POS			44962	9149	43712	14437	0.82588	-0.10	0.59	57335	15140	35368	21823	0.02182	-1.00	1.67
338.2829@10.567995	338.2829	10.57	RP	POS			115176	37204	140107	36230	0.17111	0.64	0.82	283083	60783	231087	64224	0.09613	-0.77	1.29
339.16302@10.445005	339.1630	10.45	RP	POS			91665	14521	103980	20137	0.14970	0.65	0.98	32001	8070	27056	4869	0.15208	-0.71	1.37
339.35022@11.438997	339.3502	11.44	RP	POS			81455	41662	67191	22230	0.39991	-0.43	0.78	57304	20063	78066	43126	0.19711	0.57	1.10
340.29813@10.803994	340.2981	10.80	RP	POS			59101	15405	69431	14688	0.16738	0.65	0.95	2651516	213074	2531247	392820	0.41993	-0.36	0.78
342.15048@10.546998	342.1505	10.55	RP	NEG			38924	8427	46778	10395	0.09359	0.76	1.03	12357	3316	9278	1746	0.03583	-1.03	1.68
342.2771@10.484995	342.2771	10.48	RP	NEG			34388	6764	39450	12854	0.29927	0.47	0.67	53243	9019	38525	13090	0.01158	-1.08	1.79
342.28824@0.93899953	342.2882	0.94	HILIC	POS			57007	78350	55300	26345	0.95418	-0.03	0.86	22393	6544	22140	11858	0.95503	-0.03	0.15
343.31012@8.970999	343.3101	8.97	RP	POS										1060860	401251	972490	335059	0.62471	-0.24	0.59
344.23502@9.657003	344.2350	9.66	RP	NEG			39347	15718	54424	38453	0.27859	0.48	0.84	77417	159250	40752	46530	0.54416	-0.32	0.92
344.29196@10.731002	344.2920	10.73	RP	NEG										76098	14439	68545	14473	0.28584	-0.51	0.91
345.00208@6.201003	345.0021	6.20	HILIC	NEG			1791	2525	2723	2382	0.43631	0.37	1.12	1802992	428621	1889300	987847	0.80722	0.11	0.51
345.0391@6.6459985	345.0391	6.65	HILIC	POS			20095	1796	19313	2487	0.44931	-0.35	0.76							
345.16077@8.701997	345.1608	8.70	RP	NEG			4212	1541	2453	1412	0.02346	-1.03	1.96	182040	38614	68988	24337	0.00001	-1.75	2.88
345.2677@10.531004	345.2677	10.53	RP	POS			324414	100436	435484	101978	0.03314	0.96	1.06	489466	58174	471687	67431	0.55595	-0.28	0.70
345.98676@6.544999	345.9868	6.54	RP	NEG			997751	127445	1022958	129647	0.68413	0.19	1.01	1120132	105245	1119855	102239	0.99559	0.00	0.62
346.00974@6.211999	346.0097	6.21	HILIC	POS										132394	38343	143949	78672	0.68911	0.18	0.56
346.2148@7.253001	346.2148	7.25	RP	POS	Corticosterone	1	56157	43173	44561	32791	0.53944	-0.30	0.77	310531	178116	257021	170169	0.52702	-0.30	0.65
347.02234@1.4169999	347.0223	1.42	HILIC	NEG			40456	19202	44904	26963	0.68805	0.19	1.22							
347.06284@6.5560007	347.0628	6.56	HILIC	NEG	Adenosine monophosphate	2	769877	179904	799872	198846	0.74129	0.16	0.77	2538	566	3861	1957	0.06446	0.81	1.35
347.06317@6.5360017	347.0632	6.54	HILIC	POS			514341	135274	592245	200795	0.34043	0.44	0.87							
347.108@10.342003	347.1080	10.34	RP	NEG			130839	18125	142433	12892	0.14921	0.70	1.06	11438	2560	9894	1734	0.16743	-0.68	1.25
347.259@10.793	347.2590	10.79	RP	POS			89977	14443	82035	19957	0.34100	-0.44	0.65							
347.2833@10.648996	347.2833	10.65	RP	POS			95229	28594	117080	17726	0.08174	0.85	0.98	27566	5793	22265	4116	0.04649	-0.95	1.60
347.28342@10.712004	347.2834	10.71	RP	POS			60483	23055	86001	18709	0.02271	1.05	1.13	30604	5368	27902	6247	0.33718	-0.45	0.97
348.00952@7.447999	348.0095	7.45	HILIC	NEG										20353	7973	23023	19369	0.69814	0.17	0.52
348.04712@6.8200006	348.0471	6.82	HILIC	NEG	Inosine monophosphate (IMP)	2	68881	26393	113451	38054	0.00914	1.11	1.47							
348.22775@10.587004	348.2278	10.59	RP	NEG			68835	8193	78575	10938	0.04466	0.89	1.05	47037	7380	38797	7395	0.03089	-0.98	1.71
349.00778@7.367001	349.0078	7.37	HILIC	NEG			477	553	810	640	0.33994	0.53	1.18	154827	36044	146974	53811	0.71625	-0.17	0.77
349.00818@7.3860006	349.0082	7.39	HILIC	POS										81435	18836	79937	35761	0.91056	-0.05	0.57
349.02048@1.4169999	349.0205	1.42	HILIC	NEG			38734	17642	44061	26730	0.61847	0.23	1.17							
349.10583@10.342003	349.1058	10.34	RP	NEG			47251	4397	51086	3426	0.06139	0.88	1.02	4328	1083	4100	603	0.60356	-0.27	0.72
350.28128@10.291006	350.2813	10.29	RP	POS			271047	13704	266272	22762	0.58931	-0.25	1.08	66348	5113	66261	4627	0.97051	-0.02	0.38
351.1715@10.445005	351.1715	10.45	RP	POS			141719	17817	159098	27317	0.12209	0.69	0.95	45845	10836	36655	7221	0.05941	-0.91	1.62
352.25888@10.476997	352.2589	10.48	RP	POS			85051	22396	102504	39449	0.25440	0.51	0.82	7108819	627532	6955070	1265644	0.74145	-0.15	0.60
353.16736@10.445005	353.1674	10.45	RP	POS			68886	8838	76638	16148	0.21363	0.56	0.81	24021	5222	20497	3746	0.13043	-0.73	1.42
353.18704@10.730002	353.1870	10.73	RP	POS			108298	6734	114357	11005	0.16898	0.62	0.95	44560	5992	41689	4568	0.28087	-0.53	1.00
354.14948@10.457	354.1495	10.46	RP	NEG			811297	95589	876750	51303	0.10717	0.80	0.87	335348	73869	273680	54881	0.06883	-0.87	1.58
354.17322@10.342997	354.1732	10.34	RP	POS			89743	9918	92751	7762	0.49295	0.34	1.25	15154	2668	14420	2725	0.57305	-0.27	0.87
354.2762@10.333006	354.2762	10.33	RP	POS			177085	51291	240394	66451	0.03497	0.93	1.11	84028	36403	44775	23273	0.02013	-1.10	1.81
354.27673@10.251005	354.2767	10.25	RP	POS			119492	36064	121112	59891	0.94427	0.03	0.68	70899	18693	37379	18381	0.00140	-1.34	2.18
354.27734@9.677999	354.2773	9.68	RP	POS										23814	8918	21891	2842	0.57154	-0.30	1.10
354.27908@10.658001	354.2791	10.66	RP	POS			60173	8701	65594	11865	0.27869	0.50	0.72							
355.12097@9.994997	355.1210	9.99	RP	NEG			135070	17236	146189	15674	0.17570	0.64	1.04	31128	9482	25528	6901	0.18303	-0.65	1.13
356.00018@6.6260004	356.0002	6.63	HILIC	NEG			25962	2615	26797	2127	0.47597	0.35	0.87							
356.08313@0.84000003	356.0831	0.84	HILIC	NEG			3833	735	3942	695	0.75477	0.15	1.12	35521	4703	32643	3288	0.16455	-0.68	1.10
356.16492@10.730002	356.1649	10.73	RP	NEG			911155	70097	971166	42348	0.05363	0.94	1.15	456193	54873	437674	41313	0.44124	-0.38	1.10
356.27097@10.770997	356.2710	10.77	RP	NEG			43356	15637	65407	20978	0.02000	1.01	1.10	39298	10805	40435	10720	0.82654	0.11	0.80
356.293@10.496001	356.2930	10.50	RP	POS	MG 18:1	1	113619	26207	116747	38829	0.84120	0.09	0.42	148731	42537	94019	56692	0.03117	-0.95	1.60
356.2932@10.567995	356.2932	10.57	RP	POS	MG 18:1	1	898153	311400	1114381	311034	0.16113	0.66	0.80	956338	233845	783900	250725	0.14999	-0.67	1.16
357.12753@1.3870002	357.1275	1.39	HILIC	NEG			100146	39150	114061	62793	0.57288	0.26	1.13	622	310	1018	959	0.24170	0.52	0.85
357.1365@10.341002	357.1365	10.34	RP	NEG			425245	27437	419926	24830	0.67553	-0.20	1.08	266507	30525	264859	29920	0.90997	-0.05	0.81
358.06226@1.5079997	358.0623	1.51	HILIC	NEG			474831	49619	459618	77372	0.61973	-0.23	0.44	213106	37782	219218	22322	0.69271	0.20	0.38
358.10483@5.167	358.1048	5.17	HILIC	POS			41194	8228	52370	10394	0.02026	1.02	1.20							
358.16898@10.730002	358.1690	10.73	RP	NEG			88260	5058	91677	4338	0.14925	0.69	0.93	49420	6159	47211	4390	0.40672	-0.41	1.14
358.3094@10.730996	358.3094	10.73	RP	POS			26636	6836	31144	4447	0.13063	0.74	0.84	826929	184772	871191	219394	0.64789	0.22	0.45
358.3162@10.803994	358.3162	10.80	RP	POS			586488	133215	657017	173328	0.34205	0.44	0.90	19057979	1717296	18501021	3664242	0.67639	-0.19	0.54
359.2674@1.0520004	359.2674	1.05	HILIC	POS			32132	6000	33700	7915	0.63850	0.22	0.79	13448	2388	12669	2149	0.48252	-0.34	0.56
360.07794@1.6310003	360.0779	1.63	HILIC	NEG										23138	3099	30578	9155	0.03222	0.93	1.51
360.08188@4.1750016	360.0819	4.18	HILIC	NEG	PDX fragment	3	2433	3443	15365	7182	0.00015	1.49	2.33	2178	1373	71517	16201	0.00000	1.90	3.22
360.0819@4.722	360.0819	4.72	HILIC	NEG	PDX fragment	3								602	640	39123	7032	0.00000	1.97	3.30

360.16116@10.556	360.1612	10.56	RP	POS			100500	13580	118935	12228	0.00853	1.17	1.27	86834	10252	81544	11587	0.31838	-0.47	0.86
360.16238@10.730002	360.1624	10.73	RP	NEG			151550	14349	167401	14460	0.03283	0.96	1.05	111603	10549	100195	11885	0.04515	-0.90	1.60
361.12256@10.446005	361.1226	10.45	RP	NEG			128998	13563	139124	7415	0.08268	0.86	1.04	13140	4058	11909	2925	0.48250	-0.35	1.01
361.13678@5.0740013	361.1368	5.07	HILIC	NEG			5049	1798	5598	2827	0.62298	0.23	1.03	305325	99973	370770	123335	0.22969	0.55	1.04
361.91953@4.4169974	361.9195	4.42	HILIC	POS			39701	5384	44400	9176	0.19381	0.58	0.79	99264	4336	102289	5608	0.21320	0.57	0.96
364.0983@6.2149973	364.0983	6.21	HILIC	POS			32004	7426	49226	26303	0.07190	0.79	1.16	2207	576	2421	951	0.56260	0.26	0.52
364.09848@6.3430014	364.0985	6.34	HILIC	POS			25881	5418	32353	9411	0.08561	0.76	1.21	7359	4828	8945	4800	0.49665	0.33	0.79
365.17953@10.587004	365.1795	10.59	RP	POS			224621	17503	241782	14118	0.03975	0.96	1.15	71551	13441	67610	12230	0.52892	-0.30	0.99
365.22458@10.455998	365.2246	10.46	RP	POS			99800	8822	94793	12755	0.33933	-0.44	0.69							
365.2702@10.363002	365.2702	10.36	RP	POS			111934	16192	114034	24455	0.82978	0.10	0.89							
365.36588@11.497994	365.3659	11.50	RP	POS			63811	17131	58281	19191	0.52717	-0.30	0.81	55622	36195	70604	40778	0.42045	0.38	0.83
366.1124@6.1999984	366.1124	6.20	HILIC	POS			5030	7452	25882	18897	0.00754	1.13	1.77	1239	1176	26998	13348	0.00011	1.58	2.75
366.1825@10.342997	366.1825	10.34	RP	POS			141473	13300	151491	10351	0.10101	0.78	0.98	23071	5138	20014	3861	0.18351	-0.65	1.11
366.25287@10.475004	366.2529	10.48	RP	NEG			12124	4309	13882	3132	0.34968	0.46	0.58	299447	54632	287279	54476	0.64417	-0.22	0.92
367.19507@10.812997	367.1951	10.81	RP	POS			197824	17293	214642	17315	0.05607	0.87	0.99	37231	10134	32041	5619	0.21907	-0.62	1.30
367.30823@10.291006	367.3082	10.29	RP	POS			423750	22979	420773	34664	0.82983	-0.10	1.11	104974	9633	107227	10132	0.63589	0.23	0.62
367.30966@10.393003	367.3097	10.39	RP	POS			48039	3306	47025	3480	0.53567	-0.29	0.70	13460	1987	13630	1750	0.85174	0.09	0.39
367.9914@6.224999	367.9914	6.22	HILIC	NEG										96195	14850	89165	17725	0.37215	-0.42	1.04
368.1124@5.258001	368.1124	5.26	HILIC	NEG										357225	118738	399933	135383	0.48550	0.33	0.78
368.1127@6.191001	368.1127	6.19	HILIC	NEG			18858	5419	18198	6953	0.82349	-0.10	1.38							
368.1658@10.638	368.1658	10.64	RP	NEG			104877	17907	123863	20389	0.05012	0.88	1.07	29931	4900	25991	4371	0.09426	-0.79	1.36
368.2501@10.475001	368.2501	10.48	RP	NEG										126756	21587	121566	18258	0.59522	-0.26	0.86
368.34412@12.449003	368.3441	12.45	RP	POS			200370	43398	182406	18957	0.30003	-0.54	0.74	879859	71147	868499	148790	0.83427	-0.09	0.64
369.17346@10.721994	369.1735	10.72	RP	NEG			58817	4628	60654	3496	0.36749	0.44	0.58	50496	3563	47323	5040	0.13597	-0.67	1.28
369.28857@8.195004	369.2886	8.20	RP	POS	Acylcarnitine 14:1	2	15308	10811	16710	15437	0.82373	0.10	1.14	128863	22007	123396	24335	0.62354	-0.23	0.73
370.18048@10.889006	370.1805	10.89	RP	NEG			42865	11416	56752	15015	0.03888	0.91	1.06	17495	3981	16076	3340	0.43214	-0.38	1.04
370.18207@10.842996	370.1821	10.84	RP	NEG			56808	9692	64822	9903	0.10211	0.76	0.94	32398	6117	30071	5548	0.41530	-0.39	1.13
370.25784@12.230004	370.2578	12.23	RP	POS			424566	67476	428530	65463	0.90160	0.06	0.26	158230	27265	164918	40130	0.67983	0.19	0.55
370.30817@10.957	370.3082	10.96	RP	POS			304296	254538	1350499	2404950	0.20104	0.56	0.92	1286228	2323448	147349	62116	0.20311	-0.69	1.13
371.09573@6.147998	371.0957	6.15	HILIC	NEG			33139	3935	31543	2432	0.33413	-0.48	1.39	2220	1207	2399	1107	0.74977	0.15	0.68
371.1519@10.545997	371.1519	10.55	RP	NEG			55532	8541	53522	4040	0.55265	-0.31	1.46	22968	7423	20671	4791	0.46181	-0.37	0.85
371.15213@10.449003	371.1521	10.45	RP	NEG			350134	53584	381436	29711	0.16491	0.70	0.83	69631	18598	59270	11940	0.19492	-0.64	1.34
371.30408@10.333006	371.3041	10.33	RP	POS			91576	26303	130182	41165	0.02724	0.96	1.09	67371	29793	36935	18790	0.02594	-1.06	1.73
371.30432@8.514005	371.3043	8.51	RP	POS	Acylcarnitine 14:0	2	189640	67983	201117	83875	0.75216	0.15	0.91	274803	64899	221760	34354	0.05958	-0.93	1.54
371.34116@9.350001	371.3412	9.35	RP	POS										1323677	511481	1220931	434069	0.65705	-0.22	0.54
372.15228@0.85099995	372.1523	0.85	HILIC	NEG										24330	5158	22422	6138	0.48289	-0.33	0.59
372.22723@10.556	372.2272	10.56	RP	NEG			49100	9943	60651	10031	0.02582	1.00	1.10	36921	6820	31736	5241	0.09708	-0.79	1.33
372.28815@9.729002	372.2882	9.73	RP	POS										58057	14416	50091	7200	0.18091	-0.68	1.53
372.28845@9.677999	372.2885	9.68	RP	POS										31526	9572	27204	3135	0.25022	-0.60	1.49
373.12363@10.445005	373.1236	10.45	RP	NEG			244104	22502	265729	22727	0.05957	0.86	1.13	46217	13348	37570	9215	0.14182	-0.72	1.32
373.31958@10.567995	373.3196	10.57	RP	POS			162331	53898	197036	53820	0.19197	0.61	0.79	369891	88564	296905	90127	0.10260	-0.76	1.29
373.888@4.1969995	373.8880	4.20	RP	POS										80436	23636	69905	25486	0.37660	-0.42	0.72
375.04343@5.3370013	375.0434	5.34	HILIC	NEG			75792	4130	79087	5856	0.17956	0.61	0.83	3085	315	3844	1834	0.22569	0.53	0.91
375.12164@10.445005	375.1216	10.45	RP	NEG			103535	9524	113613	8548	0.03276	0.98	1.16	20538	6396	16698	3716	0.15730	-0.71	1.34
375.1388@10.730996	375.1388	10.73	RP	NEG			237143	10522	241189	9539	0.41048	0.40	1.06	89901	20966	84808	18138	0.59439	-0.26	0.89
375.1389@10.730002	375.1389	10.73	RP	NEG			295083	11833	303752	12510	0.15003	0.67	1.15	115853	26940	110274	23496	0.65045	-0.22	0.87
375.3353@10.803994	375.3353	10.80	RP	POS			43329	8354	51797	13114	0.11440	0.71	1.01	2092248	168730	2034200	318190	0.62655	-0.22	0.61
376.28207@10.475004	376.2821	10.48	RP	NEG			5959	1240	6639	1307	0.27463	0.51	0.59	230268	49296	213345	25690	0.39695	-0.43	1.13
377.13696@10.730002	377.1370	10.73	RP	NEG			108280	6058	113791	5590	0.06409	0.86	1.26	43040	10292	40852	8356	0.63353	-0.23	0.89
378.0925@6.2010016	378.0925	6.20	HILIC	NEG			157984	37411	253677	154139	0.08366	0.76	1.03							
378.14886@10.372003	378.1489	10.37	RP	NEG			276136	46776	347654	52072	0.00676	1.17	1.26	76598	15089	60114	12797	0.02581	-1.02	1.77
378.2751@10.570005	378.2751	10.57	RP	POS			744182	259912	918961	249884	0.16761	0.65	0.79	329860	113126	274043	108092	0.30375	-0.49	0.99
378.2781@10.803994	378.2781	10.80	RP	POS			115614	24595	127699	26327	0.32907	0.46	0.93	1989152	251219	1948831	484490	0.82284	-0.10	0.46
379.20248@10.8099985	379.2025	10.81	RP	POS			304094	27368	342909	40948	0.02772	0.96	1.03	60537	18750	50687	8405	0.19636	-0.66	1.36
380.16537@10.5859995	380.1654	10.59	RP	NEG			1097362	124254	1234034	98804	0.02281	1.05	1.13	470248	77640	406754	62628	0.08002	-0.83	1.52
380.2912@10.803994	380.2912	10.80	RP	POS			414776	95461	454331	122524	0.45142	0.35	0.88	9067535	1111170	8821427	2194237	0.76171	-0.14	0.47
380.29214@10.730996	380.2921	10.73	RP	POS			23612	5608	29150	4758	0.04109	0.95	1.03	311468	98643	342548	124576	0.56204	0.27	0.55
381.8509@3.099999	381.8509	3.10	HILIC	NEG			52906	3328	51775	2987	0.46425	-0.35	1.41	43325	2876	45050	3988	0.30114	0.47	0.95
381.9839@7.8890047	381.9839	7.89	RP	NEG			421504	11693	422914	15078	0.82565	0.10	0.86	517956	46807	519699	45418	0.93756	0.04	0.64
382.0339@6.057999	382.0339	6.06	HILIC	NEG			32082	2625	33738	3111	0.23638	0.55	0.87	25313	1577	26844	1445	0.04922	0.91	1.48
382.0732@2.5639997	382.0732	2.56	HILIC	NEG			83481	25008	87656	50200	0.82133	0.10	1.23							
382.10916@6.208	382.1092	6.21	HILIC	POS			86197	23332	108115	50565	0.24231	0.52	0.95							
382.13348@9.884002	382.1335	9.88	RP	POS			92464	8075	89949	3991	0.43740	-0.40	1.00	51786	6109	55326	5526	0.22055	0.58	1.03

382.18057@10.794004	382.1806	10.79	RP	NEG			1144479	98937	1231252	69188	0.05430	0.92	1.05	721722	97088	668261	70821	0.21285	-0.61	1.35
382.2037@10.729003	382.2037	10.73	RP	POS			188599	19561	198967	11710	0.21010	0.63	0.96	79215	13528	72019	7485	0.20272	-0.64	1.23
382.39276@11.109002	382.3928	11.11	RP	POS			66843	10604	61601	13415	0.36674	-0.42	0.93	38150	15294	45890	20305	0.36872	0.42	0.94
383.15195@10.454996	383.1520	10.45	RP	NEG			759909	37755	775319	27085	0.34768	0.46	0.97	323409	55601	287875	35946	0.14149	-0.72	1.37
384.12024@6.2799993	384.1202	6.28	HILIC	POS			28786	5495	31786	9916	0.42776	0.36	1.10							
384.1293@5.0709996	384.1293	5.07	HILIC	NEG			2272	817	2446	1174	0.71570	0.17	1.03	24656	10862	30075	13186	0.35176	0.43	0.97
384.15997@10.53	384.1600	10.53	RP	POS			35109	10203	46232	8604	0.02586	1.02	1.10	41095	5514	39767	6878	0.65492	-0.21	0.73
384.17957@10.454996	384.1796	10.45	RP	NEG			79354	22123	82596	17783	0.74111	0.16	0.77	11664	4205	9701	3592	0.30976	-0.49	1.12
384.1961@11.058005	384.1961	11.06	RP	NEG			452782	52462	523351	71598	0.02690	0.97	1.06	371040	46239	364466	54023	0.78418	-0.13	0.85
384.3392@11.399001	384.3392	11.40	RP	POS										85228	74076	101365	123982	0.73642	0.15	0.45
384.33954@10.946006	384.3395	10.95	RP	POS			6767	1456	7025	2283	0.77400	0.13	0.66	201039	162545	220844	225370	0.83119	0.10	0.45
385.1677@10.747003	385.1677	10.75	RP	NEG			727320	34353	722170	32770	0.75101	-0.15	1.03	633789	94656	632468	77623	0.97500	-0.02	0.76
385.28284@0.94200003	385.2828	0.94	HILIC	POS			23654	6596	21908	3639	0.51487	-0.33	1.16	32079	5286	29532	5458	0.33052	-0.46	0.80
385.94205@4.383	385.9421	4.38	HILIC	NEG			137912	8292	141582	14173	0.50256	0.31	0.66	186096	13663	189300	20812	0.69953	0.18	0.48
386.17575@7.7130003	386.1758	7.71	RP	POS			176749	94848	173444	70153	0.93561	-0.04	0.64	354498	180821	302155	121809	0.49508	-0.34	0.56
386.19525@10.731002	386.1953	10.73	RP	NEG			64321	11957	65640	10603	0.81001	0.12	0.67							
386.20056@11.058005	386.2006	11.06	RP	NEG			49022	5306	56017	6840	0.02512	0.98	1.06	38885	5118	38262	7592	0.83815	-0.09	0.74
386.34113@11.119997	386.3411	11.12	RP	POS			12214	3884	9406	1691	0.10633	-0.89	1.46	264925	65309	237803	72188	0.41465	-0.38	0.91
387.13788@10.639003	387.1379	10.64	RP	NEG			31835	7799	39779	9208	0.06315	0.84	1.08							
388.1929@11.058005	388.1929	11.06	RP	NEG			58456	3084	61563	2745	0.04032	0.94	1.28	46832	3322	46868	4247	0.98439	0.01	0.67
388.26126@12.216002	388.2613	12.22	RP	POS			69482	24041	85534	13110	0.11595	0.78	1.06	228815	49332	241895	77338	0.66865	0.20	0.44
389.15405@10.832	389.1541	10.83	RP	NEG			151756	15149	159048	11002	0.27251	0.54	1.11	13489	5346	11240	2500	0.29620	-0.54	1.31
389.1558@6.158002	389.1558	6.16	HILIC	NEG			34339	3635	33422	2657	0.55966	-0.29	1.50	1836	987	1876	1447	0.94445	0.03	0.57
389.25964@9.391002	389.2596	9.39	RP	NEG			201358	49187	160211	22024	0.05139	-0.98	2.11							
390.2775@10.925997	390.2775	10.93	RP	POS	Dioctyl phthalate	1	548164	224746	456449	25971	0.28342	-0.58	0.80	362937	69159	380454	86298	0.63836	0.22	0.70
391.2858@10.476004	391.2858	10.48	RP	POS			386906	31349	384425	47914	0.89637	-0.06	0.83	10049	4622	12110	3977	0.33256	0.47	0.88
392.14957@10.556	392.1496	10.56	RP	NEG			75194	5703	79952	5095	0.08366	0.81	1.26	83299	7601	73911	7448	0.01766	-1.06	1.72
392.26886@10.484995	392.2689	10.48	RP	NEG			37486	14410	47574	21727	0.25405	0.52	0.69	47577	14009	29296	17889	0.02572	-0.98	1.65
392.2907@9.605997	392.2907	9.61	RP	NEG	Deoxycholic acid	2	7717	3882	26988	12735	0.00066	1.41	1.86	41738	23065	189730	126656	0.00413	1.23	2.10
393.1266@1.0830001	393.1266	1.08	HILIC	NEG			45817	15783	53858	17517	0.32021	0.47	0.93							
394.21356@10.727998	394.2136	10.73	RP	POS			298375	23587	320483	21441	0.05600	0.88	1.00	123365	18007	113328	12539	0.20252	-0.63	1.22
394.24976@10.466999	394.2498	10.47	RP	NEG										80341	23460	76180	21588	0.70347	-0.18	0.49
394.26944@10.658001	394.2694	10.66	RP	POS			53642	10536	54054	13369	0.94257	0.03	0.50							
394.28467@10.730996	394.2847	10.73	RP	NEG			2286	559	2870	843	0.09565	0.74	1.34	67310	14984	65612	19196	0.83542	-0.10	0.22
394.28482@10.803002	394.2848	10.80	RP	NEG			15982	3647	15533	2686	0.77549	-0.14	0.56	602703	56215	573156	110084	0.47205	-0.32	0.69
394.3075@10.311003	394.3075	10.31	RP	POS			303590	8411	304364	11850	0.87330	0.07	0.88	87095	10188	87802	9842	0.88378	0.07	0.39
395.12845@10.342003	395.1285	10.34	RP	NEG			43604	7217	48158	5354	0.15854	0.68	0.95	4112	1554	4296	1340	0.79476	0.13	0.53
395.98322@7.270997	395.9832	7.27	RP	NEG			907574	72975	916600	78866	0.80430	0.12	0.96	1075628	113663	1076244	113373	0.99100	0.01	0.65
396.2097@10.727998	396.2097	10.73	RP	POS			149587	12298	156115	13614	0.30032	0.49	1.12	62544	8922	59122	6674	0.38209	-0.43	1.06
396.2272@10.526	396.2272	10.53	RP	NEG			54458	11821	69950	12140	0.01408	1.09	1.16	65013	8176	62494	5940	0.47687	-0.35	0.87
396.23682@10.802001	396.2368	10.80	RP	NEG			57367	6766	62983	4448	0.06370	0.90	1.18	24871	4664	20727	4724	0.07968	-0.81	1.41
396.2824@10.802998	396.2824	10.80	RP	NEG										197662	20241	184976	35344	0.35296	-0.42	0.87
397.1214@10.372995	397.1214	10.37	RP	NEG			108491	24406	142438	26971	0.01201	1.10	1.21	9669	2208	7652	1671	0.04977	-0.93	1.55
397.16776@10.587004	397.1678	10.59	RP	NEG			443434	56658	497219	48541	0.04914	0.91	1.06	175400	39810	151937	26228	0.17414	-0.67	1.31
397.20425@10.795997	397.2043	10.80	RP	NEG			65531	4275	70627	7859	0.09888	0.73	0.88	72830	5487	69577	8011	0.32165	-0.45	0.94
397.22583@9.881998	397.2258	9.88	RP	POS			329289	235962	344572	137701	0.87364	0.08	1.01	81878	37686	85367	31093	0.83585	0.10	0.58
397.31995@8.687001	397.3200	8.69	RP	POS	Acylcarnitine 16:1	2	79935	35104	85794	62371	0.80463	0.11	1.10	289803	62462	228896	34713	0.02972	-1.05	1.74
398.31815@10.350001	398.3182	10.35	RP	POS			55163	27823	48573	31918	0.64573	-0.22	0.75							
399.12143@10.372995	399.1214	10.37	RP	NEG			41082	9636	54414	11104	0.01395	1.08	1.16	2914	603	2140	469	0.00948	-1.18	1.92
399.13892@10.587004	399.1389	10.59	RP	NEG			351530	24610	373736	24224	0.07237	0.83	1.12	142674	39507	127605	35363	0.41192	-0.40	0.93
399.1828@10.803002	399.1828	10.80	RP	NEG			488414	50446	528712	41427	0.08811	0.81	1.07	138092	27968	124301	16132	0.23860	-0.59	1.38
399.1832@10.885003	399.1832	10.89	RP	NEG			90596	14233	112440	23249	0.02537	0.97	1.09	49032	9450	47763	6904	0.75522	-0.16	1.05
399.29877@9.822999	399.2988	9.82	RP	POS			59794	2983	59932	3278	0.92663	0.04	0.75	61308	2993	61618	3045	0.83063	0.10	0.61
399.29883@9.627998	399.2988	9.63	RP	POS			65862	3163	67876	3350	0.20809	0.59	1.01	67898	1762	69299	3540	0.29096	0.47	1.07
399.33582@9.023002	399.3358	9.02	RP	POS	Acylcarnitine 16:0	1	536139	153899	580324	213140	0.61625	0.23	1.00	1102867	254193	921082	135439	0.09375	-0.84	1.37
400.3347@10.927001	400.3347	10.93	RP	POS			326718	59484	274486	76061	0.11947	-0.71	1.76	280021	106731	269983	132970	0.86096	-0.08	0.16
400.33554@11.100004	400.3355	11.10	RP	POS			15715	2768	12781	2210	0.02747	-1.02	1.94	819763	662668	918564	586691	0.74521	0.16	0.74
401.13724@10.583006	401.1372	10.58	RP	NEG			152075	12475	161939	11520	0.10333	0.76	1.20	58545	16370	51227	15372	0.34679	-0.45	0.97
401.1555@10.782996	401.1555	10.78	RP	NEG			274517	16703	288842	16127	0.08406	0.80	1.05	53918	18025	45975	9437	0.28209	-0.55	1.20
401.16397@10.727998	401.1640	10.73	RP	NEG			101021	6846	102500	6715	0.65195	0.22	1.05	47404	11164	43204	7631	0.37977	-0.44	1.01
402.15616@10.454003	402.1562	10.45	RP	NEG			183852	25444	206137	15591	0.04952	0.95	1.02	103674	19092	85656	16718	0.05179	-0.90	1.64
402.21902@10.450997	402.2190	10.45	RP	NEG			73466	11952	73543	7797	0.98769	0.01	0.58	23089	5591	19676	3985	0.16754	-0.67	1.42
402.2259@9.6900015	402.2259	9.69	RP	POS			94945	65720	74023	35988	0.43478	-0.40	1.04	59438	3986	60473	1570	0.50464	0.35	0.84

402.38306@6.936002	402.3831	6.94	RP	POS			92891	2214	94863	3255	0.14521	0.66	1.76	73231	2483	73093	2034	0.90097	-0.06	0.29
403.13565@1.4190001	403.1357	1.42	HILIC	POS			51648	15458	51834	19359	0.98212	0.01	1.29							
403.16992@11.058005	403.1699	11.06	RP	NEG			116421	17014	137469	20488	0.02855	0.97	1.09	102223	18854	99869	24301	0.81969	-0.11	0.76
403.20206@7.7130003	403.2021	7.71	RP	POS			81111	43277	80828	34794	0.98821	-0.01	0.63	183074	85383	157877	64187	0.49947	-0.33	0.54
404.16467@10.556	404.1647	10.56	RP	NEG			137211	22921	166748	21677	0.01291	1.11	1.18	183665	26912	158046	25079	0.05459	-0.89	1.44
404.17065@10.730002	404.1707	10.73	RP	NEG			234135	10925	236828	14043	0.65272	0.21	1.17	160108	19747	147480	13834	0.14793	-0.71	1.35
404.20215@6.198999	404.2022	6.20	HILIC	POS			18943	4465	21976	5218	0.20078	0.59	1.06							
404.31335@10.802001	404.3134	10.80	RP	NEG			7008	1433	7335	1667	0.66067	0.21	0.54	294399	51189	262793	34536	0.15794	-0.69	1.41
405.15714@10.342003	405.1571	10.34	RP	NEG			94660	14933	104980	9408	0.11260	0.78	0.98	19884	4569	17335	3102	0.19952	-0.63	1.17
405.16837@11.058005	405.1684	11.06	RP	NEG			50315	6883	57502	9434	0.07846	0.79	1.05	41482	8092	42010	10520	0.90544	0.06	0.77
407.15182@10.372995	407.1518	10.37	RP	NEG			355992	37297	412706	40692	0.00659	1.17	1.28	142839	22843	112447	21820	0.01092	-1.13	1.90
408.17786@10.373	408.1779	10.37	RP	NEG			28489	18673	39844	18993	0.22014	0.58	0.82							
408.2862@9.011996	408.2862	9.01	RP	NEG			14622	12858	317800	338266	0.01774	1.04	1.46	17728	22085	131742	175333	0.06801	0.80	1.39
408.28644@8.2560005	408.2864	8.26	RP	NEG			13514	7434	308516	288905	0.00904	1.14	1.44	19077	8621	448241	464503	0.01526	1.06	1.78
408.2865@8.338002	408.2865	8.34	RP	NEG			14709	7392	127330	118683	0.01336	1.08	1.43	46131	24776	228655	229078	0.03076	0.94	1.60
409.168@10.601997	409.1680	10.60	RP	NEG			641613	56474	636357	59680	0.85051	-0.09	1.07	574750	99473	555219	90735	0.67259	-0.21	0.81
410.19315@10.587001	410.1932	10.59	RP	NEG			131699	15076	139660	13194	0.25678	0.54	0.71							
410.21304@11.119997	410.2130	11.12	RP	NEG			59729	22879	84325	27697	0.05386	0.87	1.06	51302	5885	50685	15361	0.90872	-0.05	0.56
410.23654@11.062003	410.2365	11.06	RP	POS			82380	9759	91670	8562	0.05013	0.91	1.05	75711	13990	74112	15568	0.82139	-0.11	0.60
410.3177@11.087004	410.3177	11.09	RP	NEG	3,3',5,5'-Tetra-tert-butyl-4,4'-dihydroxybiphenyl	2	152463	13161	138829	9592	0.02759	-1.03	1.43	195123	31344	198432	56507	0.87689	0.07	0.48
411.1153@10.445005	411.1153	10.45	RP	NEG			71540	8501	77101	6881	0.15437	0.68	0.96	4194	1822	3153	894	0.16690	-0.70	1.28
411.1832@10.833006	411.1832	10.83	RP	NEG			505570	26561	506412	24292	0.94552	0.03	1.24	248081	54749	220845	32561	0.23716	-0.59	1.38
411.18353@10.782004	411.1835	10.78	RP	NEG			217194	19140	207630	22403	0.34198	-0.44	0.79	113046	24156	102010	12591	0.26538	-0.57	1.27
411.33502@10.311003	411.3350	10.31	RP	POS			1022807	40435	1017698	47656	0.80851	-0.11	0.98	331492	49750	334321	41621	0.89919	0.06	0.47
411.33575@10.411003	411.3358	10.41	RP	POS			74102	4622	71542	5775	0.31004	-0.47	0.97	35058	3300	35215	5174	0.93877	0.04	0.49
412.21066@10.802001	412.2107	10.80	RP	NEG			153913	23754	159842	17375	0.56388	0.29	0.72	49178	8374	42839	9503	0.15041	-0.66	1.19
412.21683@11.119997	412.2168	11.12	RP	NEG			43761	15332	62713	19301	0.03218	0.95	1.05	37539	5573	36548	10290	0.79787	-0.12	0.75
413.19894@10.475001	413.1989	10.48	RP	NEG										389867	49196	363231	38679	0.22957	-0.58	1.00
413.199@11.058005	413.1990	11.06	RP	NEG			548263	19977	575742	30318	0.03356	0.93	1.06	608217	71789	603069	75511	0.88426	-0.07	0.78
413.31442@0.88999975	413.3144	0.89	HILIC	POS			22913	5074	20578	3660	0.29242	-0.52	1.44	37327	7060	34846	7630	0.48420	-0.33	0.61
414.224@11.058	414.2240	11.06	RP	NEG			56950	13373	62045	17168	0.48818	0.32	0.88							
414.2551@10.800997	414.2551	10.80	RP	NEG			119024	13377	123152	11949	0.50492	0.32	1.31	75184	11782	65991	10279	0.10110	-0.77	1.44
414.37085@11.509002	414.3709	11.51	RP	POS										64169	15907	62297	19328	0.82416	-0.10	0.51
415.14142@10.342003	415.1414	10.34	RP	NEG			51588	6489	56524	6730	0.13270	0.70	1.02	36334	5261	33594	3499	0.22706	-0.60	1.15
415.33215@0.87999976	415.3322	0.88	HILIC	POS			28294	5979	29970	6715	0.58285	0.26	1.01	33772	10526	32238	8220	0.74016	-0.16	0.50
416.3291@10.0079975	416.3291	10.01	RP	POS			91971	22565	103864	17060	0.23643	0.58	0.70	2264	934	2035	1160	0.64908	-0.21	0.57
418.30923@11.256996	418.3092	11.26	RP	POS			3812566	94699	3846255	93706	0.46144	0.35	1.14	2824335	596996	2912097	664684	0.77170	0.14	0.58
418.34457@14.001002	418.3446	14.00	RP	POS			411262	31261	413845	20753	0.84379	0.10	0.59	280354	27704	287636	24852	0.56996	0.28	0.65
419.3162@10.791997	419.3162	10.79	RP	POS			73621	12981	65657	19177	0.30920	-0.46	0.90							
420.24637@10.467003	420.2464	10.47	RP	NEG			9919	2704	10454	2466	0.67056	0.21	0.45	151633	15080	144443	17137	0.35693	-0.43	0.77
421.2936@10.802001	421.2936	10.80	RP	NEG										52376	7775	48173	5422	0.21580	-0.61	1.46
422.01193@6.708	422.0119	6.71	HILIC	NEG			153896	59088	192281	74960	0.24011	0.54	0.73							
422.23267@11.919004	422.2327	11.92	RP	NEG			174055	86950	179504	103147	0.90458	0.06	0.52	29387	7064	32776	6149	0.30027	0.50	0.87
422.2448@11.062003	422.2448	11.06	RP	POS			133698	14515	148984	20313	0.07964	0.78	1.00	113678	16216	110611	20228	0.72518	-0.16	0.69
423.13818@10.557005	423.1382	10.56	RP	NEG			72400	14984	87046	14364	0.05125	0.90	1.04	68622	10762	62411	12502	0.27259	-0.51	0.98
423.1414@10.446005	423.1414	10.45	RP	NEG			58375	7707	60641	5324	0.49113	0.34	1.03	7395	2159	5794	1426	0.09355	-0.82	1.38
423.15924@10.730002	423.1592	10.73	RP	NEG			103265	10355	103181	8759	0.98575	-0.01	1.03	34763	9286	34548	9457	0.96190	-0.02	0.67
423.33545@8.880003	423.3355	8.88	RP	POS	Acylcarnitine 18:2	2	55480	22993	61818	42146	0.69010	0.18	1.06	233529	40740	214917	40442	0.34740	-0.45	0.89
424.26184@10.424996	424.2618	10.42	RP	POS			77706	3814	77689	1028	0.99038	-0.01	0.71	34257	3074	34384	3653	0.93714	0.04	0.31
425.1984@10.955996	425.1984	10.96	RP	NEG			36980	12591	51250	15826	0.04719	0.89	1.02	20784	3898	18500	4793	0.27953	-0.50	1.31
425.35135@9.139005	425.3514	9.14	RP	POS	Acylcarnitine 18:1	2	288030	121351	310103	217677	0.78896	0.12	1.13	1201592	214341	1040722	162281	0.09978	-0.79	1.30
427.3672@9.411997	427.3672	9.41	RP	POS	Acylcarnitine 18:0	1	117241	46251	134674	76103	0.55703	0.27	1.22	236284	34832	247119	45180	0.57279	0.26	0.49
428.16458@10.526	428.1646	10.53	RP	NEG			100075	30660	133995	25317	0.02333	1.04	1.15	183700	28395	174097	26391	0.47227	-0.35	0.76
428.17035@10.587004	428.1704	10.59	RP	NEG			204359	12662	214234	8235	0.07868	0.85	0.93	134858	20183	120367	17615	0.12902	-0.72	1.50
428.3658@11.640007	428.3658	11.64	RP	POS			125275	22309	167423	31714	0.00402	1.21	1.82	876457	328396	957782	380808	0.63275	0.23	0.49
429.19727@11.058005	429.1973	11.06	RP	NEG			137629	13757	155246	17871	0.02962	0.96	1.08	131711	24779	128144	27079	0.77434	-0.14	0.75
429.90662@4.4219985	429.9066	4.42	HILIC	POS			45009	4258	50132	8105	0.10434	0.72	0.87	96481	4229	98631	4935	0.33304	0.45	0.79
430.16415@10.587004	430.1642	10.59	RP	NEG			53091	7073	57708	5869	0.15832	0.68	1.13	27143	4603	29492	5850	0.35300	0.43	1.04
430.17813@10.648003	430.1781	10.65	RP	NEG			40659	12188	48844	7910	0.12422	0.75	0.95	30632	8063	21402	3195	0.01220	-1.23	2.04
430.1865@10.793	430.1865	10.79	RP	NEG			404790	25795	428196	25967	0.07335	0.82	1.06	259301	36489	237431	22495	0.16211	-0.69	1.46
430.2009@10.475004	430.2009	10.48	RP	NEG			7294	1848	7711	1542	0.61633	0.25	0.34	343891	38218	337876	34231	0.73275	-0.17	0.66
430.41418@7.6309996	430.4142	7.63	RP	POS			123167	8613	130013	14512	0.23101	0.54	1.16	121040	6405	121671	5449	0.82741	0.11	0.73
431.1726@10.449003	431.1726	10.45	RP	NEG			288973	48497	319226	26907	0.14031	0.74	0.85	54530	15753	42618	11498	0.09463	-0.80	1.49

431.98038@8.421	431.9804	8.42	RP	NEG			152146	11790	149781	12592	0.68657	-0.19	1.02	97197	5785	95509	4721	0.51480	-0.32	0.80
432.2016@11.058997	432.2016	11.06	RP	NEG			195495	16831	201571	22633	0.52218	0.30	1.19	143390	12094	145111	14576	0.78743	0.13	0.73
432.28796@10.813002	432.2880	10.81	RP	POS			79614	13757	75267	11842	0.48914	-0.34	0.79							
433.06613@6.050002	433.0661	6.05	HILIC	NEG			215159	24543	228497	29806	0.31160	0.47	0.99	134562	15673	144753	21897	0.26572	0.51	0.91
433.1679@10.556	433.1679	10.56	RP	NEG			98681	10179	104399	12389	0.29672	0.48	0.95	172614	20617	158062	25986	0.20184	-0.59	1.11
433.1853@10.727998	433.1853	10.73	RP	NEG			286808	26539	301739	22656	0.22434	0.58	1.12	116694	25750	106472	15346	0.34074	-0.48	1.10
434.1942@10.556	434.1942	10.56	RP	NEG			40892	7037	47386	4853	0.04375	0.96	1.05	18596	8385	14780	7752	0.33547	-0.46	1.11
435.18378@10.700004	435.1838	10.70	RP	NEG			54967	7429	66799	7345	0.00358	1.25	1.45	47927	8112	46819	6406	0.75674	-0.15	0.51
435.8793@7.8599977	435.8793	7.86	HILIC	NEG			45927	3144	44062	4279	0.30105	-0.48	1.07	63542	4887	66162	7161	0.37010	0.41	0.79
437.13138@10.579996	437.1314	10.58	RP	NEG			130427	15755	141316	13422	0.13984	0.70	1.07	23282	7938	20498	8077	0.47302	-0.34	0.84
437.19766@10.894004	437.1977	10.89	RP	NEG			75570	23881	104556	25873	0.02420	1.00	1.10	50477	7279	48219	9595	0.57733	-0.26	0.94
437.291@10.362	437.2910	10.36	RP	POS			150890	31065	171074	44948	0.27622	0.50	1.04	37920	8597	38479	7203	0.88505	0.07	0.45
437.2999@10.355002	437.2999	10.36	RP	NEG			284663	50207	335101	90250	0.15266	0.64	1.00	121509	35686	121536	32859	0.99872	0.00	0.42
438.09778@4.7129993	438.0978	4.71	HILIC	NEG										594	720	23925	4798	0.00000	1.95	3.26
438.19635@10.536996	438.1964	10.54	RP	POS			106383	4544	106371	5684	0.99606	0.00	0.97	68699	8799	68163	12588	0.91668	-0.05	0.53
438.33374@10.322999	438.3337	10.32	RP	POS			126955	8731	121947	7005	0.20705	-0.61	1.22	59038	5145	59673	3741	0.77409	0.14	0.54
438.34888@11.213004	438.3489	11.21	RP	NEG			169900	122688	182976	117347	0.82158	0.11	0.53	138089	133274	127098	93311	0.84618	-0.10	0.20
439.14618@10.812003	439.1462	10.81	RP	NEG			89205	11279	93757	8361	0.35713	0.45	1.02	2684	1537	2106	793	0.35445	-0.47	1.17
439.2113@11.119997	439.2113	11.12	RP	NEG			113472	26956	151119	37003	0.02265	1.00	1.09	114765	20125	115710	26077	0.93185	0.04	0.98
440.3259@2.0260007	440.3259	2.03	HILIC	POS			10727	6629	9248	7910	0.69415	-0.20	1.04	31444	11662	33959	13397	0.67562	0.20	0.50
440.3264@1.822999	440.3264	1.82	HILIC	POS			32942	29002	42329	29607	0.53085	0.32	1.53	60127	23510	63853	25394	0.75096	0.15	0.52
441.15704@10.446005	441.1570	10.45	RP	NEG			94395	10657	109365	14342	0.02062	1.01	1.14	57091	10881	49636	7463	0.12147	-0.75	1.43
441.16162@11.058005	441.1616	11.06	RP	NEG			41046	9125	49164	9293	0.07997	0.81	1.06	28762	9023	29235	9946	0.91703	0.05	0.78
441.2298@10.731002	441.2298	10.73	RP	NEG										84031	12282	80899	15206	0.63460	-0.22	0.42
441.23026@10.802998	441.2303	10.80	RP	NEG										347680	32928	334948	47632	0.51229	-0.30	0.75
441.3456@0.84000003	441.3456	0.84	HILIC	POS			23978	5116	24181	5917	0.93870	0.04	1.30	44357	11405	42083	11804	0.68404	-0.19	0.62
441.98505@6.554	441.9851	6.55	HILIC	NEG			27222	5617	28359	5623	0.67510	0.20	0.70							
443.16034@11.058005	443.1603	11.06	RP	NEG			30593	6653	36720	7151	0.07707	0.81	1.02	21264	5516	22136	7135	0.77333	0.13	0.83
443.17285@10.730002	443.1729	10.73	RP	NEG			190016	13435	202267	17314	0.10826	0.73	1.12	182677	19707	170261	14870	0.16099	-0.68	1.29
443.36093@0.8379998	443.3609	0.84	HILIC	POS			11609	3097	15541	5615	0.07758	0.78	0.95	21419	3229	29690	7278	0.00597	1.16	1.95
444.36063@11.671998	444.3606	11.67	RP	POS			4950	1815	4902	1649	0.95382	-0.03	0.73	74359	24025	67524	20326	0.53039	-0.31	0.68
444.36078@11.334998	444.3608	11.33	RP	POS			5484	997	5685	911	0.66488	0.21	1.00	297006	159939	399099	242040	0.29799	0.47	0.77
444.36084@11.725004	444.3608	11.73	RP	POS			7485	1456	6520	1344	0.16708	-0.65	1.02	96848	32152	92539	30080	0.77489	-0.14	0.61
444.36118@11.263	444.3612	11.26	RP	POS										70839	44747	90432	56614	0.42291	0.37	0.64
445.03632@6.711999	445.0363	6.71	HILIC	NEG			73126	22654	79255	23593	0.58265	0.26	0.68							
445.14224@10.371001	445.1422	10.37	RP	NEG			39247	9786	51798	9761	0.01489	1.08	1.18	4039	979	3108	774	0.04419	-0.94	1.59
445.1516@10.449003	445.1516	10.45	RP	NEG			54900	8987	61701	4339	0.07528	0.89	1.07	18318	4341	15397	3342	0.13816	-0.71	1.34
445.98044@7.8890047	445.9804	7.89	RP	NEG			963187	30638	961356	20108	0.88631	-0.07	0.83	1172521	112796	1180988	114862	0.87708	0.07	0.64
446.02417@6.8189993	446.0242	6.82	HILIC	NEG			22948	9009	45259	19226	0.00544	1.17	1.38							
446.2071@1.043	446.2071	1.04	HILIC	NEG			27475	10666	28232	16917	0.90926	0.05	0.64							
446.3402@11.546998	446.3402	11.55	RP	POS			277769	10556	270696	9217	0.15475	-0.68	1.29	264550	58077	255123	56328	0.73300	-0.16	0.58
447.13885@10.526	447.1389	10.53	RP	NEG			47146	16484	68231	16100	0.01443	1.09	1.16	52212	9619	51868	11921	0.94675	-0.03	0.62
447.15915@10.587004	447.1592	10.59	RP	NEG			154478	15022	160046	10255	0.38651	0.43	0.89	55089	14935	49753	13213	0.43955	-0.37	0.94
447.33508@8.891	447.3351	8.89	RP	POS	Acylcarnitine 20:4	2	49259	35382	59176	80099	0.73064	0.15	1.10	73005	13332	77910	13269	0.44759	0.36	0.80
447.348@11.109002	447.3480	11.11	RP	POS			47566	14957	44463	10874	0.63029	-0.24	0.71	40026	15425	47851	24543	0.42077	0.37	0.78
448.2748@10.793	448.2748	10.79	RP	NEG										111563	12786	106308	14532	0.42574	-0.37	0.67
449.17508@10.813002	449.1751	10.81	RP	NEG			144979	13215	147451	11678	0.68346	0.20	0.97							
449.35077@10.925997	449.3508	10.93	RP	POS			86496	30097	73265	6032	0.25375	-0.61	0.81	57262	12709	59238	16142	0.77468	0.13	0.60
450.33325@11.059002	450.3333	11.06	RP	POS			50427	26802	45352	16253	0.64589	-0.23	0.88							
451.1903@11.058005	451.1903	11.06	RP	NEG			49369	10526	63135	11862	0.01801	1.04	1.12	44281	12474	40258	10572	0.47799	-0.35	0.87
451.26974@9.841002	451.2697	9.84	RP	NEG	LysoPE 16:1	2	58051	7661	63249	12198	0.28539	0.48	0.73	102523	16291	95079	10236	0.28051	-0.54	0.96
451.27023@9.841002	451.2702	9.84	RP	POS			46690	8611	56157	11242	0.05823	0.85	0.93	86219	12738	79233	8718	0.20766	-0.62	1.05
452.0928@6.412001	452.0928	6.41	HILIC	NEG			53258	10785	49398	6835	0.39458	-0.43	0.99							
452.09326@6.4140005	452.0933	6.41	HILIC	POS			12786	3596	10817	1945	0.18932	-0.66	1.28							
452.29684@10.802001	452.2968	10.80	RP	NEG			38275	7283	43525	7737	0.15710	0.66	0.99	24185	4729	17711	3694	0.00634	-1.22	1.98
453.28464@1.8320005	453.2846	1.83	HILIC	NEG			8875	2648	12018	4240	0.07151	0.80	0.97	48083	7630	47136	6561	0.78425	-0.13	0.72
453.28464@2.1050005	453.2846	2.11	HILIC	NEG	LysoPE 16:0	2	63834	11884	72366	20757	0.28944	0.48	1.10	466155	74250	462278	82088	0.91754	-0.05	0.63
453.2848@9.730997	453.2848	9.73	RP	NEG	LysoPC 14:0	2	49940	6708	56850	12516	0.15382	0.64	0.95	1173980	141443	1166800	141952	0.91615	-0.05	0.41
453.28513@10.174002	453.2851	10.17	RP	NEG	LysoPE 16:0	1	379173	42863	453469	112606	0.07643	0.78	0.96	1632529	130327	1606269	213899	0.75226	-0.14	0.57
453.28513@10.057005	453.2851	10.06	RP	NEG			16101	2790	23983	10937	0.04964	0.86	1.01	216239	18558	217247	26194	0.92508	0.04	0.26
453.28534@1.8419998	453.2853	1.84	HILIC	POS			4571	1426	6167	2639	0.12117	0.69	0.91	30389	2701	30345	3941	0.97787	-0.01	0.37
453.28595@2.1169996	453.2860	2.12	HILIC	POS	1-Palmitoyl-2-hydroxy-sn-glycero-3-phosphoethanolamine	2	33234	5743	36670	9027	0.34024	0.43	1.22	205122	21185	199396	26315	0.61529	-0.24	0.73
453.2863@10.178995	453.2863	10.18	RP	POS	LysoPE 16:0	1	264404	40482	310687	78262	0.12548	0.68	1.03	518966	48506	530423	90516	0.73584	0.15	0.69

453.3222@1.5839998	453.3222	1.58	HILIC	POS										24310	3833	25209	5681	0.69426	0.18	0.57
453.7737@10.475001	453.7737	10.48	RP	POS										74385	8357	72674	12132	0.72797	-0.16	0.75
453.92935@4.4080005	453.9294	4.41	HILIC	NEG			149125	6355	150549	11864	0.74892	0.15	0.61	147349	14396	150918	16284	0.62812	0.23	0.55
455.20834@10.812997	455.2083	10.81	RP	NEG			90198	7152	98619	8522	0.03512	0.94	1.05	80933	6192	78790	7260	0.50799	-0.31	0.82
455.36032@10.321998	455.3603	10.32	RP	POS			478989	33731	478217	27956	0.95919	-0.03	1.02	240415	25758	244639	23280	0.72293	0.17	0.61
456.10822@6.322999	456.1082	6.32	HILIC	NEG			38502	8279	52871	27753	0.14603	0.64	1.00							
457.16748@10.526	457.1675	10.53	RP	NEG			114278	25068	141919	18174	0.02017	1.08	1.22	201084	28845	202753	29203	0.90488	0.06	0.71
457.188@10.5859995	457.1880	10.59	RP	NEG			367659	45910	422020	45052	0.02216	1.03	1.15	158333	33242	135523	25281	0.12994	-0.73	1.38
458.1913@10.526	458.1913	10.53	RP	NEG			32386	7124	40661	4618	0.01378	1.15	1.29							
458.2331@10.804997	458.2331	10.80	RP	NEG										256392	61204	263653	33469	0.76835	0.15	0.92
459.18286@10.648996	459.1829	10.65	RP	NEG			38213	10502	45520	8192	0.12755	0.73	0.93	53647	11346	44008	5883	0.05107	-0.96	1.59
459.2037@10.794004	459.2037	10.79	RP	NEG			431329	51974	467729	40108	0.12421	0.74	0.99	97500	21458	89038	13317	0.34760	-0.47	1.35
461.13913@11.182005	461.1391	11.18	RP	POS			83649	15464	83374	14872	0.97008	-0.02	0.66	77989	31382	94256	46494	0.38912	0.39	0.81
461.21924@11.058005	461.2192	11.06	RP	NEG			146262	15536	166512	21609	0.03310	0.94	1.07	143366	23880	139415	29417	0.75672	-0.15	0.77
461.26004@8.596997	461.2600	8.60	RP	POS	Taurocholic acid	1	104530	113051	210553	183529	0.15083	0.64	0.78	7994	6821	5187	2820	0.30019	-0.54	0.87
462.29883@11.722997	462.2988	11.72	RP	POS										53781	10785	40974	12705	0.03286	-0.95	1.54
462.37125@11.265006	462.3713	11.27	RP	POS			350080	17338	340671	23359	0.34003	-0.44	1.21	260752	85055	287127	79652	0.51060	0.32	0.75
463.30276@10.464997	463.3028	10.46	RP	NEG			95139	18152	103372	22501	0.40154	0.39	1.02	72935	30866	72309	21509	0.96186	-0.02	0.54
463.36703@11.261995	463.3670	11.26	RP	POS			367918	14362	365046	25787	0.76877	-0.13	0.88	230501	74227	245545	72760	0.67186	0.20	0.69
465.16983@10.475004	465.1698	10.48	RP	NEG			40866	8105	42631	5496	0.60707	0.26	0.57	131514	22540	140788	22518	0.39752	0.40	0.95
467.1672@10.476004	467.1672	10.48	RP	NEG			16039	3096	16965	2901	0.52585	0.31	0.56	54300	10909	56387	10370	0.68528	0.20	0.81
467.1728@10.587004	467.1728	10.59	RP	NEG			149708	15531	169961	17287	0.01771	1.05	1.17	129712	16757	114326	12400	0.04766	-0.94	1.65
467.2984@10.010996	467.2984	10.01	RP	NEG			50413	6396	53619	11462	0.46370	0.33	1.24	382865	54964	399537	57677	0.53969	0.29	0.63
467.30167@1.4980004	467.3017	1.50	HILIC	POS	1-Myristoyl-sn-glycero-3-phosphocholine	2	41506	9133	50717	12600	0.08943	0.76	0.95	1682807	168845	1719764	192823	0.67013	0.20	0.55
467.30228@1.3359996	467.3023	1.34	HILIC	POS			7946	1589	8213	1772	0.74031	0.16	0.63	51902	3747	53693	5007	0.39700	0.39	0.81
467.3026@1.4980004	467.3026	1.50	HILIC	POS			33636	7935	40509	9843	0.11831	0.71	0.95	1376980	140804	1405647	158790	0.68999	0.19	0.53
468.06366@6.408998	468.0637	6.41	HILIC	NEG			16772	4622	15417	3641	0.50828	-0.33	0.99							
468.79852@10.802998	468.7985	10.80	RP	POS										63524	14153	62437	17058	0.88417	-0.07	0.56
469.18857@10.793	469.1886	10.79	RP	NEG			244712	23547	267842	25888	0.06334	0.84	1.08	95278	23414	78551	13862	0.09873	-0.81	1.51
471.20425@11.058005	471.2043	11.06	RP	NEG			108300	8978	119308	13648	0.05526	0.85	1.00	120906	10891	114324	12582	0.25009	-0.54	0.96
473.18277@10.791994	473.1828	10.79	RP	NEG			101622	9397	109300	12539	0.15515	0.65	1.09	29651	7622	26741	4328	0.35473	-0.47	1.18
473.1835@10.791994	473.1835	10.79	RP	NEG			115824	12231	127383	14708	0.08545	0.78	1.13	38619	9349	33510	5810	0.20016	-0.64	1.36
474.2892@14.944	474.2892	14.94	RP	NEG										1016895	448174	815956	157309	0.25532	-0.60	1.28
474.40594@12.436998	474.4059	12.44	RP	NEG										53741	7825	51674	15248	0.71502	-0.17	0.87
474.40607@12.3150015	474.4061	12.32	RP	NEG			223243	23623	218087	29335	0.68443	-0.19	1.01	98704	5438	95631	6223	0.27884	-0.51	0.93
474.4074@14.001002	474.4074	14.00	RP	POS			626682	38160	628747	18248	0.89063	0.07	0.97	443282	30344	444591	53245	0.94851	0.03	0.49
474.40765@12.324003	474.4077	12.32	RP	POS			199248	64032	180091	67883	0.54706	-0.29	0.81	9100	1389	11818	6750	0.24035	0.52	0.94
475.19833@10.393003	475.1983	10.39	RP	NEG			7799	1863	9135	3061	0.26969	0.50	0.69	89081	14455	90390	13291	0.84566	0.09	0.26
475.19864@10.475004	475.1986	10.48	RP	NEG			44948	12941	49496	9386	0.41810	0.40	0.65	781198	86486	736888	65884	0.25013	-0.56	1.04
475.26837@2.1159997	475.2684	2.12	HILIC	POS			12684	3018	13401	3379	0.64085	0.22	1.13	56324	9788	54075	10296	0.64176	-0.22	0.71
476.17105@10.526	476.1711	10.53	RP	NEG			38573	5686	43899	2266	0.03161	1.08	1.41	47082	3427	49678	5798	0.25402	0.51	1.28
477.26944@5.745001	477.2694	5.75	HILIC	POS										11247	8885	31735	19910	0.01106	1.08	1.76
477.28433@2.0540006	477.2843	2.05	HILIC	NEG			42500	9878	63561	16648	0.00401	1.20	1.31	341473	56978	334974	75698	0.83777	-0.10	0.41
477.28506@10.016999	477.2851	10.02	RP	NEG	LysoPE 18:2	2	77638	14065	132202	48587	0.00546	1.18	1.29	960318	144094	938023	152301	0.75423	-0.15	0.55
477.28506@9.903005	477.2851	9.90	RP	NEG	LysoPE 18:2	2	242837	54077	295378	37828	0.03555	1.00	1.36	217902	68254	226621	82330	0.80875	0.11	0.36
477.28522@2.0660012	477.2852	2.07	HILIC	POS			18342	4406	29570	8166	0.00186	1.28	1.41	145308	20179	141470	28617	0.74299	-0.15	0.49
477.2859@10.023003	477.2859	10.02	RP	POS	LysoPE 18:2	2	110529	25875	181220	63889	0.00669	1.15	1.23	362531	67804	361160	55705	0.96383	-0.02	0.43
477.28613@9.903999	477.2861	9.90	RP	POS	LysoPE 18:2	2	137182	29519	155239	25280	0.18877	0.63	1.05	103869	21187	100832	18603	0.75395	-0.15	0.38
477.38162@11.252997	477.3816	11.25	RP	POS			491729	15202	497360	26847	0.58280	0.25	1.13	335423	84109	344145	93345	0.83748	0.10	0.55
479.0967@6.616998	479.0967	6.62	HILIC	NEG			22605	12321	35626	19773	0.10566	0.72	0.97							
479.26956@2.187	479.2696	2.19	HILIC	POS			123549	131761	146777	90980	0.67799	0.21	0.29							
479.27072@2.5260005	479.2707	2.53	HILIC	POS			34557	36531	58805	39403	0.19375	0.61	0.74							
479.29932@10.290002	479.2993	10.29	RP	POS	LysoPE 18:1	1	263457	46945	311219	73909	0.11382	0.71	0.97	360649	33580	403680	53844	0.05363	0.85	1.38
479.29996@1.9760002	479.3000	1.98	HILIC	NEG	LysoPE 18:1	2	72829	16281	90358	25023	0.09072	0.75	0.98	403022	104173	460929	102233	0.25363	0.54	0.98
479.3001@10.286003	479.3001	10.29	RP	NEG	LysoPE 18:1	2	363540	53745	423247	95072	0.11223	0.71	0.96	893313	119321	985584	156656	0.17329	0.62	1.01
479.30045@10.173003	479.3005	10.17	RP	NEG			76947	12279	97766	18381	0.01042	1.10	1.18	93231	13152	106254	21701	0.13477	0.67	1.09
479.30072@9.739998	479.3007	9.74	RP	NEG	LysoPC 16:1	2	169872	41689	181135	21479	0.50162	0.34	0.84	630901	118849	643221	169100	0.85830	0.08	0.20
479.30118@2.0040002	479.3012	2.00	HILIC	POS			36353	8121	43953	10016	0.09232	0.76	0.94	125024	22775	144869	24261	0.09168	0.78	1.27
479.3014@9.897002	479.3014	9.90	RP	NEG	LysoPC 16:1	2	164426	17802	188205	39193	0.10829	0.71	0.99	4461386	692477	4069795	361778	0.17418	-0.69	1.16
479.302@10.175997	479.3020	10.18	RP	POS			55207	8594	67042	12039	0.02594	0.97	1.10	42285	4835	47544	5085	0.03828	0.94	1.56
479.3354@1.4960002	479.3354	1.50	HILIC	POS										24328	2703	26631	3937	0.15981	0.64	1.19
479.338@1.1710002	479.3380	1.17	HILIC	POS			26575	7604	30838	10567	0.33352	0.44	1.04	98768	10062	99817	28075	0.91432	0.05	0.37
480.15942@5.399001	480.1594	5.40	HILIC	POS			36336	11922	47936	19305	0.13633	0.67	0.95							

481.18637@10.556	481.1864	10.56	RP	NEG	LysoPE 18:0	1	57086	7065	66472	6272	0.00949	1.16	1.33	57779	8358	52178	6328	0.13803	-0.72	1.26
481.31558@1.9560006	481.3156	1.96	HILIC	NEG			67098	14322	79711	26591	0.21716	0.55	1.07	559543	101079	570209	122177	0.84167	0.09	0.65
481.31567@1.702	481.3157	1.70	HILIC	NEG			7332	1885	10966	3838	0.01895	1.01	1.21	35795	6224	38727	6326	0.33780	0.45	0.93
481.31577@1.3779994	481.3158	1.38	HILIC	NEG			31300	4294	32474	5043	0.60062	0.25	1.16	164769	16445	159896	23045	0.60764	-0.24	0.70
481.31583@9.902006	481.3158	9.90	RP	POS			18976	2559	21391	4613	0.17838	0.60	1.10	217704	23295	225268	27404	0.53493	0.29	0.59
481.31592@10.463997	481.3159	10.46	RP	NEG			18119	4533	26083	11541	0.06614	0.80	0.99	251332	22290	234636	28693	0.18172	-0.61	1.04
481.31604@1.2249997	481.3160	1.22	HILIC	NEG			4887	769	5529	1507	0.25956	0.50	0.93	27879	2160	27755	4223	0.93669	-0.04	0.45
481.3161@10.105001	481.3161	10.11	RP	NEG			550859	70839	686223	219739	0.09077	0.74	0.96	5561350	377161	5506301	490766	0.79096	-0.12	0.56
481.31647@1.4459994	481.3165	1.45	HILIC	POS	1-Pentadecanoyl-sn-glycero-3-phosphocholine	2	51492	11404	65560	20670	0.08551	0.76	0.96	1135115	101917	1191359	114071	0.28469	0.50	0.86
481.3165@10.567995	481.3165	10.57	RP	NEG			632381	105514	755109	209004	0.12612	0.68	1.01	1576201	81869	1502223	176606	0.25818	-0.50	0.85
481.31653@10.221001	481.3165	10.22	RP	NEG	LysoPC 16:0	1	7010148	856977	7525667	1493768	0.37158	0.40	1.03	28293456	1217910	27805178	1410859	0.44131	-0.36	0.71
481.31686@1.278	481.3169	1.28	HILIC	POS			7941	1154	9058	2397	0.21404	0.55	0.91	65328	6375	70949	5713	0.06920	0.85	1.48
481.31696@1.9749992	481.3170	1.97	HILIC	POS	1-Octadecanoyl-sn-glycero-3-phosphoethanolamine	2	29893	6597	35139	11102	0.23042	0.54	1.03	142649	14315	143476	22651	0.92596	0.04	0.49
481.3178@10.0079975	481.3178	10.01	RP	POS	LysoPC 15:0	2	70432	10285	82657	24344	0.17287	0.60	1.04	979656	105698	1005457	80633	0.57712	0.28	0.47
481.3179@10.575004	481.3179	10.58	RP	POS	LysoPE 18:0	1	465475	100593	588663	192624	0.10026	0.73	1.05	751303	69242	753704	108110	0.95513	0.03	0.32
481.35068@1.4589998	481.3507	1.46	HILIC	POS			56071	10258	75088	24827	0.04527	0.87	0.97	759788	35028	780087	141072	0.66917	0.19	0.49
481.35117@10.721002	481.3512	10.72	RP	POS			130751	20629	163747	50656	0.08211	0.76	1.02	925913	89033	936246	148330	0.85705	0.08	0.36
481.9775@8.897002	481.9775	8.90	RP	NEG			182241	3099	182718	3897	0.77550	0.13	1.36	177163	6825	178359	8114	0.73813	0.16	0.54
483.2709@6.919999	483.2709	6.92	HILIC	POS			17589	2776	19508	9535	0.55633	0.26	1.13							
484.3523@1.7400005	484.3523	1.74	HILIC	POS			33868	27747	35640	29920	0.89803	0.06	1.31	100401	34182	108136	39451	0.66142	0.21	0.51
484.3525@1.59	484.3525	1.59	HILIC	POS			29647	14050	32783	14679	0.70080	0.22	1.09	109862	38463	120724	44937	0.58757	0.26	0.48
484.41324@11.854002	484.4132	11.85	RP	POS			93562	41304	63628	18467	0.08463	-0.87	1.26							
489.16876@6.302001	489.1688	6.30	HILIC	NEG			289197	78151	438788	291956	0.14710	0.64	0.85							
491.18143@10.557005	491.1814	10.56	RP	NEG			34109	5602	38683	4787	0.08548	0.81	1.04	74179	9295	68806	13914	0.34127	-0.44	0.86
491.18622@10.487	491.1862	10.49	RP	NEG			43912	12350	49655	17592	0.42752	0.37	0.64	15608	4353	11535	4390	0.06577	-0.84	1.47
491.23416@6.648001	491.2342	6.65	HILIC	POS			16006	4327	14608	2293	0.42571	-0.41	1.12							
491.30136@1.4580001	491.3014	1.46	HILIC	POS			1897	516	2670	599	0.00878	1.13	1.21	67184	5165	66244	6677	0.74039	-0.15	0.36
491.43448@12.325005	491.4345	12.33	RP	POS			450428	147633	402986	157511	0.51935	-0.31	0.78	14654	2388	20713	12988	0.17671	0.59	1.03
492.42@11.959	492.4200	11.96	RP	POS			113404	40994	85658	18793	0.10503	-0.82	1.12							
493.20062@10.821998	493.2006	10.82	RP	NEG			8650	2558	6639	2717	0.12469	-0.71	1.23	85003	16933	83749	15999	0.87480	-0.08	0.70
493.3157@10.14	493.3157	10.14	RP	NEG										293264	42262	283948	34547	0.62191	-0.24	0.51
493.31726@1.264	493.3173	1.26	HILIC	POS			76398	19342	77759	11983	0.86455	0.09	0.98	333235	37480	326858	26944	0.69174	-0.20	0.44
493.3174@9.915001	493.3174	9.92	RP	POS	LysoPC 16:1	1	125075	24716	159840	45432	0.05524	0.84	1.03	6182905	976498	5770563	593877	0.31388	-0.51	0.87
493.31754@9.893001	493.3175	9.89	RP	POS	LysoPC 16:1	1								517971	120092	468890	56524	0.30990	-0.52	0.94
493.31757@1.4259995	493.3176	1.43	HILIC	POS			151990	34011	186725	45023	0.07879	0.79	0.98	3564796	460579	3433154	358064	0.51705	-0.32	0.56
495.33203@10.497003	495.3320	10.50	RP	NEG	LysoPC 17:0	2	225518	38448	254086	58794	0.23135	0.54	0.93	2287865	130912	2237413	286512	0.62732	-0.22	0.82
495.33286@10.239997	495.3329	10.24	RP	POS	LysoPC 16:0	1	9257084	1639707	11072841	3993450	0.21253	0.55	1.06	77848796	2454467	76430957	4879105	0.43535	-0.35	0.60
495.3332@1.3909997	495.3332	1.39	HILIC	POS	LysoPC 16:0	2	6853522	1351591	7834627	2477361	0.30082	0.47	1.06	59964847	1491143	59303147	3872685	0.62769	-0.22	0.39
495.3332@10.109001	495.3332	10.11	RP	POS			552305	107061	817577	403906	0.07028	0.79	0.98	8442075	616679	8638593	1110128	0.64075	0.21	0.53
495.3335@1.2350004	495.3335	1.24	HILIC	POS	LysoPC 16:0, isomer	2	627679	112093	820702	294361	0.07806	0.77	0.99	10196540	569990	10164586	853060	0.92548	-0.04	0.36
495.9767@8.421	495.9767	8.42	RP	NEG			386051	21879	382513	24727	0.75143	-0.15	1.04	249768	5261	250925	7326	0.70166	0.18	0.87
496.3297@12.437995	496.3297	12.44	RP	NEG			5758	1476	5547	1439	0.76457	-0.14	1.07	407981	86517	390758	88645	0.68318	-0.20	0.59
497.28098@2.187	497.2810	2.19	HILIC	POS			65321	69560	80093	50113	0.62144	0.25	0.32							
497.28156@7.3389997	497.2816	7.34	RP	POS			113511	124926	185264	137570	0.26248	0.52	0.59	6241	6517	5796	2493	0.85889	-0.09	0.38
497.30823@9.850999	497.3082	9.85	RP	NEG										186657	26820	195013	38815	0.59679	0.24	0.56
497.8946@4.4250016	497.8946	4.43	HILIC	POS			39258	2799	43048	5490	0.07663	0.78	0.93	70278	2461	72129	3958	0.24118	0.53	1.03
499.26862@2.0749996	499.2686	2.07	HILIC	POS			11476	3712	16011	4055	0.02386	1.01	1.13	44108	8002	43307	10894	0.85939	-0.08	0.54
499.26904@1.9760002	499.2690	1.98	HILIC	NEG										36008	9662	34097	7866	0.65745	-0.22	0.69
499.2692@9.759999	499.2692	9.76	RP	NEG										76808	16299	70805	14903	0.43157	-0.38	0.95
499.29514@1.6819994	499.2951	1.68	HILIC	NEG			27953	26358	225963	239033	0.02591	0.97	1.16	12500	6636	36524	30549	0.03391	0.92	1.89
499.29535@9.203002	499.2954	9.20	RP	NEG			417040	475759	924180	715915	0.08949	0.76	0.95	53743	45065	29029	13932	0.16830	-0.72	1.19
499.2957@9.299997	499.2957	9.30	RP	NEG	Tauroursodeoxycholic acid	2	39560	39233	201873	207391	0.03413	0.92	1.09	32093	54597	24263	9970	0.69887	-0.21	0.37
499.2958@7.9190035	499.2958	7.92	RP	NEG			381364	448127	724868	501855	0.14317	0.68	0.78	56107	22369	41342	14266	0.12895	-0.75	1.29
499.2958@1.4780004	499.2958	1.48	HILIC	NEG	Tauroursodeoxycholic acid	2	236032	266063	822836	702070	0.02966	0.94	1.11	57125	36820	59207	31595	0.90061	0.06	0.95
499.29617@9.412996	499.2962	9.41	RP	NEG	Tauroursodeoxycholic acid	2	223799	206936	1863681	1765077	0.01498	1.06	1.25	62737	56207	87554	52665	0.35221	0.45	1.19
499.3869@10.330996	499.3869	10.33	RP	POS			114893	17084	110964	16243	0.62663	-0.23	0.99	61272	5949	61952	4903	0.79816	0.13	0.66
500.05826@5.334	500.0583	5.33	HILIC	NEG			124183	5410	127490	8393	0.32605	0.45	0.92	7134	985	8149	3534	0.40299	0.37	0.78
501.2128@10.578002	501.2128	10.58	RP	NEG			19128	6418	23629	6487	0.15925	0.66	0.78	73562	16372	57363	15501	0.04781	-0.91	1.52
501.21332@10.485994	501.2133	10.49	RP	NEG			59928	18983	71148	30551	0.35355	0.42	0.68	71578	21414	44602	26704	0.02867	-0.97	1.65
501.27792@10.006998	501.2779	10.01	RP	NEG	LysoPE 20:4	2	64527	14745	111583	53969	0.02193	0.99	1.06	2526635	309335	2059817	270242	0.00396	-1.26	2.04
501.28464@9.903005	501.2846	9.90	RP	NEG	LysoPE 20:4	2	347196	56573	377589	51472	0.25542	0.54	0.91	736872	166026	650793	213974	0.34932	-0.43	0.78
501.28473@1.925	501.2847	1.93	HILIC	NEG			57077	13532	76693	20251	0.02488	0.98	1.05	1578230	245790	1318128	241821	0.03819	-0.94	1.56
501.28494@1.6679995	501.2849	1.67	HILIC	NEG			29036	5614	34655	16237	0.32627	0.43	0.82	126509	21054	102535	18393	0.02195	-1.04	1.76

501.2852@1.944	501.2852	1.94	HILIC	POS			36770	7891	50563	15330	0.02569	0.96	1.05	508396	72809	427787	68534	0.02864	-0.99	1.63
501.28586@9.903999	501.2859	9.90	RP	POS	LysoPE 20:4	2	204223	35987	201931	33665	0.89179	-0.07	1.08	290560	38365	231049	31860	0.00295	-1.30	2.13
501.28632@1.6800001	501.2863	1.68	HILIC	POS			25098	3584	23732	5030	0.51030	-0.30	1.05	53512	7708	44102	7606	0.01909	-1.05	1.73
501.28653@10.0079975	501.2865	10.01	RP	POS	LysoPE 20:4	1	102732	22189	166321	61544	0.00960	1.11	1.18	1064574	121639	896317	128114	0.01090	-1.12	1.81
501.9833@8.897002	501.9833	8.90	RP	NEG			46829	1037	46527	1739	0.65402	-0.20	0.78	49400	1996	48693	2507	0.51376	-0.31	0.58
503.22986@10.802001	503.2299	10.80	RP	NEG			27335	8577	30551	12320	0.52319	0.29	0.85	812088	87304	749827	82697	0.14252	-0.69	1.21
503.22995@10.730996	503.2300	10.73	RP	NEG			4323	1188	4766	1456	0.48694	0.33	0.91	330184	51611	307673	49148	0.36100	-0.44	0.80
503.2809@12.438998	503.2809	12.44	RP	NEG										144730	15764	134686	25954	0.32562	-0.45	0.96
503.2985@1.9739993	503.2985	1.97	HILIC	POS			16772	3270	19739	5786	0.18898	0.59	1.01	93623	14119	93889	19878	0.97391	0.02	0.37
503.29987@10.183004	503.2999	10.18	RP	NEG			7565	1672	9576	2288	0.04530	0.89	1.25	84798	17273	92690	25553	0.44630	0.35	1.08
503.2999@9.833002	503.2999	9.83	RP	NEG	LysoPC 18:3	2								753313	165633	584301	115045	0.02826	-1.03	1.69
505.18805@10.526	505.1881	10.53	RP	NEG			46385	8059	55680	4919	0.01369	1.16	1.49	56953	6605	56002	7730	0.78168	-0.13	0.72
505.31622@10.048003	505.3162	10.05	RP	NEG	LysoPC 18:2	2	848205	96355	1107750	249526	0.00951	1.11	1.18	15594925	934615	15926465	1087062	0.49578	0.32	0.75
505.31644@9.9349985	505.3164	9.93	RP	NEG	LysoPC 18:2	1	2376865	492811	2664423	250429	0.16048	0.71	0.99	5272722	1015182	5937699	1702891	0.31879	0.45	0.78
507.33044@1.3129998	507.3304	1.31	HILIC	NEG			7881	1175	8990	1888	0.14583	0.65	0.85	62164	9530	60508	7371	0.69185	-0.20	0.61
507.33124@10.230997	507.3312	10.23	RP	POS			63187	15765	93721	50287	0.09431	0.73	0.98	3740902	255012	3782878	258796	0.73446	0.16	0.47
507.3314@10.1900015	507.3314	10.19	RP	NEG	LysoPC 18:1	1	943652	158963	1024428	121507	0.25431	0.56	0.96	871306	216795	1035214	313258	0.20745	0.57	0.95
507.3321@10.386995	507.3321	10.39	RP	NEG	LysoPC 18:1	2	2394257	248416	2686049	527784	0.14283	0.65	1.02	31343927	2292350	30165301	2874280	0.34595	-0.44	0.83
507.33243@10.005003	507.3324	10.01	RP	POS			18617	4421	21371	2498	0.14189	0.73	0.87	452104	77393	422662	77467	0.43372	-0.37	0.66
507.33328@1.2140005	507.3333	1.21	HILIC	POS			6875	1789	7792	1356	0.24966	0.56	0.95	55496	7698	57033	7303	0.67239	0.20	0.50
507.36536@1.3880004	507.3654	1.39	HILIC	POS			22169	3612	26873	7549	0.10280	0.72	1.00	135309	20549	141874	32668	0.60988	0.23	0.59
508.27872@1.4990002	508.2787	1.50	HILIC	NEG										25212	4574	25716	8349	0.87271	0.07	0.78
508.33664@10.078001	508.3366	10.08	RP	POS										349815	29262	354305	39488	0.78505	0.13	0.37
509.34244@10.239997	509.3424	10.24	RP	POS			23970	4034	35646	19458	0.09139	0.74	0.95	1921094	303112	2089279	318053	0.26792	0.52	1.02
509.3466@1.2900007	509.3466	1.29	HILIC	NEG			23000	2512	23657	3513	0.64980	0.21	0.95	67483	8322	63468	11083	0.39185	-0.40	0.96
509.34717@10.679995	509.3472	10.68	RP	NEG	LysoPC 18:0	1	6773527	1095697	7364059	1885761	0.41812	0.37	1.00	38640244	2827316	36148281	3661441	0.12069	-0.70	1.18
509.34872@1.3460004	509.3487	1.35	HILIC	POS			187467	31752	210406	49320	0.24875	0.52	0.98	1062511	64815	1063796	106335	0.97518	0.01	0.52
509.34894@10.578002	509.3489	10.58	RP	POS	LysoPC 17:0	2	437251	70854	532249	159120	0.11245	0.70	1.01	4905597	257594	4850529	432322	0.74162	-0.15	0.60
509.349@1.1959994	509.3490	1.20	HILIC	POS	1-Heptadecanoyl-sn-glycero-3-phosphocholine	2	24101	4929	31669	10186	0.05637	0.83	1.00	331188	22339	335551	50493	0.80995	0.11	0.73
510.33923@10.425001	510.3392	10.43	RP	POS			91221	2011	95788	3846	0.00521	1.17	1.44							
510.42865@11.959	510.4287	11.96	RP	POS			149488	68587	98321	35422	0.08092	-0.87	1.09							
511.3277@9.859995	511.3277	9.86	RP	POS			13414	2680	16995	5007	0.07018	0.80	0.91	232075	25829	252650	42171	0.22024	0.55	0.92
511.32803@1.983001	511.3280	1.98	HILIC	POS			7483	1237	8705	2619	0.21162	0.56	1.06	259136	34853	273836	48576	0.46514	0.34	0.59
511.32892@1.7110002	511.3289	1.71	HILIC	POS										23763	3218	25420	4783	0.39310	0.39	0.67
513.28735@12.438002	513.2874	12.44	RP	NEG										47267	4751	44398	9651	0.42295	-0.36	0.88
513.3054@1.4889995	513.3054	1.49	HILIC	NEG			3938	1611	2689	1533	0.17855	-0.74	1.18	95263	16041	95396	13511	0.98530	0.01	0.59
515.28204@12.438997	515.2820	12.44	RP	NEG			8332	1616	7763	1731	0.48198	-0.33	0.65	957770	99656	893693	204916	0.39856	-0.38	0.89
515.2902@2.194	515.2902	2.19	HILIC	NEG	Taurocholic acid	2	1965008	2144487	2551685	1611126	0.53074	0.31	0.43	87713	105434	37382	26559	0.22099	-0.65	1.05
515.2906@8.510998	515.2906	8.51	RP	NEG	Taurocholic acid	2	3670878	4059588	8121758	7002016	0.11044	0.71	0.89	247836	267001	149978	119308	0.35730	-0.48	0.78
515.2907@7.281003	515.2907	7.28	RP	NEG	Taurocholic acid	2	4572815	5130362	9336595	7003246	0.11353	0.71	0.87	241420	246225	269338	119398	0.77408	0.15	0.63
515.2907@3.492	515.2907	3.49	HILIC	NEG										90597	92396	101423	90036	0.80571	0.12	0.44
515.291@3.2219996	515.2910	3.22	HILIC	NEG			453705	482446	1469851	1177471	0.02655	0.96	1.20	21777	15066	36187	17774	0.07923	0.80	1.63
515.29156@8.6050005	515.2916	8.61	RP	POS			114043	139675	278439	353427	0.20043	0.57	0.67	6643	5602	4563	2825	0.35870	-0.47	0.77
517.21094@6.302001	517.2109	6.30	HILIC	NEG			40105	6814	62191	39288	0.10944	0.70	0.87							
517.31506@1.2249997	517.3151	1.22	HILIC	POS			29156	5607	32726	7488	0.26290	0.51	1.12	124598	19197	108156	14218	0.06250	-0.89	1.46
517.3161@10.579003	517.3161	10.58	RP	POS			223655	40884	275085	89934	0.12832	0.67	1.06	321605	72923	285668	79978	0.33313	-0.46	0.94
517.31665@10.446005	517.3167	10.45	RP	POS			97801	23636	128422	51091	0.11333	0.70	1.00	102930	19104	92804	16646	0.25436	-0.55	0.97
517.3177@9.839998	517.3177	9.84	RP	POS										1316566	289947	1047022	213432	0.04506	-0.95	1.54
517.4864@11.876005	517.4864	11.88	RP	POS			154130	29952	136974	16776	0.17328	-0.68	1.37	3684	771	2685	563	0.00828	-1.20	1.95
519.07605@6.544999	519.0761	6.54	HILIC	NEG			74216	30460	145149	113060	0.08224	0.76	1.00							
519.333@10.089997	519.3330	10.09	RP	POS	LysoPC 18:2	1	1052581	196287	1668340	678925	0.01826	1.02	1.11	35946777	2243861	36855872	2868942	0.46041	0.34	0.62
519.33356@1.3559992	519.3336	1.36	HILIC	POS			1178036	243192	1684621	487404	0.01147	1.08	1.16	22503064	1089505	23300677	2244025	0.33878	0.43	0.81
519.33356@9.936003	519.3336	9.94	RP	POS	LysoPC 18:2	1	1782294	434994	2008772	370345	0.25880	0.54	0.93	7256227	996531	7650307	1331318	0.48211	0.33	0.56
519.33374@1.2060002	519.3337	1.21	HILIC	POS			1092451	224589	1113079	233170	0.85122	0.09	0.95	5412705	446087	5585467	648902	0.51308	0.30	0.55
519.5015@12.287002	519.5015	12.29	RP	POS			80057	24320	63127	26484	0.17577	-0.63	0.85							
520.099@5.377999	520.0990	5.38	HILIC	POS			20486	3702	25520	5717	0.03734	0.91	1.14							
521.3466@10.597997	521.3466	10.60	RP	NEG										197318	23849	205816	34105	0.54269	0.28	0.82
521.3485@1.3240004	521.3485	1.32	HILIC	POS	1-(9Z-Octadecenoyl)-sn-glycero-3-phosphocholine	2	1372070	228865	1615528	387277	0.11589	0.70	1.00	20101040	1511900	20037904	1416144	0.92894	-0.04	0.36
521.34863@10.4349985	521.3486	10.43	RP	POS	LysoPC 18:1	1	1115443	273951	1633688	828390	0.08700	0.75	1.01	39892352	3275143	38841277	3101772	0.49806	-0.33	0.61
521.34863@1.1739999	521.3486	1.17	HILIC	POS	1-(9Z-Octadecenoyl)-sn-glycero-3-phosphocholine	2	573651	85194	611694	112767	0.42575	0.37	1.07	5095126	622553	4987502	563990	0.70892	-0.18	0.47
521.34894@10.724	521.3489	10.72	RP	POS			11919442	2846848	15178636	6480480	0.17482	0.60	1.03	68080016	3860291	63321168	6243241	0.06380	-0.82	1.36
521.34906@10.433997	521.3491	10.43	RP	POS	LysoPC 18:1	1	2251447	408210	3074790	1250392	0.07307	0.78	1.02	61966473	5112969	59762697	5213420	0.38010	-0.42	0.73
521.3495@10.22	521.3495	10.22	RP	POS	LysoPC 18:1	1	1029731	176570	1208616	268787	0.10768	0.72	1.06	3476966	341354	3558129	646480	0.73695	0.15	0.39

521.45123@1.3260001	521.4512	1.33	HILIC	POS										1082401	156034	1046646	136415	0.61663	-0.24	0.59
521.58997@10.619997	521.5900	10.62	HILIC	POS			39240	1183	38209	2019	0.19496	-0.58	1.08	38400	2591	38408	2393	0.99482	0.00	0.60
521.9169@4.3769994	521.9169	4.38	HILIC	NEG			92434	3328	94707	5171	0.27478	0.50	1.25	129683	10794	133054	13963	0.57074	0.26	0.58
522.09717@5.377999	522.0972	5.38	HILIC	POS			14479	2494	18203	4096	0.02959	0.95	1.07							
522.32886@12.4400015	522.3289	12.44	RP	NEG			10129	1297	9928	2209	0.81260	-0.11	0.90	308104	21747	298225	29852	0.42779	-0.37	0.79
522.3512@10.433004	522.3512	10.43	RP	POS			332538	87403	492717	239695	0.07194	0.79	1.01	11759366	882938	11426191	858616	0.43181	-0.38	0.69
523.2677@1.9520004	523.2677	1.95	HILIC	POS			11549	2617	15031	4432	0.05396	0.85	0.97	127244	21715	107034	26709	0.09356	-0.76	1.27
523.3624@10.962004	523.3624	10.96	RP	NEG	LysoPC 19:0	2	162210	37298	162006	38255	0.99103	-0.01	1.11	1420044	230437	1257767	280903	0.19492	-0.60	1.04
523.36395@1.1549999	523.3640	1.15	HILIC	POS			324788	80903	430267	166536	0.09860	0.73	0.94	4193975	310879	3984518	674904	0.39695	-0.38	0.80
523.3647@1.2970006	523.3647	1.30	HILIC	POS	1-Stearoyl-2-hydroxy-sn-glycero-3-phosphocholine	2	3900507	729763	4246909	1166083	0.45200	0.34	1.10	23387688	1074361	22078688	2196488	0.11876	-0.69	1.17
523.36487@10.724	523.3649	10.72	RP	POS	LysoPC 18:0	1	3350779	1086101	4531513	2431834	0.19044	0.58	1.01	47113632	2997020	43471155	5388422	0.08804	-0.75	1.30
523.3649@10.812003	523.3649	10.81	RP	POS			5018519	974914	6256493	2283519	0.14388	0.65	1.05	10115255	1380427	10043466	1530623	0.91799	-0.05	0.39
523.8791@10.475999	523.8791	10.48	RP	POS										128707	15159	125023	21993	0.67994	-0.19	0.80
525.2844@1.8840001	525.2844	1.88	HILIC	NEG			15711	3691	22914	6027	0.00628	1.15	1.24	1036973	184378	847330	175578	0.04093	-0.93	1.55
525.28467@1.6399996	525.2847	1.64	HILIC	NEG			6655	1848	7592	1747	0.28875	0.51	0.88	132111	23660	104120	21996	0.02004	-1.05	1.74
525.2847@9.903005	525.2847	9.90	RP	NEG			71546	10986	86726	11774	0.01150	1.11	1.37	908690	209927	763469	219074	0.17030	-0.64	1.09
525.2853@1.9030001	525.2853	1.90	HILIC	POS			12296	3037	17115	4744	0.01809	1.02	1.09	425824	61784	343659	59146	0.01100	-1.13	1.83
525.2857@9.997002	525.2857	10.00	RP	NEG	LysoPE 22:6	2	17095	2665	30286	13565	0.01205	1.09	1.16	1850361	275397	1480575	234196	0.00821	-1.18	1.93
525.2859@1.6520002	525.2859	1.65	HILIC	POS			5034	861	5233	812	0.62337	0.24	0.57	54763	8648	44088	7986	0.01572	-1.08	1.77
525.2873@10.002998	525.2873	10.00	RP	POS	LysoPE 22:6	1	26978	5857	47305	19593	0.00886	1.12	1.20	1341807	191144	1119247	150805	0.01678	-1.10	1.78
525.2875@9.903005	525.2875	9.90	RP	POS			44376	7405	47659	6426	0.33673	0.46	0.88	322291	48708	254520	34260	0.00501	-1.27	2.06
525.43994@11.630001	525.4399	11.63	RP	POS			103619	45588	75810	21596	0.14083	-0.75	1.11							
527.3001@1.8840001	527.3001	1.88	HILIC	NEG			2980	1074	4590	1153	0.00692	1.17	1.29	91567	16473	72065	16029	0.02181	-1.03	1.68
527.30023@9.675997	527.3002	9.68	RP	NEG			9951	2205	10718	1020	0.38302	0.45	0.89	99088	15209	135236	61024	0.09733	0.73	1.19
527.30225@10.218997	527.3023	10.22	RP	NEG			4822	1520	9343	3072	0.02157	1.35	1.44	65882	17371	53795	16496	0.15224	-0.67	1.13
527.31067@10.128999	527.3107	10.13	RP	NEG										80501	16070	60864	7905	0.00896	-1.25	2.02
527.3219@1.4290006	527.3219	1.43	HILIC	NEG			6530	937	7186	1781	0.33054	0.44	0.99	75897	13490	74883	11966	0.86979	-0.08	0.57
527.35706@10.604003	527.3571	10.60	RP	NEG			59436	7998	68153	16283	0.15828	0.63	1.01	339949	13868	348888	32265	0.44271	0.34	0.65
527.4553@11.960003	527.4553	11.96	RP	POS	DG 10:0_18:1	2	170294	79231	108696	37537	0.06822	-0.91	1.20							
528.34406@10.425997	528.3441	10.43	RP	NEG			156404	12235	170288	15515	0.04791	0.88	1.05	249199	15979	239971	20432	0.29650	-0.48	0.96
529.3153@1.2660002	529.3153	1.27	HILIC	NEG										34353	5292	33542	3085	0.70750	-0.19	0.65
529.31573@9.933004	529.3157	9.93	RP	NEG	LysoPC 20:4	1	604819	88272	642762	87434	0.37548	0.42	0.67	2515431	463748	2688224	835860	0.58614	0.25	0.50
529.31586@10.045004	529.3159	10.05	RP	NEG	LysoPC 20:4	2	235442	23780	301267	84335	0.03654	0.91	1.00	8036056	556504	7664849	449701	0.14676	-0.70	1.14
530.4011@11.752007	530.4011	11.75	RP	POS			54675	5481	53255	3486	0.53514	-0.31	0.88	70550	13929	72168	20846	0.84644	0.09	0.60
530.4097@11.630998	530.4097	11.63	RP	POS			86497	32149	65420	18203	0.12398	-0.77	1.19							
530.47015@14.001002	530.4702	14.00	RP	POS			682567	42281	684639	22986	0.90292	0.06	0.77	340996	36608	352303	79855	0.69603	0.18	0.60
531.26117@11.11	531.2612	11.11	RP	NEG										57297	13907	51306	15547	0.40042	-0.40	1.02
531.26764@9.891002	531.2676	9.89	RP	POS										194666	33482	174485	16405	0.14661	-0.74	1.30
531.3078@10.2579975	531.3078	10.26	RP	NEG	LysoPC 20:3	2	225174	18134	244042	36876	0.17636	0.60	0.93	394843	37107	387199	27288	0.63378	-0.24	0.79
531.3078@1.5740006	531.3078	1.57	HILIC	NEG			14442	2221	14320	1833	0.90227	-0.06	1.06	182309	24192	177805	27400	0.71577	-0.17	0.49
531.30786@1.4059993	531.3079	1.41	HILIC	NEG			163782	13028	156614	10501	0.22584	-0.59	1.60	58444	6972	56160	8268	0.53303	-0.29	0.78
531.30804@1.2249997	531.3080	1.22	HILIC	NEG			28488	4556	31900	5720	0.17605	0.62	0.85	44725	5055	45120	8081	0.90080	0.06	0.44
531.9741@9.325003	531.9741	9.33	RP	NEG			52910	1719	52582	2199	0.72618	-0.16	0.96	50034	1902	52063	3374	0.12680	0.68	1.15
533.12915@6.050002	533.1292	6.05	HILIC	NEG			27281	4437	30535	6534	0.22627	0.55	0.99	16979	2393	19222	4105	0.16636	0.62	1.04
533.28143@10.259002	533.2814	10.26	RP	POS			240096	38123	259624	62782	0.42711	0.36	1.09	476982	64497	459978	33490	0.51232	-0.34	0.72
533.282@10.252997	533.2820	10.25	RP	POS			239951	37125	259403	64009	0.43150	0.36	1.10	471780	61918	460312	31778	0.64305	-0.24	0.62
533.28326@10.108001	533.2833	10.11	RP	POS			26052	4538	31409	9254	0.12903	0.67	0.91	170223	23321	160289	11128	0.29170	-0.54	1.01
533.3057@1.4070004	533.3057	1.41	HILIC	NEG			72292	5739	68525	4719	0.15492	-0.68	1.79	25970	2948	25866	3541	0.94625	-0.03	0.48
535.1581@11.548003	535.1581	11.55	RP	POS			270430	27230	273670	30733	0.81551	0.11	0.64	354858	51885	444155	177868	0.15693	0.62	1.09
535.3646@1.2770003	535.3646	1.28	HILIC	POS			9059	1532	10290	2599	0.22806	0.54	1.12	106003	9614	114115	12515	0.13736	0.68	1.35
535.49524@11.865998	535.4952	11.87	RP	NEG			128285	34695	110768	15917	0.21407	-0.64	1.38							
535.4971@11.877	535.4971	11.88	RP	POS			164984	32393	146569	17524	0.17410	-0.68	1.24							
536.1879@6.150001	536.1879	6.15	HILIC	POS			472425	101386	420500	83803	0.26177	-0.54	1.55							
537.1022@6.6260004	537.1022	6.63	HILIC	NEG			72276	24921	101806	67264	0.22163	0.54	0.96							
537.1571@11.548003	537.1571	11.55	RP	POS			86669	8439	87242	9224	0.89228	0.06	0.75	111072	17582	138199	54908	0.16610	0.61	1.07
537.27374@2.1959996	537.2737	2.20	HILIC	POS			56185	49745	68408	25480	0.54006	0.31	0.49							
537.3797@1.2669995	537.3797	1.27	HILIC	POS			119213	32462	124126	30185	0.74621	0.16	0.97	821267	136357	767898	146605	0.43555	-0.37	0.85
537.3799@1.134	537.3799	1.13	HILIC	POS			15569	4467	18991	6579	0.20709	0.57	0.83	165316	32617	157432	37541	0.63960	-0.22	0.80
537.3801@11.0599985	537.3801	11.06	RP	POS			308840	78793	353185	91402	0.28393	0.50	0.91	2919375	570606	2595520	627947	0.26782	-0.52	0.94
537.51086@12.273994	537.5109	12.27	RP	NEG			170610	40271	145689	42197	0.21828	-0.58	0.81	26641	4936	24704	7625	0.52374	-0.29	0.88
539.3218@9.898003	539.3218	9.90	RP	NEG			112299	11695	124471	24150	0.18125	0.59	1.04	3300580	519358	3001872	271240	0.16750	-0.70	1.17
539.3389@10.342003	539.3389	10.34	RP	NEG			606416	159145	741014	142889	0.08033	0.82	1.01	92539	27065	79013	17511	0.24272	-0.58	1.20
541.0615@7.7020006	541.0615	7.70	HILIC	POS			3744	9373	7029	9059	0.46373	0.35	0.93							

541.3144@10.071999	541.3144	10.07	RP	POS			317823	61877	516305	239809	0.02771	0.96	1.05	11550212	777849	11066396	522530	0.15473	-0.70	1.21
541.3149@9.932005	541.3149	9.93	RP	POS			184622	53769	220590	62896	0.20764	0.58	0.85	1123661	196584	1240854	186903	0.21645	0.59	1.08
541.3151@1.3310003	541.3151	1.33	HILIC	POS			28359	5204	39621	10860	0.01120	1.08	1.30	209259	32664	251068	31734	0.01421	1.09	1.87
541.3152@1.1949998	541.3152	1.19	HILIC	POS			43655	6733	46723	9349	0.42948	0.36	0.82	90698	11214	99004	11124	0.13544	0.70	1.23
541.31714@9.813002	541.3171	9.81	RP	POS										623062	170260	698537	155252	0.34536	0.45	0.88
541.33673@1.2260005	541.3367	1.23	HILIC	NEG			109022	20834	129407	38132	0.16835	0.62	1.00	1045624	132305	1037263	169246	0.90767	-0.05	0.60
541.33685@1.3769997	541.3369	1.38	HILIC	NEG			641064	93785	689836	153397	0.41807	0.37	1.10	5544010	767640	5340650	946679	0.62088	-0.23	0.75
541.3372@10.105001	541.3372	10.11	RP	NEG			564819	72918	699685	224570	0.09844	0.72	0.96	5482906	350114	5464040	527550	0.92867	-0.04	0.34
542.371@10.740998	542.3710	10.74	RP	NEG			37792	7601	45017	19881	0.30930	0.45	1.11							
543.33093@10.589997	543.3309	10.59	RP	POS			204649	67514	326568	203491	0.09973	0.72	0.97	1541727	344202	1248554	285444	0.07149	-0.85	1.46
543.33105@10.391999	543.3311	10.39	RP	POS			100245	32218	185584	131964	0.07311	0.78	1.00	758807	61871	754086	63933	0.87589	-0.07	0.55
543.33167@10.579003	543.3317	10.58	RP	POS			193546	31991	244031	67619	0.05446	0.84	1.05	523485	134491	458642	144719	0.33914	-0.45	0.93
543.3328@1.2760007	543.3328	1.28	HILIC	POS			284697	52275	386611	107560	0.01873	1.01	1.09	11802406	858618	11480141	969815	0.46492	-0.34	0.59
543.3331@1.1409999	543.3331	1.14	HILIC	POS			307200	43115	305812	60499	0.95540	-0.03	0.85	2527506	337071	2384720	266605	0.34352	-0.46	0.80
543.3333@9.933998	543.3333	9.93	RP	POS	LysoPC 20:4	1	592165	117735	625057	108476	0.55032	0.29	0.85	3634046	461653	3689070	700225	0.84378	0.09	0.35
543.3334@10.066996	543.3334	10.07	RP	POS	LysoPC 20:4	1	329034	49684	530385	230837	0.02112	1.00	1.10	18093883	1529252	17229571	1170971	0.20740	-0.61	1.06
544.3319@10.729003	544.3319	10.73	RP	POS			2383596	603409	3003330	1262959	0.19125	0.58	1.03	3680910	819407	3281605	726627	0.29608	-0.50	0.99
545.3463@11.061003	545.3463	11.06	RP	POS			132745	30259	189774	91064	0.08683	0.75	1.01	323167	98977	320066	103786	0.94920	-0.03	0.65
545.3464@1.2859995	545.3464	1.29	HILIC	POS			248284	35969	295939	66016	0.06894	0.80	0.94	3471335	557547	3930598	634712	0.12009	0.71	1.28
545.3466@1.1450003	545.3466	1.15	HILIC	POS			115760	19195	126122	24202	0.32447	0.46	0.82	869106	178653	953232	190208	0.34789	0.44	0.82
545.34686@10.730002	545.3469	10.73	RP	POS			1562165	349239	1815274	584198	0.27070	0.50	1.10	2326036	586195	2039447	478493	0.28128	-0.52	1.08
545.34735@10.802001	545.3474	10.80	RP	POS			1437316	311550	1839061	737168	0.14137	0.65	1.04	1673209	399988	1461286	325138	0.24390	-0.56	1.03
545.3484@10.240999	545.3484	10.24	RP	POS										2646513	544239	2968236	624508	0.25862	0.53	1.00
545.9739@8.897002	545.9739	8.90	RP	NEG			541286	10683	543586	11629	0.66793	0.20	1.02	574081	28058	577105	29158	0.82592	0.11	0.61
547.36224@10.5859995	547.3622	10.59	RP	POS			44890	8827	66843	31004	0.05425	0.84	1.00	616381	114125	723332	163219	0.12024	0.70	1.28
547.4974@13.997004	547.4974	14.00	RP	POS			2662448	162663	2668040	76645	0.93025	0.05	0.98	1804614	137361	1825102	224450	0.81456	0.11	0.55
549.28613@1.9859998	549.2861	1.99	HILIC	POS										30455	4818	29536	4337	0.67976	-0.20	0.45
549.30133@10.576997	549.3013	10.58	RP	NEG			71283	11605	83812	18311	0.09528	0.74	0.99	73150	10378	70839	11493	0.65998	-0.21	0.70
549.3797@1.1209997	549.3797	1.12	HILIC	POS			5727	1069	7687	1993	0.01691	1.03	1.10	84123	16858	93288	19912	0.30447	0.48	0.98
549.3799@1.2519999	549.3799	1.25	HILIC	POS	1-O-(cis-9-Octadecenyl)-2-O-acetyl-sn-glycero-3-phosphocholine	2	28982	6232	35993	9002	0.06755	0.81	0.97	498339	86368	562378	99348	0.16118	0.65	1.20
551.39484@1.2340002	551.3948	1.23	HILIC	POS			49192	10003	53398	14841	0.48355	0.32	0.91	414217	40788	388244	82034	0.39505	-0.38	0.80
551.3949@1.104	551.3949	1.10	HILIC	POS			6617	1685	8163	2831	0.16909	0.62	0.89	65046	8207	62739	15108	0.68530	-0.18	0.72
553.316@9.924997	553.3160	9.92	RP	NEG			57466	6898	65541	8809	0.04270	0.90	1.05	1381051	173330	1403611	382642	0.87018	0.07	0.39
553.37476@10.679995	553.3748	10.68	RP	NEG			31994	3713	33279	4102	0.49530	0.32	1.05	187542	7960	186478	17112	0.86388	-0.08	0.59
553.47046@12.109003	553.4705	12.11	RP	POS			136094	66815	96850	39686	0.16731	-0.69	0.86	2748	954	3162	656	0.31389	0.50	0.95
553.5434@10.281001	553.5434	10.28	RP	POS										187485	43246	148299	35008	0.05523	-0.90	1.92
555.3078@1.1959994	555.3078	1.20	HILIC	NEG			39147	8904	37002	7855	0.60004	-0.26	0.67	35981	4836	36808	5464	0.73756	0.16	0.52
555.308@9.941996	555.3080	9.94	RP	NEG			86657	17995	96978	8852	0.16550	0.70	1.04	109512	14210	120691	18732	0.16730	0.63	1.06
555.31195@9.933004	555.3120	9.93	RP	NEG			98006	18386	109439	10416	0.14266	0.73	1.06	179832	20680	191653	36507	0.39952	0.38	0.70
555.3486@1.1919999	555.3486	1.19	HILIC	NEG										34987	5886	34999	6512	0.99669	0.00	0.66
555.3504@1.3359996	555.3504	1.34	HILIC	NEG			24449	4850	27052	5622	0.30589	0.48	0.94	141769	27911	137105	30943	0.74108	-0.16	0.73
557.17114@6.4040003	557.1711	6.40	HILIC	NEG			36617	4381	48168	26467	0.20276	0.56	0.77							
557.2824@10.079996	557.2824	10.08	RP	POS			31918	7519	47818	20173	0.03823	0.90	0.99	474274	45735	474892	32687	0.97474	0.02	0.66
557.3195@1.1739999	557.3195	1.17	HILIC	NEG			37603	6083	36785	5829	0.77639	-0.14	0.67	74666	9761	75868	10643	0.80609	0.12	0.45
557.3215@1.3160007	557.3215	1.32	HILIC	NEG			74091	6027	77499	4590	0.20653	0.61	0.73	26386	3799	28117	3736	0.34655	0.45	0.80
557.32623@1.4960002	557.3262	1.50	HILIC	NEG										37589	7011	38806	5114	0.68695	0.20	0.59
557.33325@10.449003	557.3333	10.45	RP	NEG			98449	9538	114754	17812	0.02455	0.97	1.06	621390	37824	607533	72143	0.60759	-0.23	0.64
558.14264@2.5639997	558.1426	2.56	HILIC	NEG			188064	78783	197100	141312	0.86587	0.08	1.27							
558.14386@2.5740001	558.1439	2.57	HILIC	POS			633700	225590	578984	295097	0.66112	-0.20	1.50							
559.07117@7.690001	559.0712	7.69	HILIC	NEG	Adenosine diphosphoribose	2	98172	15881	105352	22161	0.43453	0.36	0.64							
559.07166@7.7010026	559.0717	7.70	HILIC	POS			51133	38402	74666	46560	0.25531	0.53	1.04							
559.3358@1.1539996	559.3358	1.15	HILIC	NEG			16866	2722	18315	3861	0.36370	0.42	0.88	55056	7011	52622	10094	0.55469	-0.27	0.68
559.33716@1.2859995	559.3372	1.29	HILIC	NEG			88725	3626	84936	4550	0.06494	-0.83	2.10	25129	3304	25318	5086	0.92526	0.04	0.42
560.20996@6.150001	560.2100	6.15	HILIC	NEG			67378	23330	63836	35162	0.80104	-0.12	1.25							
563.32104@9.696005	563.3210	9.70	RP	NEG	LysoPC 18:2 (M+FA-H-)	1	13763	1775	13047	1650	0.39348	-0.41	1.60	140562	47133	121165	49773	0.40886	-0.39	0.74
564.36774@10.454003	564.3677	10.45	RP	NEG			216658	44192	250902	35184	0.09434	0.80	0.92	88853	22590	66984	21729	0.05357	-0.89	1.59
565.3121@9.931	565.3121	9.93	RP	POS			94337	17963	104212	20641	0.29263	0.49	0.86	1186771	134396	1115113	171012	0.33273	-0.45	0.88
565.3161@1.2669995	565.3161	1.27	HILIC	POS			12352	1828	15818	3545	0.01672	1.03	1.14	39832	2182	38470	2643	0.24689	-0.54	0.98
565.3367@1.347	565.3367	1.35	HILIC	NEG			90869	13819	118941	23530	0.00592	1.16	1.25	1789567	249944	1796824	234161	0.95056	0.03	0.56
565.33673@1.2029994	565.3367	1.20	HILIC	NEG			106760	23749	107595	24743	0.94283	0.03	0.70	446267	60540	453641	52868	0.78949	0.13	0.52
565.3488@11.088996	565.3488	11.09	RP	NEG			31863	31403	31579	30560	0.98481	-0.01	1.19	67235	30881	87298	42334	0.26079	0.52	0.91
565.4126@1.2120006	565.4126	1.21	HILIC	POS			4972	1316	5435	1927	0.55381	0.27	0.73	40150	5818	38722	8656	0.68160	-0.19	0.71
566.0542@8.054998	566.0542	8.05	HILIC	NEG			49062	6847	61390	23751	0.14414	0.64	0.93							

567.25244@12.437995	567.2524	12.44	RP	NEG			16116	6351	15205	6444	0.76737	-0.14	0.87	114920	16838	113279	16277	0.83727	-0.10	0.74
567.3326@9.925997	567.3326	9.93	RP	POS			64062	11298	71840	12788	0.18815	0.61	0.94	1581307	168341	1545419	264949	0.73118	-0.16	0.46
567.3329@10.063997	567.3329	10.06	RP	POS	LysoPC 22:6	1	88258	15500	129851	45326	0.01804	1.02	1.09	5856859	639708	5641270	722418	0.51105	-0.31	0.58
567.3332@10.0650015	567.3332	10.07	RP	POS			85733	13514	130714	46187	0.01259	1.07	1.14	5693500	568524	5466043	649555	0.43907	-0.36	0.65
567.35175@1.1689998	567.3518	1.17	HILIC	NEG			54585	8055	56882	14394	0.67436	0.19	0.88	489938	89873	473241	67308	0.66887	-0.21	0.67
567.3525@1.3129998	567.3525	1.31	HILIC	NEG			136410	14936	155362	27825	0.08344	0.76	1.00	1861605	331953	1800199	245435	0.66904	-0.21	0.70
569.2721@1.9229996	569.2721	1.92	HILIC	NEG			7512	1653	10100	2709	0.02322	0.98	1.08	56716	8018	47321	7868	0.02339	-1.02	1.72
569.34784@10.178995	569.3478	10.18	RP	POS			10805	2139	12818	3642	0.16265	0.63	1.03	248303	35384	227375	27528	0.19009	-0.63	1.03
569.3483@10.362	569.3483	10.36	RP	POS			29638	5968	36223	5566	0.02866	0.99	1.15	234251	48133	218927	38783	0.47617	-0.35	0.59
569.3678@1.153	569.3678	1.15	HILIC	NEG			52268	11333	61816	21658	0.24667	0.52	0.98	549733	86925	514330	105815	0.44597	-0.36	0.87
569.3683@1.2869996	569.3683	1.29	HILIC	NEG			399452	67549	422360	93155	0.55341	0.27	1.03	2045958	337099	1907329	392618	0.43109	-0.37	0.93
571.2273@5.7400017	571.2273	5.74	HILIC	POS			16730	2076	17683	1979	0.33751	0.46	0.94	16260	1495	16760	2061	0.55861	0.27	0.53
571.2291@5.602998	571.2291	5.60	HILIC	POS			22712	8011	28217	6842	0.14233	0.70	0.77	10495	1788	11471	1744	0.26059	0.53	1.05
572.4183@10.449003	572.4183	10.45	RP	NEG			55692	12417	67314	10940	0.05396	0.89	0.98	24779	7378	19181	10086	0.19122	-0.60	1.20
573.2415@1.4479992	573.2415	1.45	HILIC	NEG			2712	1995	4327	2022	0.10765	0.75	1.11	20532	3100	20457	3016	0.95927	-0.02	0.47
573.24866@2.188	573.2487	2.19	HILIC	NEG			67226	60520	82574	30720	0.52681	0.33	0.57							
574.1181@2.5740001	574.1181	2.57	HILIC	POS			78492	25410	71739	31130	0.61855	-0.23	1.57							
575.2565@1.4360006	575.2565	1.44	HILIC	NEG			14331	2504	14207	2326	0.91511	-0.05	1.45	140946	22415	144336	23249	0.75753	0.15	0.48
575.26794@10.402006	575.2679	10.40	RP	POS			15143	2221	15706	4915	0.75100	0.14	1.19	182460	21519	183064	25241	0.95689	0.03	0.66
575.5279@14.002007	575.5279	14.00	RP	POS			375972	36535	379378	24361	0.82428	0.11	1.00	143893	21130	156942	39139	0.38013	0.40	0.81
576.4499@10.730002	576.4499	10.73	RP	NEG			81431	11804	93906	14361	0.05825	0.85	1.05	80219	21582	72410	29667	0.52666	-0.29	0.60
577.2558@1.4360006	577.2558	1.44	HILIC	NEG			14150	2729	13617	2397	0.67006	-0.21	1.48	134140	20887	136691	21512	0.80241	0.12	0.45
577.2819@12.438997	577.2819	12.44	RP	NEG			4252	1145	3679	992	0.28019	-0.52	0.67	399319	57782	368167	81509	0.35626	-0.42	0.96
577.4749@10.731002	577.4749	10.73	RP	POS			38615	7775	36647	8165	0.60829	-0.24	0.85							
579.3704@10.485	579.3704	10.49	RP	NEG			40307	13934	56340	18042	0.04753	0.88	1.04							
579.4267@1.1839994	579.4267	1.18	HILIC	POS			13280	3074	17263	6148	0.09353	0.74	0.96	95503	7201	96631	13657	0.82491	0.10	0.49
580.4793@10.795005	580.4793	10.80	RP	NEG			62368	10874	70326	10625	0.13755	0.70	0.88	9576	2888	6776	2651	0.04959	-0.91	1.65
583.24286@1.8810003	583.2429	1.88	HILIC	NEG										37616	4742	33822	6110	0.15534	-0.65	1.12
583.27765@2.187	583.2777	2.19	HILIC	NEG			91485	87189	117176	54895	0.48074	0.35	0.49							
583.38086@10.5859995	583.3809	10.59	RP	NEG			2798834	490933	3492807	557261	0.01177	1.10	1.18	1016178	271141	760555	187292	0.04005	-0.98	1.71
584.2636@6.8049994	584.2636	6.80	RP	POS										46042	19206	48293	21169	0.81613	0.11	0.81
585.3626@1.3860005	585.3626	1.39	HILIC	NEG			284766	44269	297732	47123	0.55618	0.28	1.10	731979	179670	799765	111158	0.36788	0.45	1.01
587.1022@6.8900003	587.1022	6.89	HILIC	NEG			27095	15908	43993	46667	0.30443	0.45	1.05							
587.31915@10.112	587.3192	10.11	RP	NEG			93491	12160	103843	16915	0.14899	0.65	1.12	273762	14896	284404	18630	0.19476	0.60	1.08
587.3211@1.3069998	587.3211	1.31	HILIC	NEG										27880	8328	32361	6124	0.22382	0.59	1.16
587.3214@9.8099985	587.3214	9.81	RP	NEG	LysoPC 20:5 (M+FA-H-)	2	9030	1725	11252	2479	0.03837	0.91	1.09	605846	146688	664060	113703	0.37108	0.44	0.89
588.4695@10.729003	588.4695	10.73	RP	NEG			43698	6851	48331	6395	0.16074	0.66	0.87	24237	5686	19697	4542	0.08590	-0.82	1.45
589.33624@1.2659998	589.3362	1.27	HILIC	NEG			35448	4844	46961	10839	0.00917	1.11	1.24	990846	199029	933572	119297	0.48642	-0.35	0.82
589.3367@1.1310004	589.3367	1.13	HILIC	NEG			27216	4591	26196	6268	0.69525	-0.18	0.77	267053	55353	247270	30832	0.38356	-0.44	0.91
589.37494@10.446998	589.3749	10.45	RP	NEG			90143	17127	109111	16568	0.03004	0.98	1.09	16953	6367	10084	5574	0.02876	-1.00	1.69
589.90436@4.362	589.9044	4.36	HILIC	NEG			165716	3899	165582	8240	0.96417	-0.02	0.74	192152	16411	193490	20750	0.88029	0.07	0.55
591.35016@1.2760007	591.3502	1.28	HILIC	NEG			15799	3386	20593	4164	0.01485	1.06	1.20	351423	97956	376974	63611	0.53447	0.31	0.71
591.3504@1.1349996	591.3504	1.13	HILIC	NEG			11156	2001	11520	2873	0.75562	0.14	0.73	90614	24583	97294	15386	0.51437	0.33	0.73
591.35095@10.128999	591.3510	10.13	RP	NEG			104415	22950	114339	12210	0.29189	0.54	0.86	368124	92048	576323	324215	0.07691	0.77	1.27
591.37177@10.449003	591.3718	10.45	RP	NEG			1851482	373504	2234777	358386	0.04186	0.93	1.05	332571	137144	225289	84718	0.07539	-0.87	1.58
591.4166@10.462999	591.4166	10.46	RP	POS			231119	38373	250152	24249	0.24387	0.58	0.68							
592.35596@10.240999	592.3560	10.24	RP	NEG										326804	64727	356873	74051	0.37067	0.42	0.87
593.27203@1.8840001	593.2720	1.88	HILIC	NEG										64203	9529	53716	9453	0.03243	-0.97	1.62
593.3654@10.371001	593.3654	10.37	RP	NEG			214323	97787	386536	162724	0.01300	1.06	1.16	177647	23542	173579	18928	0.69705	-0.19	0.74
593.40674@10.730002	593.4067	10.73	RP	NEG			149741	16982	166318	16254	0.05140	0.89	1.08	63367	12479	55661	8556	0.15931	-0.69	1.38
595.364@1.2040002	595.3640	1.20	HILIC	POS										22428	4850	25970	5333	0.15832	0.65	1.19
595.3831@1.2449999	595.3831	1.24	HILIC	NEG			4452	809	5074	1284	0.22714	0.55	0.92	44857	12728	47950	8730	0.56758	0.29	0.72
595.38513@10.587004	595.3851	10.59	RP	NEG			82621	12779	103752	15078	0.00480	1.20	1.29	68331	14008	67954	15258	0.95705	-0.03	0.77
595.40247@10.730002	595.4025	10.73	RP	NEG			3053321	330509	3453507	355305	0.02406	1.01	1.14	1315211	257763	1156338	161230	0.15249	-0.71	1.40
595.9703@9.321005	595.9703	9.32	RP	NEG			188213	5040	184868	3628	0.13667	-0.72	1.57	193467	12436	190196	11977	0.58048	-0.27	0.76
597.5703@10.591002	597.5703	10.59	RP	POS			3110	1442	3946	3192	0.47185	0.32	0.90	93642	36177	99677	44483	0.75461	0.15	0.49
598.42804@10.588004	598.4280	10.59	RP	POS			190943	24871	221232	25791	0.02153	1.03	1.12	66264	46538	56756	41750	0.65815	-0.22	0.59
598.4969@2.8450007	598.4969	2.85	HILIC	POS			109683	29299	136782	35127	0.09130	0.77	0.91	241204	29442	204694	36847	0.03121	-0.95	1.65
599.4319@10.793	599.4319	10.79	RP	NEG			160158	18631	180656	17706	0.03002	0.99	1.09	23174	6821	19228	4747	0.18681	-0.65	1.31
600.2584@1.9179996	600.2584	1.92	HILIC	POS										73143	30904	75886	22883	0.83716	0.10	0.93
600.5098@2.9660015	600.5098	2.97	HILIC	POS			33094	9219	42182	11193	0.07518	0.80	1.15	80610	10403	78825	15283	0.77220	-0.13	0.33
601.21@10.005003	601.2100	10.01	RP	NEG			5259	1008	7877	3386	0.03850	0.90	1.00	46651	6643	38400	6865	0.01927	-1.04	1.69
601.26294@1.4490004	601.2629	1.45	HILIC	NEG			643	611	1341	755	0.08413	0.89	0.99	35790	5573	38077	4832	0.37266	0.43	0.78

601.34155@1.2760007	601.3416	1.28	HILIC	POS			50833	4644	57515	18771	0.30014	0.46	0.64	4098	1148	4025	1010	0.88925	-0.07	0.42
602.45935@10.811998	602.4594	10.81	RP	POS			125163	13980	141439	23278	0.08419	0.77	0.87							
603.283@1.4440001	603.2830	1.44	HILIC	NEG			2378	427	2172	527	0.37259	-0.42	1.44	23976	4289	23438	3644	0.78104	-0.14	0.60
603.3084@1.102	603.3084	1.10	HILIC	NEG										26147	4520	25521	3317	0.74816	-0.16	0.53
603.37146@10.446005	603.3715	10.45	RP	NEG			45230	7184	51935	7293	0.06705	0.84	1.05	21175	4686	17780	6438	0.21223	-0.57	1.18
604.5638@10.648996	604.5638	10.65	HILIC	POS			30583	2364	30941	3413	0.79630	0.12	1.25	55500	6297	55213	6206	0.92415	-0.05	0.64
606.41205@11.200996	606.4121	11.20	RP	NEG			27164	3698	31354	8679	0.18948	0.58	0.94	139978	30840	131959	28425	0.57805	-0.27	0.66
607.0807@1.5079998	607.0807	1.51	RP	NEG	Uridine 5'-diphospho-N-acetylgalactosamine	2	1896154	281430	2170034	642624	0.24569	0.52	0.71							
607.0808@7.822	607.0808	7.82	HILIC	NEG	Uridine 5'-diphospho-N-acetylgalactosamine	2	60035	10254	75477	31822	0.17341	0.60	0.97							
607.3094@9.902006	607.3094	9.90	RP	NEG			17439	4435	18062	2644	0.73213	0.17	1.02	127568	18338	117721	9934	0.19743	-0.65	1.08
607.3678@10.449003	607.3678	10.45	RP	NEG			107353	22965	124043	15660	0.10182	0.79	0.97	19534	5627	14236	5312	0.05810	-0.87	1.49
607.4025@10.722994	607.4025	10.72	RP	NEG			87469	7184	96065	8407	0.03119	0.96	1.14	84707	10418	75892	10483	0.09295	-0.78	1.46
607.4581@1.1430004	607.4581	1.14	HILIC	POS			11712	3305	13367	4027	0.35105	0.43	0.95	81104	7548	78680	13255	0.63215	-0.22	0.58
608.37213@10.453002	608.3721	10.45	RP	NEG			84979	15693	97075	9628	0.07894	0.86	0.95	29125	8111	21978	6824	0.06420	-0.87	1.59
609.17725@11.928003	609.1773	11.93	RP	POS			158575	16993	153866	12063	0.51896	-0.32	0.86	154498	16846	191754	52684	0.05612	0.83	1.38
609.32416@10.265998	609.3242	10.27	RP	NEG			516230	43698	550088	73498	0.24193	0.53	0.92	700673	63755	701646	54157	0.97301	0.02	0.59
609.325@1.3870002	609.3250	1.39	HILIC	NEG			65060	5606	61255	6506	0.19959	-0.59	1.90	19528	8886	14455	9902	0.26795	-0.52	0.97
609.36035@1.361	609.3604	1.36	HILIC	NEG			60212	14781	77985	14795	0.02131	1.03	1.15	122080	31338	154214	23587	0.02997	1.01	1.65
609.53436@13.635994	609.5344	13.64	RP	POS	DG 16:0_18:2	2	412530	209222	254654	142310	0.09041	-0.82	0.94	130057	30196	105098	59681	0.26720	-0.50	0.89
610.3732@10.454003	610.3732	10.45	RP	NEG			199060	41204	230211	31639	0.09871	0.79	0.91	76787	19206	59296	16416	0.05776	-0.89	1.59
610.4361@10.449003	610.4361	10.45	RP	NEG			75680	16777	84749	13678	0.23534	0.57	0.77	14024	5651	10750	4091	0.19029	-0.64	1.39
611.2536@9.890999	611.2536	9.89	RP	NEG			7863	1562	7289	1346	0.42243	-0.39	1.00	88262	11780	85811	6923	0.61178	-0.26	0.70
611.37714@1.3440007	611.3771	1.34	HILIC	NEG			42343	11078	46645	9274	0.39257	0.42	0.72	61526	16796	68901	10112	0.29482	0.53	0.98
611.3994@10.729003	611.3994	10.73	RP	NEG			101768	9953	110565	7285	0.05488	0.91	1.13	32563	5997	29454	5192	0.26342	-0.54	1.15
612.15125@1.191	612.1513	1.19	RP	NEG			340319	111734	325813	103435	0.78082	-0.13	0.93							
612.15137@8.501001	612.1514	8.50	HILIC	NEG	Oxidized glutathione	2	59806	14391	64052	22352	0.63207	0.22	1.12							
612.3714@10.453002	612.3714	10.45	RP	NEG			92534	18440	104396	14943	0.16106	0.67	0.85	38155	9977	31191	8044	0.12990	-0.72	1.38
612.4023@10.729003	612.4023	10.73	RP	NEG			88860	7969	94582	4610	0.09608	0.82	1.00	32806	6011	31035	4023	0.48676	-0.35	1.05
613.2673@10.260003	613.2673	10.26	RP	NEG			235346	22642	243402	32846	0.54684	0.28	0.91	323488	30077	321114	21158	0.85287	-0.09	0.67
613.337@1.113	613.3370	1.11	HILIC	NEG			4409	665	4896	1164	0.28124	0.49	0.73	154709	35317	143945	20574	0.45909	-0.38	0.83
613.33746@10.035003	613.3375	10.04	RP	NEG	LysoPC 22:6 (M+FA-H-)	2	67888	11948	83454	22237	0.07603	0.78	1.06	2824169	304614	2731322	388254	0.57672	-0.26	0.47
613.34015@1.2439996	613.3402	1.24	HILIC	NEG			15335	3671	18685	4162	0.08683	0.78	0.86	595670	136148	563174	87292	0.56815	-0.29	0.76
613.39307@1.3409997	613.3931	1.34	HILIC	NEG			85960	21705	89039	20617	0.76355	0.15	0.97	55821	15450	58134	9978	0.71976	0.18	0.75
614.40424@10.730002	614.4042	10.73	RP	NEG			203203	18536	213440	17472	0.24908	0.55	0.87	111417	11865	99194	12499	0.04852	-0.90	1.58
615.16925@7.7660003	615.1693	7.77	RP	POS			322107	165401	296913	83577	0.70197	-0.20	1.22	1518	608	2931	4229	0.32014	0.44	0.71
615.1699@0.8199999	615.1699	0.82	HILIC	POS			462661	136525	469320	152071	0.92324	0.05	0.81							
615.3285@10.169004	615.3285	10.17	RP	NEG			7796	1837	9643	3240	0.14683	0.65	0.97	149821	18920	143490	24168	0.54085	-0.29	0.55
615.3695@10.436001	615.3695	10.44	RP	NEG			42795	10906	56881	11925	0.01783	1.05	1.12							
618.3953@10.587004	618.3953	10.59	RP	NEG			133751	18280	158424	21386	0.01677	1.05	1.12	59967	10175	49292	12307	0.05919	-0.85	1.54
618.5228@13.830996	618.5228	13.83	RP	POS			189167	82671	132759	74978	0.15301	-0.68	0.81	136381	25765	106888	36545	0.06056	-0.83	1.40
619.39844@10.638	619.3984	10.64	RP	NEG			84600	19685	107010	26134	0.05274	0.86	1.02							
620.29517@12.438998	620.2952	12.44	RP	NEG										82862	31193	89368	61111	0.77383	0.13	0.44
620.2952@12.019997	620.2952	12.02	RP	NEG			632632	95473	754110	143762	0.04627	0.88	1.12	293359	62790	295263	63745	0.95011	0.03	0.55
621.3106@1.134	621.3106	1.13	HILIC	POS			13222	2793	12262	2865	0.48319	-0.33	0.82	35409	3973	34525	5555	0.69887	-0.18	0.47
624.45013@10.5859995	624.4501	10.59	RP	NEG			67583	12536	87185	17667	0.01333	1.07	1.15	62193	17063	46417	20985	0.09557	-0.76	1.29
624.5126@2.7229998	624.5126	2.72	HILIC	POS			225099	58114	276127	67940	0.10331	0.74	1.04	410677	65588	379030	75472	0.35427	-0.43	0.76
625.0721@5.330998	625.0721	5.33	HILIC	NEG			39598	1518	42104	2686	0.02373	0.98	1.31	1969	337	2559	978	0.09871	0.72	1.18
625.20953@9.996001	625.2095	10.00	RP	NEG			4108	1292	6588	1971	0.00503	1.18	1.30	67837	10424	55265	9874	0.01876	-1.06	1.74
625.2683@9.882002	625.2683	9.88	RP	NEG										100747	13239	93726	9514	0.22758	-0.59	1.02
626.52783@2.7290015	626.5278	2.73	HILIC	POS			316923	84473	363613	81181	0.25227	0.54	0.93	872256	91983	785890	149503	0.15070	-0.64	1.13
627.28204@10.260003	627.2820	10.26	RP	NEG			292467	28237	301330	39879	0.58850	0.25	1.00	263343	22606	258894	11274	0.62105	-0.25	0.69
628.48193@10.795005	628.4819	10.80	RP	NEG			116156	16691	132491	20998	0.08249	0.78	1.05	75503	23543	64275	31490	0.39842	-0.39	1.00
628.5053@12.212004	628.5053	12.21	RP	NEG			39043	14500	54666	15059	0.03897	0.93	1.09	166843	44736	159730	52680	0.76038	-0.14	0.43
629.3631@10.449003	629.3631	10.45	RP	NEG			65148	7293	71993	3953	0.03472	1.03	1.14	8423	2728	6264	2728	0.11352	-0.74	1.23
629.5069@10.800999	629.5069	10.80	RP	POS			95061	18579	92756	15921	0.78419	-0.13	0.47							
630.11536@1.7619997	630.1154	1.76	HILIC	POS			21297	9925	24108	14054	0.62565	0.23	1.18							
630.5211@12.285003	630.5211	12.29	RP	NEG			17275	10759	42937	19175	0.00242	1.26	1.61	123666	43323	186401	55423	0.01480	1.06	1.77
631.1194@6.627	631.1194	6.63	HILIC	NEG			377475	76159	432170	80087	0.15677	0.66	1.02							
631.4@10.558004	631.4000	10.56	RP	NEG			511866	137244	698263	173203	0.02021	1.02	1.11	245370	56941	182798	66683	0.04579	-0.90	1.48
631.4375@10.791006	631.4375	10.79	RP	NEG			62644	4146	65713	4946	0.16942	0.63	0.83	11955	2521	10987	5484	0.62712	-0.22	0.80
632.5121@11.058005	632.5121	11.06	RP	NEG			18976	4368	25750	8221	0.03980	0.90	1.07	52126	32102	44465	16096	0.55033	-0.31	0.72
633.3243@9.944	633.3243	9.94	RP	NEG			116304	21832	128793	9308	0.16058	0.72	0.99	127165	12624	138393	15447	0.10695	0.73	1.23
633.32434@10.112	633.3243	10.11	RP	NEG			66478	8886	80743	14785	0.02161	0.99	1.12	250406	15484	256088	19258	0.49659	0.32	0.69

635.3404@10.444	635.3404	10.44	RP	NEG			180855	15852	193326	28649	0.25872	0.51	1.14	763881	68150	738566	88690	0.50230	-0.31	0.75
635.4324@10.833006	635.4324	10.83	RP	NEG			99970	32658	136015	43975	0.06159	0.83	1.03	25163	8493	19886	6183	0.16268	-0.68	1.44
635.4328@10.832	635.4328	10.83	RP	NEG			425479	73146	491727	67027	0.06455	0.86	0.99	40670	12269	32760	9671	0.15687	-0.68	1.38
635.5497@13.829996	635.5497	13.83	RP	POS	DG 18:1_18:2	2	242266	114989	163944	93479	0.13966	-0.71	0.84	306156	61009	233427	92527	0.06127	-0.83	1.40
636.1487@6.627	636.1487	6.63	HILIC	NEG			66272	9022	65816	5467	0.90201	-0.06	0.45							
637.26685@10.071999	637.2669	10.07	RP	NEG			14232	2527	21039	4421	0.00079	1.36	1.63	174386	13338	179573	15489	0.45553	0.35	0.85
637.35565@10.729003	637.3557	10.73	RP	NEG			545689	72206	593377	127039	0.33098	0.44	1.00	1617988	253850	1526059	277553	0.47358	-0.34	0.74
639.2852@10.403998	639.2852	10.40	RP	NEG			88491	11743	95193	16542	0.32904	0.45	1.10	392986	31214	398574	38490	0.73775	0.16	0.88
639.39154@10.449003	639.3915	10.45	RP	NEG			224517	51151	263837	32391	0.08118	0.85	0.92	27958	11025	19826	7257	0.09505	-0.81	1.50
639.42554@10.730002	639.4255	10.73	RP	NEG			50279	3551	50078	3658	0.90762	-0.06	0.95	47113	5583	43820	4077	0.18401	-0.65	1.26
641.265@10.261995	641.2650	10.26	RP	NEG			220402	19264	227223	30324	0.56951	0.26	1.09	306240	33436	311314	35498	0.75927	0.15	0.72
641.4075@10.5859995	641.4075	10.59	RP	NEG			139657	23239	171719	22995	0.00948	1.14	1.22	49436	11873	38038	9022	0.04067	-0.96	1.70
643.4025@10.5859995	643.4025	10.59	RP	NEG			2768147	479624	3449955	542397	0.01125	1.11	1.19	976490	263379	722792	186342	0.03731	-0.99	1.71
643.4216@10.727998	643.4216	10.73	RP	NEG			262265	27188	281034	17021	0.11259	0.78	0.89	84093	14522	76542	11020	0.24295	-0.57	1.23
645.4089@10.7109995	645.4089	10.71	RP	NEG			121954	33424	165763	33127	0.01295	1.10	1.18	75077	13272	65754	17464	0.21461	-0.57	1.22
645.96716@9.709999	645.9672	9.71	RP	NEG			63642	1535	64275	2220	0.48511	0.32	0.77	75108	9638	73709	10025	0.76720	-0.14	0.74
646.45233@11.480003	646.4523	11.48	RP	POS			309223	43901	322098	48264	0.56168	0.28	0.98	237834	69603	305104	146417	0.21904	0.55	1.12
646.51483@11.640997	646.5148	11.64	RP	NEG			18488	5384	24089	7121	0.07372	0.80	0.90	243331	66527	228797	60086	0.63713	-0.23	0.43
647.43335@10.791994	647.4334	10.79	RP	NEG			3664333	462918	4155894	445533	0.03618	0.95	1.06	416984	161387	310670	85253	0.11895	-0.78	1.50
647.4797@10.800002	647.4797	10.80	RP	POS			120308	27769	117844	22199	0.84101	-0.10	0.41							
648.0709@6.6470017	648.0709	6.65	HILIC	NEG			54531	22527	66718	18448	0.23560	0.57	0.92							
648.3843@10.556	648.3843	10.56	RP	NEG			28526	5622	36331	5527	0.00898	1.15	1.24	39270	8242	31471	7636	0.05544	-0.88	1.45
649.4685@11.058005	649.4685	11.06	RP	NEG			31710	8552	44318	14307	0.03345	0.93	1.05	26420	4054	26604	5310	0.93447	0.04	0.94
650.384@10.556	650.3840	10.56	RP	NEG			46933	8687	56649	7530	0.02374	1.03	1.13	61011	15618	48232	21425	0.16111	-0.64	1.08
650.44946@10.5859995	650.4495	10.59	RP	NEG			55671	9459	66074	10100	0.03755	0.94	1.05	33130	6343	28806	5706	0.15218	-0.68	1.43
651.2848@9.942995	651.2848	9.94	RP	NEG			98400	18594	110976	9817	0.11035	0.80	1.06	107612	11826	116633	17824	0.21549	0.56	1.00
651.4646@11.058005	651.4646	11.06	RP	NEG			567272	165823	805168	276610	0.03749	0.91	1.05	522860	62210	498537	100775	0.53826	-0.28	0.94
652.2958@10.394998	652.2958	10.39	RP	NEG			33650	5088	37820	8890	0.22898	0.54	0.86	469738	35025	458440	45419	0.55878	-0.27	0.78
653.40894@10.730002	653.4089	10.73	RP	NEG			75081	5474	80552	6996	0.07915	0.79	1.10	71794	6654	66169	9126	0.14837	-0.66	1.21
654.14594@6.6159987	654.1459	6.62	HILIC	NEG			190715	39932	188554	33452	0.90407	-0.06	0.87							
655.3137@10.760003	655.3137	10.76	RP	NEG			38709	5223	36491	5713	0.40207	-0.40	1.46	126249	19061	124624	26083	0.88045	-0.07	0.53
655.3138@10.694	655.3138	10.69	RP	NEG			53702	21798	59541	26776	0.61623	0.24	0.98	457552	44914	442481	55425	0.53202	-0.29	0.70
657.40125@10.587004	657.4013	10.59	RP	NEG			101905	12889	118951	14123	0.01572	1.06	1.14	59053	8645	46331	9987	0.00970	-1.12	1.85
657.89197@4.4069986	657.8920	4.41	HILIC	NEG			103008	3126	103613	5112	0.76090	0.14	0.77	109576	9624	111855	11900	0.65853	0.21	0.66
659.39777@10.5859995	659.3978	10.59	RP	NEG			196082	33595	241933	33509	0.01033	1.13	1.23	87422	15189	69671	16091	0.02779	-0.99	1.65
660.40393@10.587004	660.4039	10.59	RP	NEG			110490	14419	126665	12088	0.02223	1.05	1.14	49817	10216	39710	6984	0.03212	-1.02	1.77
661.1737@7.7650003	661.1737	7.77	RP	NEG			89994	16741	98121	21887	0.38396	0.40	0.84							
662.40466@10.587004	662.4047	10.59	RP	NEG			309454	38967	359840	41353	0.01645	1.06	1.13	165405	28812	131394	32548	0.03060	-0.96	1.65
662.44763@12.716002	662.4476	12.72	RP	POS			1307051	332106	1230744	181437	0.57034	-0.29	0.89	1129891	296137	1156262	395437	0.87336	0.07	0.42
662.46497@10.5859995	662.4650	10.59	RP	NEG			61871	10670	72627	10454	0.04676	0.91	0.99	27609	6455	21783	5444	0.05881	-0.88	1.62
663.43115@10.800997	663.4312	10.80	RP	NEG			232789	31202	255853	23941	0.10569	0.78	1.03	21721	5132	18361	4243	0.15658	-0.68	1.25
664.43475@10.802001	664.4348	10.80	RP	NEG			140660	14712	151979	11294	0.09341	0.80	0.96	71116	12928	63849	11254	0.22776	-0.58	1.19
665.26117@10.121001	665.2612	10.12	RP	NEG			13341	2181	18025	4288	0.00868	1.11	1.33	145581	17257	149138	19188	0.68425	0.19	0.69
666.43646@10.795005	666.4365	10.80	RP	NEG			374264	39211	407360	37921	0.08884	0.79	0.97	184484	24535	165807	22758	0.11635	-0.74	1.47
667.14844@6.413001	667.1484	6.41	HILIC	NEG			13855	3971	11905	2809	0.26020	-0.55	1.20							
667.4019@10.566996	667.4019	10.57	RP	NEG			189770	40085	245904	44142	0.01142	1.11	1.18	155965	29965	118242	36640	0.02728	-0.98	1.60
668.08563@6.627	668.0856	6.63	HILIC	NEG			26792	4387	27524	3099	0.69594	0.20	0.59							
668.43365@10.794004	668.4337	10.79	RP	NEG			168434	14959	180143	16501	0.13273	0.69	0.94	80419	12240	71121	10125	0.10403	-0.77	1.47
668.4658@11.058005	668.4658	11.06	RP	NEG			35193	6612	43035	8891	0.04595	0.89	1.02	28639	3217	27157	5557	0.48868	-0.31	0.79
669.29193@10.6900015	669.2919	10.69	RP	NEG			54017	8431	55763	13951	0.74706	0.15	1.00	317987	58171	312192	65186	0.84451	-0.09	0.65
669.41724@10.712004	669.4172	10.71	RP	NEG			64429	17250	86467	16215	0.01341	1.10	1.23	69661	13575	59355	15117	0.14578	-0.67	1.36
670.4669@11.058005	670.4669	11.06	RP	NEG			81900	14466	99238	22531	0.06428	0.82	1.01	59935	5603	58705	12804	0.78890	-0.12	0.24
671.09735@6.605002	671.0974	6.61	HILIC	NEG			56315	19518	61136	17276	0.59132	0.26	0.76							
671.39764@6.4110017	671.3976	6.41	HILIC	POS			11579	3765	10786	3118	0.63889	-0.23	0.42							
674.53357@12.126005	674.5336	12.13	RP	NEG			410457	109659	479929	162404	0.29494	0.48	0.97	95830	12565	99332	14478	0.58985	0.25	0.77
674.53625@11.979003	674.5363	11.98	RP	POS	SM 32:1	2	1128191	314504	1314535	458680	0.32173	0.45	0.86	297377	33426	263306	59576	0.14474	-0.65	1.16
676.12756@6.614999	676.1276	6.61	HILIC	NEG			27730	3015	26110	2501	0.24063	-0.57	0.90							
681.3975@10.5859995	681.3975	10.59	RP	NEG			124684	19873	147286	19396	0.02685	1.00	1.10	50947	6782	42547	8795	0.03451	-0.94	1.56
683.39484@10.567995	683.3948	10.57	RP	NEG			108926	17314	125569	14324	0.04461	0.94	1.04	44934	7581	36483	6791	0.02545	-1.02	1.71
683.4171@10.459998	683.4171	10.46	RP	NEG			31782	10954	37000	8151	0.28039	0.53	0.75	115840	21699	93179	21980	0.04264	-0.92	1.62
684.5114@10.475001	684.5114	10.48	RP	NEG										187465	37231	170452	26884	0.29582	-0.51	0.90
685.42676@10.791994	685.4268	10.79	RP	NEG			96868	6142	98959	4655	0.43813	0.38	0.79	10481	2818	9046	1813	0.23393	-0.59	0.96
686.4004@10.566996	686.4004	10.57	RP	NEG			74807	11351	89642	10915	0.01232	1.11	1.19	57161	9902	48548	10799	0.09550	-0.77	1.34

686.5351@12.033998	686.5351	12.03	RP	NEG			1477895	487472	1605009	466912	0.58280	0.26	0.97	813123	59575	757542	150049	0.30288	-0.46	0.81
687.4837@11.804	687.4837	11.80	RP	NEG	PE 16:1/16:1	2	157821	33211	151739	30692	0.69482	-0.19	0.57							
688.5502@12.239996	688.5502	12.24	RP	NEG			10590740	2276764	12588911	3421986	0.15629	0.64	0.82	329106	34891	361957	38390	0.07412	0.81	1.47
688.55273@12.139006	688.5527	12.14	RP	POS	SM 33:1	2	665161	150653	800074	264387	0.19195	0.59	0.90	161505	14418	161046	24541	0.96111	-0.02	0.71
689.4525@11.058005	689.4525	11.06	RP	NEG			35501	7297	44782	10219	0.03778	0.92	1.02	25653	6212	26489	6303	0.78145	0.13	0.97
689.4982@12.166	689.4982	12.17	RP	NEG	PE 16:0_16:1	2	189496	28711	156686	27358	0.02507	-1.01	1.41							
689.50085@12.171998	689.5009	12.17	RP	POS			141439	24341	128629	35009	0.37326	-0.41	0.81							
691.403@10.557005	691.4030	10.56	RP	NEG			102067	47577	167965	64278	0.02248	1.00	1.10	136539	46352	99069	42145	0.09469	-0.78	1.31
691.42194@10.5859995	691.4219	10.59	RP	NEG			312356	53928	382580	52669	0.01302	1.10	1.17	92679	22372	70136	19038	0.03780	-0.96	1.61
692.0189@6.199999	692.0189	6.20	HILIC	NEG										100156	27115	104366	53910	0.83241	0.10	0.55
695.45416@10.793	695.4542	10.79	RP	NEG			461444	64287	506942	43911	0.11035	0.78	0.90	22259	7234	16991	5774	0.11429	-0.75	1.40
695.9639@10.078002	695.9639	10.08	RP	NEG			92132	1774	94149	3508	0.13366	0.67	1.00	120906	16379	117491	13789	0.64404	-0.23	0.88
697.4536@10.822999	697.4536	10.82	RP	NEG			110252	25626	136777	21118	0.03205	0.99	1.10	24021	6315	18306	4073	0.04495	-0.96	1.71
699.086@6.4140005	699.0860	6.41	HILIC	NEG			30704	8768	28197	8645	0.55210	-0.29	1.10							
699.4858@11.058005	699.4858	11.06	RP	NEG			85279	18845	112333	28652	0.02754	0.96	1.07	74737	13055	70465	16196	0.54324	-0.28	0.82
699.5602@11.765997	699.5602	11.77	RP	POS			67454	16862	70205	14871	0.72186	0.17	0.83	80950	9151	84814	20558	0.60276	0.23	0.39
699.5626@11.763	699.5626	11.76	RP	NEG			48673	14861	46305	10117	0.70621	-0.19	1.27	72698	7601	77383	13755	0.37299	0.40	0.67
700.55316@12.049995	700.5532	12.05	RP	POS	SM 34:2	2	2575429	771239	2933568	904633	0.37684	0.41	0.86	1252721	112797	1165636	285905	0.39344	-0.38	0.75
701.40826@10.587004	701.4083	10.59	RP	NEG			72423	9280	86226	12134	0.01364	1.07	1.18	53767	9482	42094	7628	0.01265	-1.13	1.91
702.5688@12.264006	702.5688	12.26	RP	POS			17592643	4131816	21955559	6341447	0.09630	0.74	0.84	520507	41687	562850	61383	0.09983	0.74	1.30
703.516@12.089997	703.5160	12.09	RP	POS	184 fragment in MSMS	3	606658	153704	660549	190728	0.51522	0.30	0.89	331007	39576	254764	55085	0.00314	-1.23	2.03
704.5172@10.475001	704.5172	10.48	RP	NEG										179923	47540	168959	23686	0.56318	-0.30	0.69
705.4396@10.791994	705.4396	10.79	RP	NEG			97437	11803	107310	12156	0.09955	0.76	1.02	22014	6398	18304	6255	0.23370	-0.56	1.20
705.5326@12.234993	705.5326	12.23	RP	POS	PC 30:0	2	899312	174836	997636	248037	0.33808	0.44	0.90	140952	16800	143757	25290	0.78161	0.13	0.59
709.4694@11.058005	709.4694	11.06	RP	NEG			35661	5880	43564	7064	0.01851	1.03	1.11	39720	4563	37200	6807	0.36170	-0.42	0.71
712.4123@10.528998	712.4123	10.53	RP	NEG			63316	15108	79394	14671	0.03639	0.95	1.07	72688	16757	63311	17473	0.26270	-0.53	0.98
713.46436@10.731002	713.4644	10.73	RP	NEG										81869	9381	76092	10682	0.23822	-0.55	1.00
713.46747@11.058005	713.4675	11.06	RP	NEG			37494	8423	47232	10907	0.04660	0.89	1.06	39718	7883	38353	9679	0.74536	-0.15	0.69
713.49866@11.919004	713.4987	11.92	RP	POS	PE 34:3	2	666685	116335	726023	141977	0.34272	0.44	0.63	68678	12922	58875	8722	0.08840	-0.83	1.42
713.4989@11.947999	713.4989	11.95	RP	NEG	PE 16:1_18:2	2	1189490	252580	1190662	210640	0.99174	0.01	0.73	1031415	152815	794252	133242	0.00322	-1.28	2.07
714.00085@6.2010016	714.0009	6.20	HILIC	NEG										65161	12786	64004	19653	0.88206	-0.07	0.67
715.4168@10.546998	715.4168	10.55	RP	NEG			79924	26745	121440	35178	0.01080	1.10	1.17	100179	28325	79858	22919	0.12068	-0.74	1.23
715.5129@12.436998	715.5129	12.44	RP	NEG										102514	19279	88090	29944	0.23294	-0.54	1.06
715.51373@12.304995	715.5137	12.30	RP	NEG	PE 16:0_18:2	2	1316840	180592	1114494	310402	0.10259	-0.73	0.82	135889	26343	116921	29739	0.16914	-0.64	1.04
715.51794@12.308996	715.5179	12.31	RP	POS	PE 34:2	2	1050951	141855	944135	211346	0.21783	-0.56	0.90	28396	4499	29055	3848	0.74677	0.16	0.39
716.10767@6.5549994	716.1077	6.55	HILIC	NEG			41832	8348	40866	7110	0.79789	-0.13	0.66							
717.53406@12.234993	717.5341	12.23	RP	POS			1680506	221254	1607669	271572	0.53852	-0.29	0.55	272241	33889	259468	26992	0.39885	-0.41	0.75
717.5373@11.630001	717.5373	11.63	RP	POS										253815	74480	171662	63969	0.02508	-1.03	1.66
719.55286@11.731998	719.5529	11.73	RP	POS										77299	25613	57863	18993	0.09478	-0.80	1.30
721.52124@12.716002	721.5212	12.72	RP	POS			1053167	224657	985554	112937	0.45362	-0.38	1.00	633167	150015	689703	316607	0.62506	0.22	0.51
723.5196@12.47	723.5196	12.47	RP	NEG			1064230	279794	836662	317078	0.12373	-0.71	0.98	383473	84309	339861	123180	0.38591	-0.40	0.76
723.5224@12.5079975	723.5224	12.51	RP	POS	P-PE 36:4	2	564327	142002	451043	135265	0.10429	-0.76	0.92	47190	5821	43515	7685	0.26355	-0.51	0.98
724.5232@10.477001	724.5232	10.48	RP	NEG										104836	63913	97779	32487	0.78134	-0.14	0.62
724.5502@12.225004	724.5502	12.23	RP	POS			1032603	128047	1222048	229534	0.04144	0.89	1.02	24885	2771	26481	2841	0.24616	0.55	0.92
725.46545@10.475001	725.4655	10.48	RP	NEG										395092	91211	349502	52318	0.23219	-0.60	1.23
725.87946@4.401998	725.8795	4.40	HILIC	NEG			91816	3180	91951	4210	0.93941	0.04	0.81	90965	9376	91997	9187	0.81778	0.11	0.63
727.5293@12.47	727.5293	12.47	RP	NEG			65320	18582	51622	18608	0.13865	-0.69	0.91	200156	52658	215417	69423	0.60248	0.24	0.80
728.5843@12.368996	728.5843	12.37	RP	POS	SM 36:2	2	1758625	385239	1889573	449313	0.51433	0.31	0.61	55799	4602	49578	8362	0.06220	-0.82	1.36
729.5166@12.234003	729.5166	12.23	RP	NEG			434022	56697	391415	84903	0.22017	-0.56	0.72	466089	49715	467734	102992	0.96512	0.02	0.33
729.5288@12.143998	729.5288	12.14	RP	NEG										384082	54617	330496	80181	0.11039	-0.72	1.18
729.53204@12.052001	729.5320	12.05	RP	POS	PC 14:0_18:2	1	1350708	328749	1447730	401815	0.58011	0.26	0.96	2525791	319271	1974353	340154	0.00243	-1.28	2.08
730.5992@13.043001	730.5992	13.04	RP	POS	SM 36:1	2	2625870	788158	2888603	846814	0.50546	0.32	0.51							
731.5482@12.326997	731.5482	12.33	RP	POS			3432486	373676	3534367	638540	0.67829	0.19	0.95	1398260	262417	1211256	268528	0.15505	-0.66	1.17
733.5337@11.498	733.5337	11.50	RP	POS										82379	22425	55322	20688	0.01779	-1.07	1.73
733.5632@12.941004	733.5632	12.94	RP	POS	PC 16:0/16:0	1	1622873	641857	1517724	414568	0.69475	-0.20	1.06	646628	35642	658606	96311	0.72205	0.16	0.56
737.5001@11.852998	737.5001	11.85	RP	NEG	PE 16:1_20:4	2	773951	161600	725109	158380	0.52890	-0.30	0.66	369183	128324	300178	116337	0.25437	-0.54	0.95
737.5014@11.855995	737.5014	11.86	RP	POS			687665	154051	703083	160939	0.83854	0.10	0.41	246262	22216	209082	60435	0.09486	-0.73	1.25
739.40765@10.527006	739.4077	10.53	RP	NEG			54102	26312	93246	36700	0.01704	1.03	1.12	145698	47418	123645	41279	0.31478	-0.48	0.85
739.47876@10.796998	739.4788	10.80	RP	NEG			41710	9227	45156	14331	0.54518	0.28	0.74	340518	39929	302677	58363	0.12119	-0.70	1.29
739.5146@12.225004	739.5146	12.23	RP	NEG	PE 16:0_20:4	2	1049604	192535	843095	237493	0.05659	-0.86	1.06	566236	60855	483613	94448	0.03818	-0.91	1.56
739.5146@12.130003	739.5146	12.13	RP	NEG	PE 18:2/18:2	2	484287	99262	488335	90678	0.92991	0.04	0.23	49204	13357	48344	15135	0.89971	-0.06	0.15
739.5167@12.438002	739.5167	12.44	RP	NEG										142469	31875	135917	79220	0.81500	-0.10	0.58
739.5185@12.234993	739.5185	12.23	RP	POS	PE 36:4	2	1040908	173528	918255	204028	0.18527	-0.61	0.80	145234	18529	143012	20384	0.81172	-0.11	0.81

740.57495@10.802001	740.5750	10.80	RP	NEG										59608	11385	51680	9251	0.13156	-0.72	1.31
741.5301@12.437995	741.5301	12.44	RP	NEG	PE 18:1_18:2	2	1720711	268437	1516111	481502	0.27070	-0.50	0.74	595609	131992	609919	288332	0.89079	0.06	0.56
741.53326@12.437995	741.5333	12.44	RP	POS			1410782	188537	1335831	291468	0.51863	-0.30	1.03	89977	14583	85607	23239	0.63255	-0.22	0.47
741.5337@12.161004	741.5337	12.16	RP	POS			457071	111182	446248	114994	0.84217	-0.10	0.40	1371552	105913	1313965	135043	0.32406	-0.46	0.87
743.5412@12.437995	743.5412	12.44	RP	NEG	PC 16:0_18:2	2	246216	34882	227663	44333	0.33357	-0.45	0.67	476211	44562	464129	89099	0.71332	-0.17	0.51
743.5451@12.9920025	743.5451	12.99	RP	NEG	PE 18:1_18:1	2	662222	225321	507686	258845	0.19293	-0.60	0.84	271989	21685	278692	31813	0.60281	0.24	0.40
743.5465@12.3469925	743.5465	12.35	RP	POS			1348961	167609	1208403	220080	0.14155	-0.67	0.88	152893	8983	151236	20753	0.82343	-0.10	0.47
743.5471@12.3469925	743.5471	12.35	RP	POS			1267579	176536	1160971	226551	0.27662	-0.50	0.79	266741	30548	255029	34832	0.45764	-0.35	0.75
743.548@12.213005	743.5480	12.21	RP	POS	PC 33:2	2	205081	50344	234679	71417	0.31717	0.46	0.81	966938	119120	936179	99844	0.56734	-0.28	0.58
744.50653@10.494999	744.5065	10.49	RP	POS										69499	10139	71094	11433	0.75760	0.15	0.62
745.5594@12.990999	745.5594	12.99	RP	NEG	PC 16:0_18:1	2	93573	26663	84576	21705	0.45213	-0.37	1.01	304208	19087	296497	29785	0.51455	-0.30	0.53
745.56244@12.634002	745.5624	12.63	RP	POS			295074	63416	300902	86844	0.87128	0.08	1.29	789804	108613	690597	163795	0.14185	-0.66	1.17
745.58374@12.903997	745.5837	12.90	RP	POS			370695	72935	391134	68014	0.55098	0.29	0.74	1048827	91230	934822	106451	0.02504	-0.99	1.67
745.96106@10.4609995	745.9611	10.46	RP	NEG			215061	8637	214493	11735	0.90719	-0.05	0.80	234011	27679	228624	23351	0.66638	-0.21	0.88
747.5216@12.411995	747.5216	12.41	RP	NEG			342782	74645	305834	103737	0.39186	-0.39	0.67	1050751	206922	995208	307663	0.65379	-0.21	0.62
747.5218@12.389006	747.5218	12.39	RP	POS			271915	52831	238945	68220	0.26306	-0.52	0.83	57643	8673	62962	10248	0.24868	0.54	0.88
748.5479@11.714002	748.5479	11.71	RP	NEG										49874	9571	37127	11990	0.02193	-1.01	1.63
751.515@12.031004	751.5150	12.03	RP	POS			249034	41427	252058	43468	0.88207	0.07	1.03	276887	26732	225350	30258	0.00124	-1.34	2.16
751.53564@12.208006	751.5356	12.21	RP	NEG			312121	86909	326202	92154	0.74347	0.16	1.17	72729	8943	70648	11047	0.66394	-0.20	0.58
753.52985@11.989006	753.5299	11.99	RP	POS	PC 14:0_20:4 (PC 34:4)	2	389088	108785	430713	151030	0.50599	0.31	1.11	1885090	235205	1389239	269693	0.00059	-1.40	2.26
753.5313@12.438002	753.5313	12.44	RP	NEG										54948	23112	68734	71741	0.57766	0.25	0.72
755.54645@10.570005	755.5465	10.57	RP	POS			608142	209168	754814	203095	0.15270	0.67	0.81	303224	90046	256773	87182	0.28550	-0.51	0.99
755.548@12.138	755.5480	12.14	RP	POS	PC 16:1_18:2	1	3104002	614324	3391488	744581	0.38134	0.41	0.83	12613911	1139900	10551194	1429061	0.00315	-1.24	2.02
756.5372@12.320003	756.5372	12.32	RP	NEG			752324	109411	865937	168405	0.10213	0.73	0.86							
757.5643@12.479003	757.5643	12.48	RP	POS	PC 16:0_18:2	1	24703422	2734583	26520711	3871515	0.25948	0.52	0.69	70624688	4091434	70295600	3900225	0.86463	-0.08	0.21
758.41095@10.526	758.4110	10.53	RP	NEG			16108	6679	23035	5722	0.03351	0.98	1.08	46829	11388	44290	13102	0.66566	-0.20	0.65
759.5796@13.039	759.5796	13.04	RP	POS	PC 34:1	2	10928233	2332188	11232266	2749723	0.80260	0.12	0.77	45819392	3562431	44198819	5774302	0.47501	-0.33	0.65
760.5803@10.802998	760.5803	10.80	RP	NEG										95575	32748	86613	13272	0.48333	-0.37	0.92
761.4989@11.785003	761.4989	11.79	RP	NEG	PE 16:1_22:6	2	194257	36965	193259	41560	0.95767	-0.03	0.39	271738	41939	231995	72765	0.16532	-0.62	1.02
761.50287@11.793	761.5029	11.79	RP	POS			154294	35250	169261	40564	0.41373	0.38	0.53	222721	31901	186838	59888	0.12325	-0.68	1.15
763.5147@11.968996	763.5147	11.97	RP	NEG	PC 14:0_22:6	1	363488	67534	333577	68800	0.36722	-0.43	0.73	810203	156762	647306	184229	0.05793	-0.86	1.47
763.5151@12.437995	763.5151	12.44	RP	NEG			49712	7669	49439	13000	0.95643	-0.02	1.58	232279	71749	202629	81664	0.42355	-0.38	0.91
763.5162@12.143003	763.5162	12.14	RP	NEG	PE 18:2_20:4	2	620888	110028	536432	150232	0.18600	-0.60	0.76	1648894	168201	1501300	277371	0.18179	-0.60	1.06
763.5167@12.153006	763.5167	12.15	RP	POS	PE 16:0_22:6	1	499229	106605	454084	113992	0.39836	-0.40	0.60	765423	68710	748912	109617	0.70131	-0.18	0.64
765.53064@12.338995	765.5306	12.34	RP	NEG	PE 18:1_20:4	2	1816464	309774	1465756	434176	0.06138	-0.83	0.97	468109	59993	403142	172168	0.28650	-0.47	0.86
765.5309@12.436998	765.5309	12.44	RP	NEG	PE 18:1_20:4	2								835113	188355	736259	324760	0.43078	-0.36	0.82
765.53284@12.349	765.5328	12.35	RP	POS			1405886	209418	1197214	249926	0.06997	-0.82	0.94	230248	26733	224483	33654	0.69021	-0.19	0.59
765.56464@12.670996	765.5646	12.67	RP	POS			622378	290931	585020	377329	0.81523	-0.11	1.31	394849	55365	351850	53220	0.11427	-0.74	1.26
767.54565@12.906004	767.5457	12.91	RP	NEG	PE 18:0_20:4	2	597952	248217	411052	252531	0.13336	-0.70	0.89	405441	55674	381407	61088	0.39519	-0.40	0.76
768.59143@12.920006	768.5914	12.92	RP	POS			245986	62145	266915	62137	0.48723	0.33	0.82	786188	93715	720772	91842	0.15594	-0.67	1.12
769.5304@10.802998	769.5304	10.80	RP	POS										154494	20665	158707	37811	0.76756	0.13	0.61
769.5581@12.295006	769.5581	12.30	RP	POS			205297	22218	212518	12875	0.43013	0.40	1.31	362116	41230	392184	46085	0.16208	0.65	1.07
771.58295@12.899996	771.5830	12.90	RP	POS	PC 35:2	2	1465698	244644	1550979	239535	0.46838	0.35	0.45	4108521	185163	4078427	583961	0.88039	-0.07	0.51
775.5355@12.044004	775.5355	12.04	RP	NEG	PC 14:0_18:2 (M+FA-H-)	2	585528	140554	591071	152593	0.93712	0.04	1.21	1292116	183984	1004635	170435	0.00350	-1.26	2.04
777.5299@12.112	777.5299	12.11	RP	POS	PC 36:6	2	573230	70778	596186	73768	0.51100	0.31	0.89	2008210	215035	1672897	293691	0.01206	-1.08	1.75
777.5472@12.436998	777.5472	12.44	RP	NEG										59481	16218	61945	35451	0.84753	0.09	0.59
777.5508@12.290003	777.5508	12.29	RP	NEG			1741752	297033	1778561	351861	0.81261	0.11	0.95	553425	97228	480659	103089	0.14240	-0.68	1.15
779.53827@11.496001	779.5383	11.50	RP	NEG										42721	7337	29308	9179	0.00289	-1.25	2.03
779.5466@12.082	779.5466	12.08	RP	POS	PC 16:1_20:4	1	1514602	341347	1625966	436767	0.55133	0.28	1.00	10483034	990370	9751242	1412914	0.21394	-0.57	0.96
779.56665@12.924995	779.5667	12.92	RP	NEG	PC 16:0/16:0	1	810856	393903	758132	264629	0.75056	-0.16	1.13	431306	36451	455473	61991	0.31768	0.45	0.82
781.5278@10.802998	781.5278	10.80	RP	NEG										810717	93174	715022	117155	0.06948	-0.82	1.37
781.5637@12.225004	781.5637	12.23	RP	POS	PC 36:4	2	1727444	385514	2067450	454790	0.10339	0.74	1.00	3065716	421656	3411383	692242	0.20935	0.57	1.14
781.5642@12.377996	781.5642	12.38	RP	POS	PC 16:0_20:4	1	10811160	1628990	11049590	2259701	0.79800	0.12	1.20	30854482	1493931	29876936	1179610	0.15139	-0.69	1.27
783.58@12.670996	783.5800	12.67	RP	POS	PC 36:3	2	22663565	3836235	24062067	3734445	0.44751	0.36	0.66	42579096	3246781	43830383	7549881	0.64385	0.21	0.68
787.5211@11.916006	787.5211	11.92	RP	NEG			162093	37494	166923	36252	0.78618	0.13	0.83	261946	27076	250676	58203	0.59505	-0.24	0.42
789.5304@11.972994	789.5304	11.97	RP	NEG	PC 16:1_22:6	2	148053	37770	163290	50856	0.47547	0.33	1.07	3244424	455527	2714307	591385	0.04560	-0.89	1.44
789.5314@12.436998	789.5314	12.44	RP	NEG										135786	26045	126929	49610	0.63306	-0.22	0.68
789.5342@12.255003	789.5342	12.26	RP	POS			288315	51148	258914	70369	0.31889	-0.46	0.93	75463	7024	73651	18075	0.77559	-0.13	0.49
789.535@12.239996	789.5350	12.24	RP	NEG	PE 18:1_22:6	2	475298	70438	434700	97222	0.31855	-0.46	0.67	609174	55899	618506	104611	0.81174	0.11	0.32
791.54456@12.136995	791.5446	12.14	RP	NEG			436126	199473	499617	228473	0.53741	0.29	1.03	2922348	404304	2464510	726689	0.10983	-0.71	1.17
791.5457@12.259002	791.5457	12.26	RP	NEG	PC 16:0_22:6	2	813461	121705	818203	167587	0.94539	0.03	1.19	13349128	1123561	13085791	1026397	0.61455	-0.24	0.74
791.55505@12.041997	791.5551	12.04	RP	POS	PC 37:6	2								523929	48653	451603	79596	0.02981	-0.95	1.53
791.564@12.596003	791.5640	12.60	RP	NEG			389103	75640	373109	67117	0.64571	-0.22	1.14	556012	46195	509447	39054	0.03740	-0.96	1.56

793.546@12.468996	793.5460	12.47	RP	NEG			338047	28647	317134	23117	0.11452	-0.75	1.43	358806	27276	362718	55352	0.84739	0.09	0.62
793.59393@13.010994	793.5939	13.01	RP	POS	PC-P 38:4	2	261648	58201	270258	55687	0.75447	0.15	0.97	1463605	159596	1397847	173373	0.41443	-0.39	0.77
795.51184@12.489996	795.5118	12.49	RP	POS			429317	9170	433935	25240	0.60086	0.23	0.92	479877	54491	505847	85401	0.44436	0.35	0.82
795.5789@12.766999	795.5789	12.77	RP	POS			780444	98824	773554	139133	0.90377	-0.06	1.13	2044343	182766	1753272	248488	0.01043	-1.10	1.80
795.9575@10.988005	795.9575	10.99	RP	NEG			375918	41041	387482	42435	0.56601	0.27	1.08	541666	69190	545604	76100	0.90992	0.05	0.81
797.52655@13.032005	797.5266	13.03	RP	POS	PC 37:3	2	259472	32971	262881	33608	0.83145	0.10	0.69	395766	49880	419363	49425	0.33063	0.46	0.98
799.5359@11.967004	799.5359	11.97	RP	NEG	PC 14:0_20:4 (M+FA-H-)	2	192241	50291	211773	71232	0.50490	0.31	1.17	985014	156185	737471	119220	0.00221	-1.34	2.17
801.5269@12.0390005	801.5269	12.04	RP	POS			293414	48424	304171	55828	0.66703	0.20	1.07	775685	75444	732855	91971	0.29212	-0.49	0.85
801.55176@12.135994	801.5518	12.14	RP	NEG	PC 16:1_18:2 (M+FA-H-)	2	1448806	329599	1516773	335836	0.67173	0.20	1.01	6783634	811615	5644851	748939	0.00727	-1.18	1.91
803.5468@12.0	803.5468	12.00	RP	POS	PC 38:7	2	166972	59345	189231	70446	0.47638	0.33	0.93	5930635	657316	5176149	863893	0.04977	-0.87	1.42
803.5675@12.458006	803.5675	12.46	RP	NEG	PC 16:0/18:2	1	13328200	1865797	13765486	2240554	0.65688	0.21	0.80	40137716	3575769	39842232	1883083	0.83624	-0.11	0.48
805.56116@12.208006	805.5612	12.21	RP	NEG			1507780	226988	1582704	356746	0.59533	0.24	1.15	17071754	1453560	16675182	1407558	0.56739	-0.28	0.75
805.56134@12.735994	805.5613	12.74	RP	POS	PC 38:6	2	2202163	75192	2226869	110553	0.58086	0.25	1.61	3519603	271608	3480556	302970	0.77679	-0.13	0.64
805.56305@12.286005	805.5631	12.29	RP	POS	PC 16:0_22:6	1	1687298	216936	1614150	240850	0.50730	-0.31	1.58	28236362	1699003	27246039	1516834	0.21575	-0.59	1.03
805.56354@12.143003	805.5635	12.14	RP	POS	PC 38:6	2	1018245	351120	1266189	493675	0.22998	0.55	0.88	4959821	689464	4253686	1344536	0.16947	-0.61	1.03
805.5826@13.020998	805.5826	13.02	RP	NEG	PC 16:0_18:1 (M+FA-H-)	2	5774546	1631812	5639252	1710319	0.86612	-0.08	1.01	26569242	1984263	25645599	2807716	0.42519	-0.37	0.64
807.5808@12.519996	807.5808	12.52	RP	POS	PC 18:1_20:4	2	6724710	952239	6474556	1030972	0.60016	-0.25	1.23	19912581	2031598	17624127	2043444	0.02983	-0.98	1.59
809.5931@12.927	809.5931	12.93	RP	POS	PC 38:4	2	815507	239196	822678	217863	0.94838	0.03	0.52	3528259	852502	4047336	1398648	0.34582	0.43	0.91
809.5955@13.1530075	809.5955	13.15	RP	POS	PC 18:0_20:4	1	9440204	2501095	8403895	2474361	0.39231	-0.41	1.43	28032533	2461601	23918370	2806679	0.00393	-1.23	2.02
812.0063@10.724	812.0063	10.72	RP	POS			7781	2394	10064	5805	0.27783	0.48	0.98	278438	52822	254552	61628	0.38793	-0.40	0.79
813.5871@12.815997	813.5871	12.82	RP	NEG										621569	64563	560423	56254	0.05072	-0.91	1.55
817.31305@6.150001	817.3131	6.15	HILIC	NEG			99089	41454	90241	59670	0.71556	-0.17	1.30							
817.5632@12.130998	817.5632	12.13	RP	POS										210242	22795	212072	31390	0.88779	0.07	0.41
817.58105@12.879	817.5811	12.88	RP	NEG	PC 17:0_18:2	2	534539	108432	543645	117360	0.86644	0.08	0.62	1847431	106003	1842101	219989	0.94711	-0.03	0.49
817.59814@12.823	817.5981	12.82	RP	POS										363183	52831	315667	69788	0.11769	-0.71	1.19
818.59265@10.453002	818.5927	10.45	RP	NEG			52751	13394	61696	10932	0.14746	0.69	0.89	21903	6221	17058	6081	0.11566	-0.73	1.41
819.51117@12.365998	819.5112	12.37	RP	POS			180074	18717	179708	24052	0.97150	-0.02	1.03	135024	11758	140960	27077	0.54273	0.27	0.66
821.5281@12.734003	821.5281	12.73	RP	POS			512202	49627	526228	47219	0.55085	0.29	0.85	483491	23317	499265	48294	0.37770	0.40	0.80
821.63116@10.729003	821.6312	10.73	RP	NEG			52164	5448	56293	3841	0.09146	0.81	1.06	19856	5280	16872	2962	0.17875	-0.67	1.31
823.5425@12.028997	823.5425	12.03	RP	NEG			156558	19063	164359	29913	0.51077	0.30	0.99	967213	103822	788234	133459	0.00497	-1.19	1.93
824.63904@10.730996	824.6390	10.73	RP	NEG			50544	5576	51523	5503	0.71417	0.18	1.11	26776	2669	23726	4817	0.10794	-0.71	1.27
825.55164@12.181	825.5516	12.18	RP	NEG			1146921	380092	1273763	417389	0.50939	0.31	1.04	7504861	625480	7043375	1246301	0.32297	-0.44	0.81
827.546@11.897002	827.5460	11.90	RP	POS										330071	64045	286673	69644	0.18666	-0.62	1.03
827.5659@12.207	827.5659	12.21	RP	NEG			639092	301158	763407	324576	0.41185	0.39	0.94	1925118	215117	2026992	490185	0.56435	0.26	0.76
827.5674@12.345	827.5674	12.35	RP	NEG	PC 16:0/20:4 (M+FA-H-)	2	5175228	971423	5409824	1353264	0.67412	0.20	1.24	17963060	1233279	16793421	860161	0.03938	-0.98	1.68
829.56055@12.532005	829.5606	12.53	RP	POS			504673	36702	472485	35193	0.07713	-0.82	1.51	619966	63909	577911	47210	0.14225	-0.71	1.18
829.5613@12.072999	829.5613	12.07	RP	POS	PC 40:8	2	443926	79659	502110	135174	0.27179	0.50	1.05	4139582	527107	4259482	590350	0.65494	0.21	0.41
829.583@12.683006	829.5830	12.68	RP	NEG	PC 18:1_18:2 (M+FA-H-)	2	11466605	2381089	11549404	2276852	0.94130	0.04	0.83	23232358	1542807	24045859	3606276	0.53016	0.28	0.75
831.5745@12.359993	831.5745	12.36	RP	NEG			318345	34232	319166	42741	0.96439	0.02	1.16	1688892	157124	1769216	226719	0.38772	0.40	0.91
831.5794@12.377996	831.5794	12.38	RP	POS	PC 40:7	2	420833	30150	428866	51751	0.68612	0.18	0.83	3053899	281408	3210511	368434	0.32025	0.46	0.91
833.5921@13.002996	833.5921	13.00	RP	POS	PC 18:0_22:6	2	732837	143414	657215	135439	0.27064	-0.52	1.28	6932308	948208	5977745	609853	0.02820	-1.04	1.69
837.55115@12.039997	837.5512	12.04	RP	NEG										453998	34059	400649	47857	0.01304	-1.07	1.74
839.56366@12.203002	839.5637	12.20	RP	NEG			53028	5049	48656	12969	0.34547	-0.42	0.95	155332	15231	158673	21500	0.70469	0.18	0.45
839.60126@13.006996	839.6013	13.01	RP	NEG	PC P-18:0_20:4 (M+FA-H-)	2	120746	30515	122004	26813	0.92808	0.04	1.14	602616	68314	568127	73420	0.31721	-0.47	0.87
841.5424@12.201998	841.5424	12.20	RP	NEG										179434	14945	195110	34925	0.22094	0.54	1.01
842.4969@10.802001	842.4969	10.80	RP	NEG										114313	14350	102178	18243	0.13162	-0.69	1.21
844.4969@10.802001	844.4969	10.80	RP	NEG										110194	14440	98855	17694	0.15174	-0.66	1.16
845.9546@11.648004	845.9546	11.65	RP	NEG			542880	24752	540692	28549	0.86386	-0.08	1.11	766519	97748	756735	97819	0.83541	-0.10	0.63
847.6113@10.451997	847.6113	10.45	RP	NEG			56121	14784	68198	10392	0.07093	0.87	0.95	6278	3044	4343	2093	0.14849	-0.71	1.31
849.51776@10.802998	849.5178	10.80	RP	NEG										56560	9506	50780	8562	0.22810	-0.61	1.07
849.5469@12.418	849.5469	12.42	RP	NEG			283530	27349	291121	45809	0.66854	0.20	0.95							
849.5853@10.526	849.5853	10.53	RP	NEG			21696	6392	30809	9785	0.02912	0.95	1.08	161492	22898	147332	23778	0.21682	-0.58	1.17
853.5616@12.005003	853.5616	12.01	RP	POS	PC 42:10	2	135813	26599	159950	50374	0.21065	0.56	0.99	1789949	278755	1893270	389031	0.52056	0.30	0.52
853.57513@12.276	853.5751	12.28	RP	NEG			145966	43771	152815	46636	0.75246	0.15	1.13	1994621	160124	1946364	221144	0.59835	-0.24	0.65
853.58295@12.510004	853.5830	12.51	RP	NEG	PC 18:1_20:4 (M+FA-H-)	2	3772063	662178	3626609	650544	0.64663	-0.22	1.39	11049005	1135030	9812384	961988	0.02613	-1.02	1.65
853.65265@10.727998	853.6527	10.73	RP	NEG			67901	7807	72085	4688	0.20559	0.63	0.77	19753	5194	17668	3036	0.33382	-0.49	1.17
854.71484@10.457	854.7148	10.46	RP	POS			90021	15866	94361	11496	0.52653	0.31	0.57							
855.599@13.139005	855.5990	13.14	RP	NEG	PC 18:0_20:4 (M+FA-H-)	1	3904239	1360950	3499803	1290583	0.53020	-0.30	1.46	16725357	1544022	14149665	1509533	0.00246	-1.29	2.10
861.58856@10.455001	861.5886	10.46	RP	NEG			51169	12523	60021	8217	0.10797	0.79	0.93							
862.6781@12.438998	862.6781	12.44	RP	NEG										70367	18395	65105	32785	0.67287	-0.19	0.82
863.5658@12.113999	863.5658	12.11	RP	NEG										95093	9338	95458	17144	0.95486	0.03	0.71
863.60034@12.809003	863.6003	12.81	RP	NEG										194223	23126	190506	26938	0.75654	-0.15	0.51
865.4387@4.463	865.4387	4.46	RP	POS			46133	13843	42467	11283	0.55382	-0.29	0.52							

865.58344@12.520002	865.5834	12.52	RP	NEG										600692	50073	568762	48915	0.19218	-0.61	1.06
869.54065@12.109993	869.5407	12.11	RP	NEG			159034	23700	164844	20558	0.59138	0.26	0.88	294024	31725	271817	18294	0.10363	-0.81	1.35
871.55475@12.489006	871.5548	12.49	RP	NEG			1033155	55206	1073931	82889	0.22861	0.55	1.08	908571	119266	987625	261044	0.40748	0.37	0.85
872.6896@10.464998	872.6896	10.46	RP	POS			85591	18234	91499	14234	0.46414	0.36	0.51							
873.5698@13.020998	873.5698	13.02	RP	NEG			570464	108528	559834	137907	0.85685	-0.08	0.92	1118111	89823	1152025	141574	0.54479	0.28	0.71
874.68823@10.464998	874.6882	10.46	RP	POS			42420	8859	44814	7601	0.55278	0.29	0.44							
875.10236@5.330998	875.1024	5.33	HILIC	NEG			80599	5141	83948	7485	0.27690	0.50	0.94	1886	355	2459	1505	0.26774	0.49	0.85
875.5668@12.0599985	875.5668	12.06	RP	NEG			201618	36173	227022	53572	0.24732	0.53	1.05	2155625	256065	2195407	346562	0.78280	0.13	0.36
879.5964@12.976006	879.5964	12.98	RP	NEG	PC 18:0_22:6	2	380860	69200	351455	104427	0.48379	-0.32	1.55	4046466	628418	3437937	345070	0.03047	-1.05	1.71
884.6839@10.587004	884.6839	10.59	RP	NEG			45295	10123	58697	13729	0.02854	0.96	1.05	20586	5040	14388	5413	0.02218	-1.02	1.76
885.3012@6.1419992	885.3012	6.14	HILIC	NEG			25277	5983	21740	4378	0.18309	-0.65	1.61							
888.6227@11.916997	888.6227	11.92	RP	NEG										101108	20428	77953	31193	0.07501	-0.79	1.35
889.5717@10.475001	889.5717	10.48	RP	POS										158197	15445	153361	14229	0.50383	-0.32	0.53
889.5733@10.393999	889.5733	10.39	RP	POS										66963	10220	67090	7440	0.97697	0.01	0.28
890.73004@10.794004	890.7300	10.79	RP	NEG			52454	9081	60213	10153	0.10488	0.74	0.96	7435	3292	4775	1948	0.06567	-0.90	1.61
895.95154@12.352998	895.9515	12.35	RP	NEG			662659	44670	664680	46124	0.92616	0.04	1.01	963200	209848	967371	200882	0.96644	0.02	0.67
896.6404@10.587004	896.6404	10.59	RP	NEG			91941	15481	108003	15501	0.04295	0.92	1.05	38215	8917	27985	8332	0.02371	-1.02	1.78
897.5696@12.726995	897.5696	12.73	RP	NEG			1051047	120588	1054880	139413	0.95086	0.03	0.71	908315	46091	966276	70723	0.05120	0.86	1.53
899.56824@11.983001	899.5682	11.98	RP	NEG			56030	12729	64949	19460	0.25788	0.51	0.92	933714	128198	971066	212190	0.65050	0.21	0.35
899.679@10.793	899.6790	10.79	RP	NEG			134069	20570	148463	14300	0.11540	0.76	0.89	5461	1550	4276	1298	0.10322	-0.77	1.50
901.57745@12.141003	901.5775	12.14	RP	NEG										125175	19603	157117	26892	0.00937	1.11	1.86
901.70795@10.464998	901.7080	10.46	RP	POS			37292	6645	39085	6915	0.58341	0.26	0.53							
902.68646@10.794004	902.6865	10.79	RP	NEG			114809	14709	128020	18280	0.10654	0.73	0.99	43017	6548	37022	6910	0.07641	-0.81	1.50
903.4614@4.6760006	903.4614	4.68	RP	POS										181254	44897	198048	67362	0.53547	0.28	0.58
918.63477@12.438002	918.6348	12.44	RP	NEG										80217	13385	71875	23196	0.35307	-0.42	0.93
919.5525@12.173003	919.5525	12.17	RP	NEG			151917	14707	147629	17193	0.57580	-0.26	1.59	348971	22439	376927	45933	0.11165	0.70	1.26
925.65753@10.587004	925.6575	10.59	RP	NEG			106145	22561	131817	22158	0.02712	1.00	1.07	22250	6250	16037	5008	0.03710	-0.97	1.68
931.70483@10.791994	931.7048	10.79	RP	NEG			163640	21239	176034	16115	0.19273	0.63	0.76	6188	2545	5034	2117	0.31927	-0.48	1.07
933.6333@10.936003	933.6333	10.94	RP	POS			33606	6070	35391	3324	0.46972	0.37	0.84	41620	10756	55705	25401	0.13516	0.66	1.20
939.6362@10.5859995	939.6362	10.59	RP	NEG			97610	18988	121430	17972	0.01509	1.09	1.17	27022	7764	19997	4831	0.04388	-0.97	1.70
945.9485@13.105003	945.9485	13.11	RP	NEG			635991	47992	637029	57817	0.96726	0.02	0.98	1172293	274644	1178044	262991	0.96465	0.02	0.67
948.61664@10.194	948.6166	10.19	RP	NEG			22930	4959	31107	14199	0.11378	0.69	0.95	293307	26057	290470	37326	0.85190	-0.09	0.58
948.6179@10.217003	948.6179	10.22	RP	POS			4804	1394	6661	3741	0.17046	0.60	1.03	100349	6869	98175	12574	0.64700	-0.21	0.74
974.63086@10.1849985	974.6309	10.18	RP	NEG			8363	1881	11828	4733	0.05327	0.84	0.99	55076	5712	57147	9438	0.57297	0.26	0.68
974.63306@10.282997	974.6331	10.28	RP	NEG			61671	9670	78122	25224	0.07971	0.77	0.99	159729	14953	184300	29364	0.03575	0.91	1.50
990.667@10.239997	990.6670	10.24	RP	POS			226593	99007	394718	380977	0.20575	0.56	0.99	26423281	1311125	25627993	2897277	0.45099	-0.34	0.62
992.6831@10.095	992.6831	10.10	RP	POS			2324	946	6107	6417	0.09415	0.73	0.93	3040354	239320	3105533	345747	0.64309	0.21	0.51
994.6015@9.903005	994.6015	9.90	RP	NEG			2054	555	2631	730	0.07283	0.81	0.98	51705	9648	40684	7959	0.01977	-1.07	1.72
995.94525@13.948	995.9453	13.95	RP	NEG			584519	39466	586741	45623	0.91307	0.05	1.00	1093250	115561	1088623	114847	0.93353	-0.04	0.53
996.61646@10.016999	996.6165	10.02	RP	NEG			4800	960	7645	3692	0.03799	0.90	1.01	116091	23068	115611	16968	0.96144	-0.02	0.51
1000.64825@10.300997	1000.6483	10.30	RP	NEG			11857	1764	13303	2795	0.19867	0.58	0.84	101604	12566	107539	16611	0.39970	0.39	0.65
1000.65063@10.018001	1000.6506	10.02	RP	POS										204877	30151	213165	22738	0.52924	0.31	0.55
1012.6447@10.260996	1012.6447	10.26	RP	POS			134373	41534	182021	107412	0.21953	0.54	1.05	4981413	440761	4919186	404836	0.76154	-0.15	0.33
1012.6503@9.923002	1012.6503	9.92	RP	POS										516786	117283	502724	122550	0.80721	-0.12	0.23
1014.6671@10.095	1014.6671	10.10	RP	POS			3325	1203	7670	7512	0.10012	0.72	0.93	3031227	267675	3149197	360468	0.43631	0.36	0.73
1016.67395@10.230997	1016.6740	10.23	RP	POS			54761	18290	87212	63579	0.15031	0.63	1.01	1846686	290152	1886318	313925	0.78453	0.13	0.25
1016.6777@10.087004	1016.6777	10.09	RP	POS			4868	1264	8526	6456	0.10797	0.70	0.89	8521648	780264	8820656	981888	0.48083	0.33	0.60
1018.6009@9.985	1018.6009	9.99	RP	NEG										37285	6191	29197	5667	0.01114	-1.13	1.85
1018.60144@9.893001	1018.6014	9.89	RP	NEG										68915	16460	51977	11776	0.02795	-1.03	1.67
1020.6178@10.014006	1020.6178	10.01	RP	NEG			4313	1063	7805	3770	0.01630	1.04	1.10	245886	33554	220903	30434	0.12109	-0.73	1.22
1024.6495@10.007999	1024.6495	10.01	RP	POS										146987	15093	143815	22677	0.72694	-0.16	0.33
1026.6614@10.128999	1026.6614	10.13	RP	POS										126293	15748	122610	13243	0.60455	-0.25	0.49
1028.7805@10.475999	1028.7805	10.48	RP	POS										436047	59539	420641	74033	0.63050	-0.23	0.62
1030.6969@10.4140005	1030.6969	10.41	RP	POS										640382	109452	618825	121368	0.69718	-0.18	0.60
1032.6384@9.882999	1032.6384	9.88	RP	NEG										141227	42361	117423	20354	0.17237	-0.69	1.17
1032.7136@10.621001	1032.7136	10.62	RP	POS										203733	16101	190154	28733	0.22320	-0.55	0.96
1036.6697@10.221001	1036.6697	10.22	RP	NEG			432998	96127	524351	217783	0.25349	0.51	0.99	6807012	369666	6598728	399331	0.26709	-0.52	0.92
1036.8416@10.302001	1036.8416	10.30	RP	POS										41181	17229	40036	17141	0.88993	-0.07	0.58
1038.7391@10.475001	1038.7391	10.48	RP	NEG										50131	17765	42155	7047	0.25894	-0.59	1.28
1044.585@10.251005	1044.5850	10.25	RP	POS			87529	18957	105168	37309	0.21264	0.56	1.04	343199	48446	333543	31903	0.63510	-0.24	0.75
1044.6178@10.005001	1044.6178	10.01	RP	NEG										326165	36217	276693	32760	0.00828	-1.17	1.90
1044.7137@10.621001	1044.7137	10.62	RP	POS										781996	65932	710629	113464	0.11415	-0.70	1.16
1046.7296@10.724	1046.7296	10.72	RP	POS			6913947	1632187	8756875	3720504	0.18090	0.59	1.03	55172914	3261251	51216972	5852026	0.08817	-0.75	1.27

1052.5648@8.520998	1052.5648	8.52	RP	NEG			38248	48330	82149	63998	0.11481	0.71	0.94							
1058.6539@9.928995	1058.6539	9.93	RP	NEG			6036	1424	7735	1783	0.03735	0.92	1.08	238082	33148	240849	44209	0.88113	0.07	0.16
1058.7222@10.634001	1058.7222	10.63	RP	POS										163309	20957	157250	34551	0.65212	-0.21	0.41
1060.6699@10.092997	1060.6699	10.09	RP	NEG			11729	2205	19131	9321	0.03287	0.93	1.03	977334	84112	980852	64378	0.92356	0.05	0.72
1062.6669@10.0339985	1062.6669	10.03	RP	POS										6547006	604464	6331075	660927	0.47952	-0.33	0.57
1062.6842@10.200998	1062.6842	10.20	RP	NEG			97357	17456	117239	33387	0.12389	0.68	0.99	481816	114763	522580	165356	0.54582	0.28	0.49
1062.8556@10.311998	1062.8556	10.31	RP	POS										63630	17939	60244	16892	0.68818	-0.19	0.42
1066.1263@10.065999	1066.1263	10.07	RP	POS										117954	11301	121247	13693	0.58306	0.26	0.68
1068.618@9.911997	1068.6180	9.91	RP	NEG			1992	669	2303	435	0.27627	0.54	0.89	51295	13429	46193	16802	0.48321	-0.33	0.65
1068.6188@10.003999	1068.6188	10.00	RP	NEG										215893	31485	173240	20321	0.00552	-1.27	2.07
1078.1273@10.048998	1078.1273	10.05	RP	POS										108468	6661	110320	11321	0.67125	0.19	0.62
1084.6681@9.9349985	1084.6681	9.93	RP	NEG			51828	20367	62813	11307	0.19720	0.65	0.97	284877	97939	347941	162918	0.32407	0.45	0.77
1084.67@10.070005	1084.6700	10.07	RP	NEG			9440	1736	16154	8699	0.03639	0.91	1.02	2419461	203004	2483708	234384	0.54134	0.29	0.73
1088.7017@10.372995	1088.7017	10.37	RP	NEG			22881	3181	29736	10132	0.06572	0.80	0.98	3350776	421385	3158294	459359	0.36741	-0.42	0.78
1092.6171@9.996001	1092.6171	10.00	RP	NEG										96405	15559	79010	10387	0.01732	-1.12	1.83
1092.7325@10.674996	1092.7325	10.67	RP	NEG			103092	33293	117981	56958	0.49853	0.31	1.05	3622896	292197	3227836	560596	0.07251	-0.79	1.29
1108.6681@9.933004	1108.6681	9.93	RP	NEG			29320	9510	33716	6518	0.28430	0.53	0.75	277270	93681	331335	169445	0.40331	0.38	0.67
1108.6703@10.052001	1108.6703	10.05	RP	NEG			6641	1250	9172	3329	0.04466	0.87	1.00	1675115	97684	1625881	113584	0.33629	-0.45	0.77
1110.6608@9.923998	1110.6608	9.92	RP	POS										108038	26287	101836	34136	0.66845	-0.20	0.49
1110.667@10.015	1110.6670	10.02	RP	POS										1261884	239195	1149572	219164	0.31922	-0.48	0.84
1122.8331@10.802998	1122.8331	10.80	RP	NEG										64848	16751	56815	8622	0.24289	-0.59	1.32
1132.6682@9.933004	1132.6682	9.93	RP	NEG			7197	1802	8151	1755	0.27447	0.52	0.69	202233	56061	229811	109189	0.49870	0.31	0.57
1132.6715@10.016002	1132.6715	10.02	RP	NEG										890333	85758	843238	86093	0.26362	-0.53	0.86
1156.6686@9.923998	1156.6686	9.92	RP	NEG										67124	17128	72703	36256	0.67314	0.19	0.47
1156.6698@10.014003	1156.6698	10.01	RP	NEG										283006	45376	262209	45353	0.34728	-0.45	0.76
1160.7164@10.700004	1160.7164	10.70	RP	NEG			27953	7785	32841	12834	0.33298	0.44	1.00	314325	44945	289334	52172	0.28972	-0.49	0.90
1176.7819@11.826997	1176.7819	11.83	RP	NEG	Pentaerythritol tetrakis(3,5-di-tert-butyl-4-hydroxyhydrocinnamate)	2	32112	6237	30911	7194	0.70905	-0.18	0.61	106540	33540	118955	52740	0.55200	0.27	0.47
1181.9346@10.800002	1181.9346	10.80	RP	NEG			38846	6232	43267	5208	0.12809	0.72	0.89							
1193.8112@11.842004	1193.8112	11.84	RP	POS			71425	12356	70267	13268	0.85052	-0.09	1.08	190027	47573	254009	115157	0.13313	0.66	1.22
1198.7659@11.842995	1198.7659	11.84	RP	POS			55331	9473	56048	10831	0.88278	0.07	1.24	107844	42144	150714	72534	0.13597	0.67	1.23
1260.8873@10.72	1260.8873	10.72	RP	NEG										328457	259337	446546	289443	0.37424	0.42	0.90
1277.9298@10.802998	1277.9298	10.80	RP	POS										152166	45400	149721	48519	0.91357	-0.05	0.49
1410.0682@12.224	1410.0682	12.22	RP	NEG			33213	7921	38604	9149	0.19742	0.60	0.72	213827	40222	197063	43333	0.40735	-0.39	0.63
1412.0807@12.2839985	1412.0807	12.28	RP	NEG										53370	18490	78691	23101	0.01876	1.03	1.73
1434.0679@12.203999	1434.0679	12.20	RP	NEG										691231	151387	636191	102795	0.39479	-0.42	0.74
1443.9475@10.179995	1443.9475	10.18	RP	NEG			1405	635	2383	1607	0.11809	0.73	0.95	60107	5223	58482	7350	0.59091	-0.25	0.71
1485.9989@10.199999	1485.9989	10.20	RP	POS										925038	59149	871826	139601	0.29427	-0.47	0.79

Legend: d, Cohen's d effect size; DG, diglyceride; FA, fatty acid; p, p-value from Welch's t-test; PC, phosphatidyl choline; PDX, polydextrose; PE, phosphatidyl ethanolamine; PLS-DA, partial least sum of squares discriminant analysis; RT, retention time; SM, sphingomyeling; VIP, variable importance for projection.

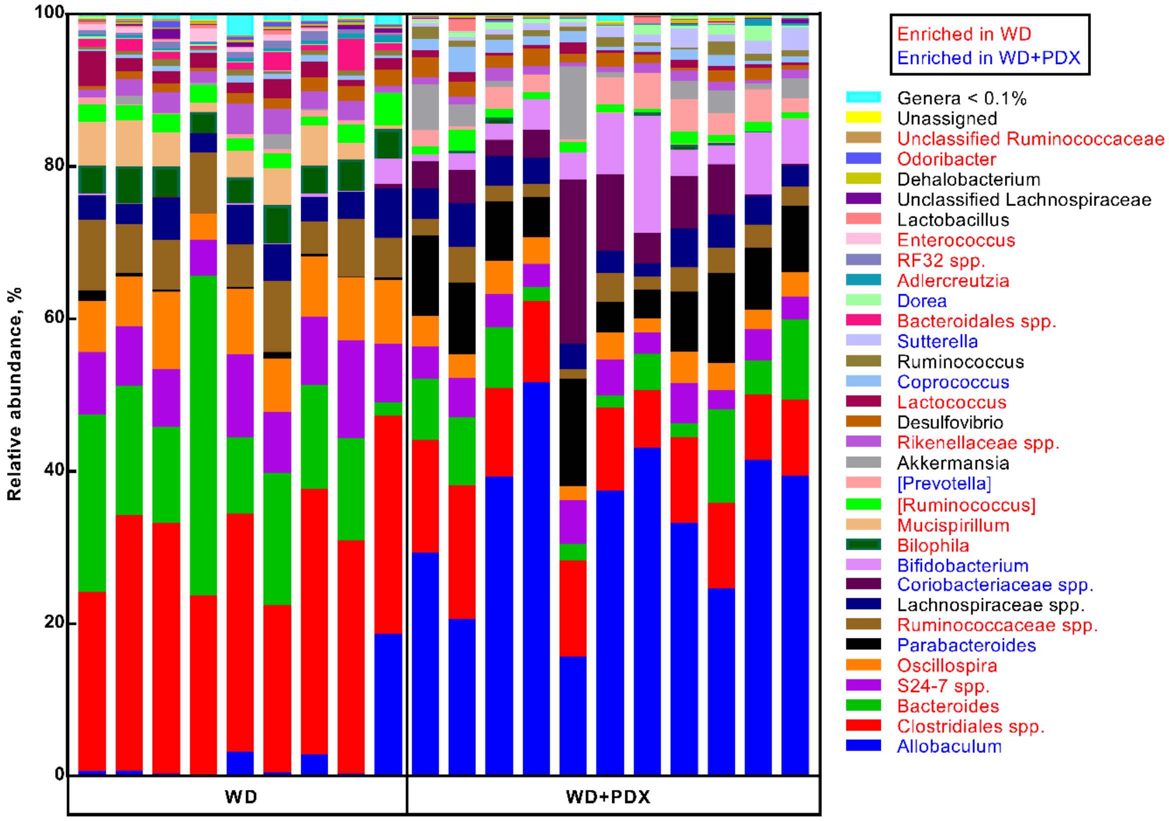

Supplementary Figure 1. Relative abundance of caecal bacteria at genus level of taxonomy in WD and WD + PDX mice. Square brackets indicate the Greengenes database notation for proposed taxonomy. Blue colored taxa were significantly enriched in WD mice and red colored taxa are significantly enriched in WD + PDX mice. $p < 0.05$; Mann-Whitney U test with false discovery rate correction (Raza, G. S. et al. Polydextrose changes the gut microbiome and attenuates fasting triglyceride and cholesterol levels in Western diet fed mice. *Sci. Rep.* **7**, 11 (2017)).
